# Supplementary material for: New HSV-1 Anti-Viral 1′-Homocarbocyclic Nucleoside Analogs with an Optically Active Substituted Bicyclo[2.2.1]Heptane Fragment as a Glycoside Moiety
Source: Molecules. 2019 Jul 3;24(13):2446. doi: 10.3390/molecules24132446 (PMC6651170; doi:10.3390/molecules24132446)
Supplement: Supplementary file 1 [file molecules-24-02446-s001.pdf]

New HSV-1 Anti-viral 1'-Homocarbocyclic Nucleoside Analogues with an Optically Active Substituted Bicyclo[2.2.1]Heptane Fragment as a Glycoside Moiety

Constantin I. Tănase[1*], Constantin Drăghici[2], Anamaria Hanganu[2], Lucia Pintilie[1], Maria Maganu[2], Alexandrina Volobueva[3], Ekaterina Sinegubova[3], Vladimir V. Zarubaev[3], Johan Neyts[4], Dirck Jochmans[4], Slita V. Alexander[3].

[1] National Institute for Chemical-Pharmaceutical Research and Development, Department of bioactive substances and pharmaceutical technologies, 112 Vitan Av., 031299, Bucharest-3, Romania

[2] Organic Chemistry Center "C.D.Nenitescu", Spectroscopy Laboratory, 202 B Splaiul Independentei, 060023 Bucharest, Romania; cst_drag@yahoo.com (C.D.).

[4] Department of Virology, Pasteur Institute of Epidemiology and Microbiology, 197101 St. Petersburg, Russia, (zarubaev@gmail.com).

[5] Rega Institute for Medicinal Research, Laboratory of Virology and Chemotherapy, KU Leuven, Herestraat 49, BE-3000 Leuven, Belgium

* Correspondence author: cvtanase@gmail.com; Tel.: +40-21-321-2117; Fax: +40-21-322-291

1. **NMR Spectra of the compounds**
2. **Molecular docking**

[1] Corresponding author. Tel.: +40-21-321.21.17; Fax: +40-21-322.29.17; e-mail: cvtanase@gmail.com

1. NMR Spectra of the compounds

1.1. ^{1}H, ^{13}C, COSY and HETCOR (aliphatic)-NMR spectra in DMSO of the compound **4a**

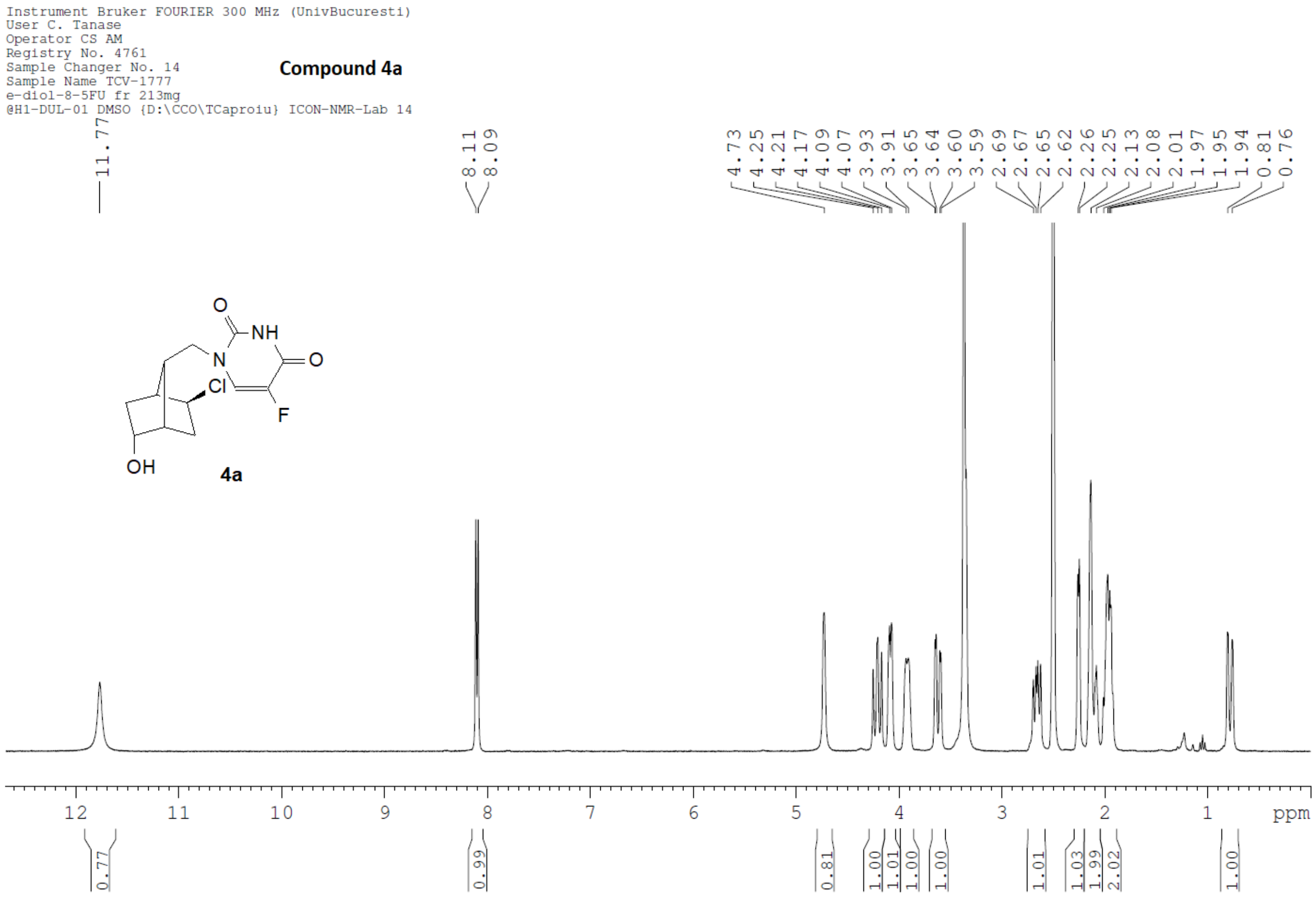

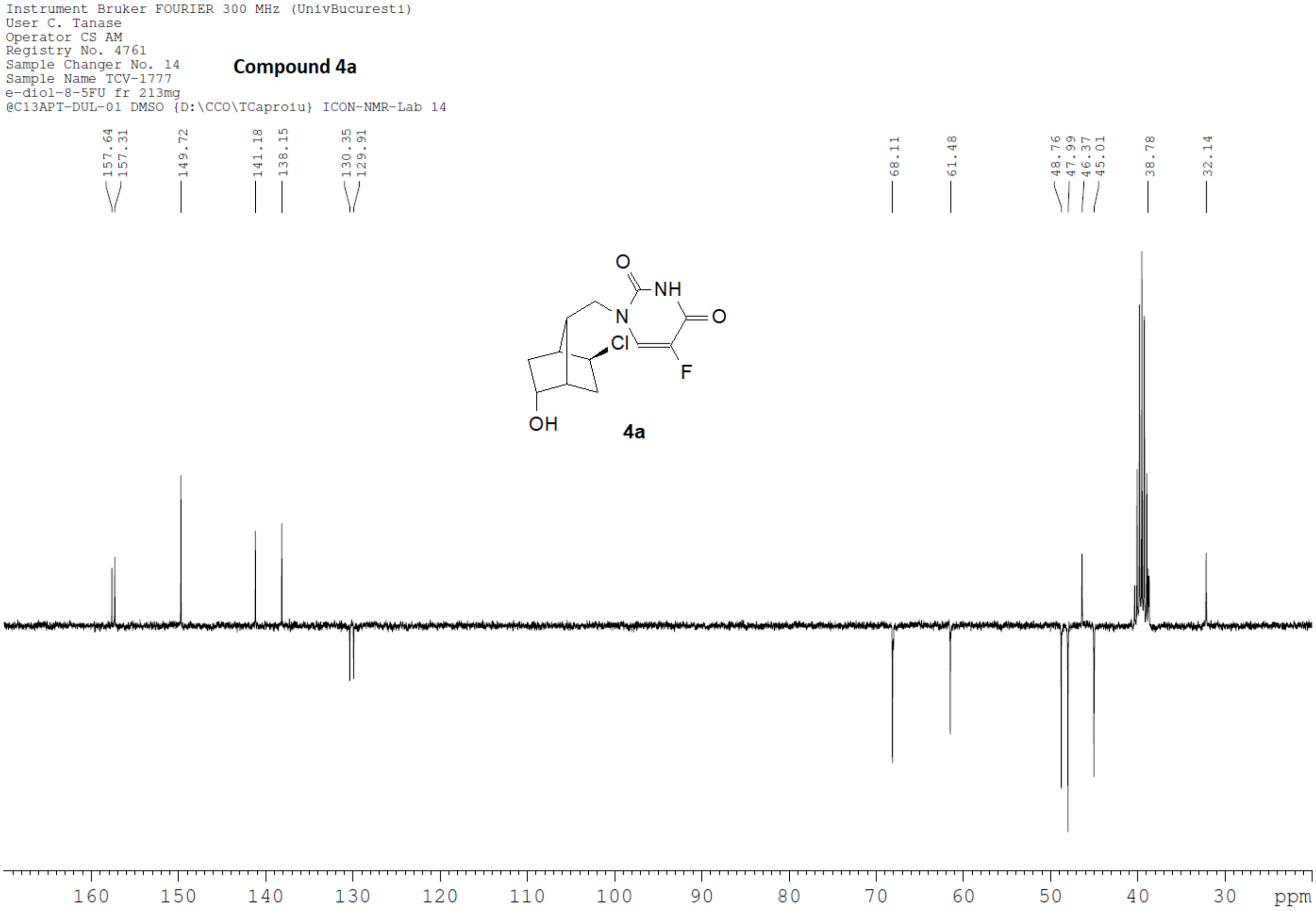

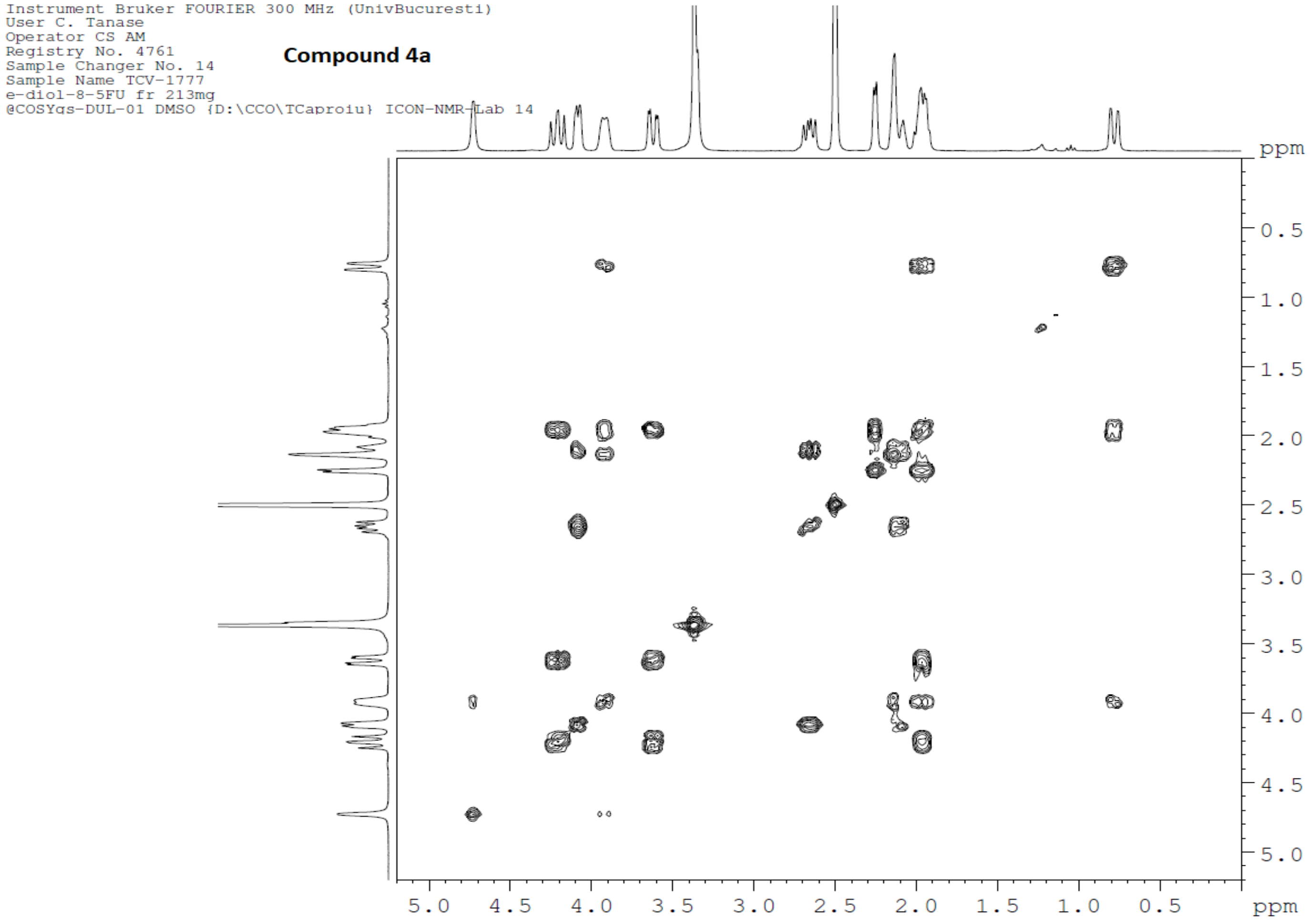
Instrument Bruker FOURIER 300 MHz (UnivBucuresti)
User C. Tanase
Operator CS AM
Registry No. 4761
Sample Changer No. 14
Sample Name TCV-1777
e-diol-8-5FU fr 213mg
@COSYgs-DUL-01 DMSO {D:\CCO\TCaproiu} ICON-NMR-Lab 14
Compound 4a
ppm
ppm

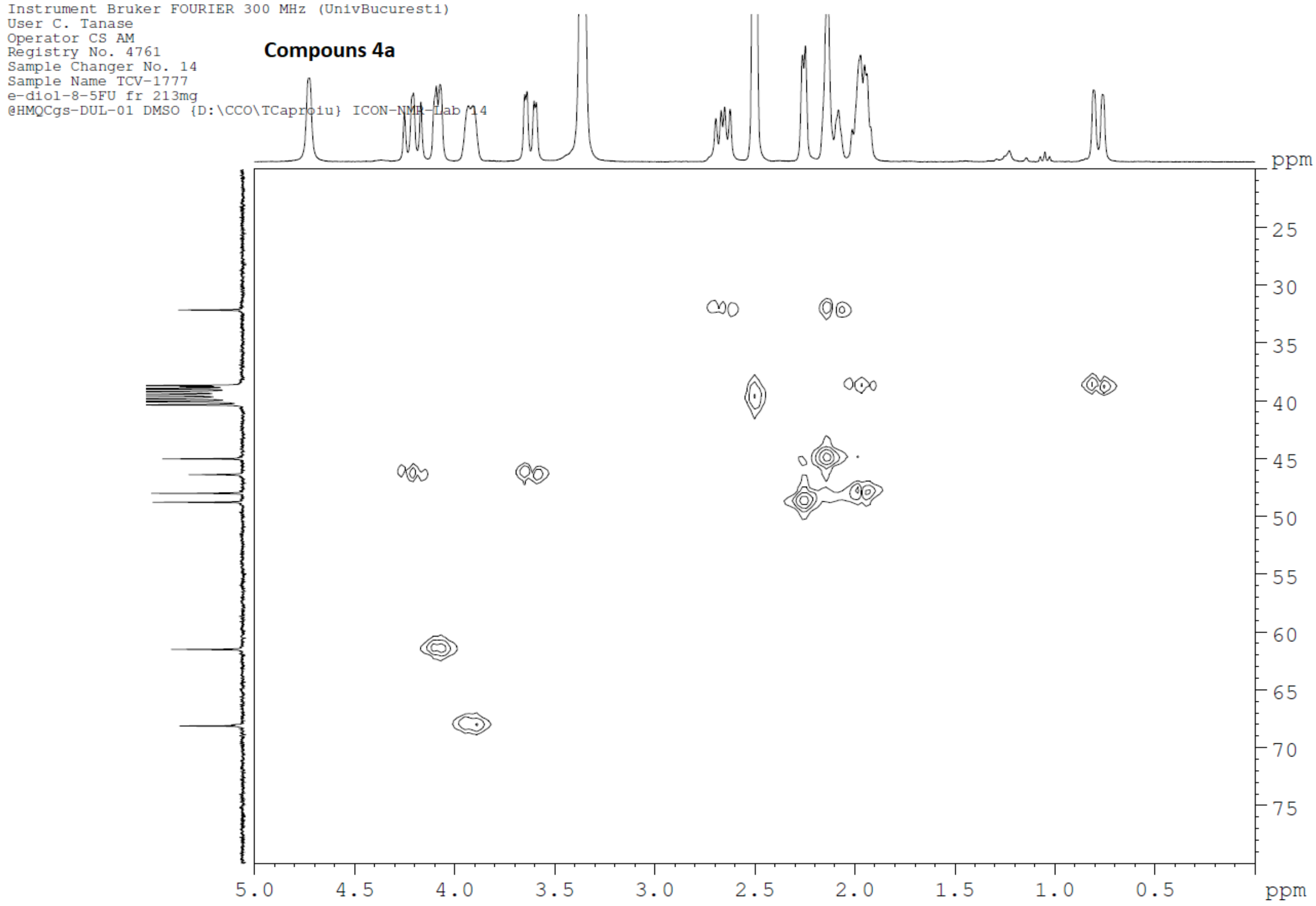
Instrument Bruker FOURIER 300 MHz (UnivBucuresti)
User C. Tanase
Operator CS AM
Registry No. 4761
Sample Changer No. 14
Sample Name TCV-1777
e-diol-8-5FU fr 213mg
@HMQCgs-DUL-01 DMSO {D:\CCO\TCaproiu} ICON-NMR-Lab 14
Compouns 4a
ppm
ppm

1.2. ^{1}H, ^{13}C, COSY and HETCOR (aliphatic)-NMR spectra in DMSO of the compound **4b**

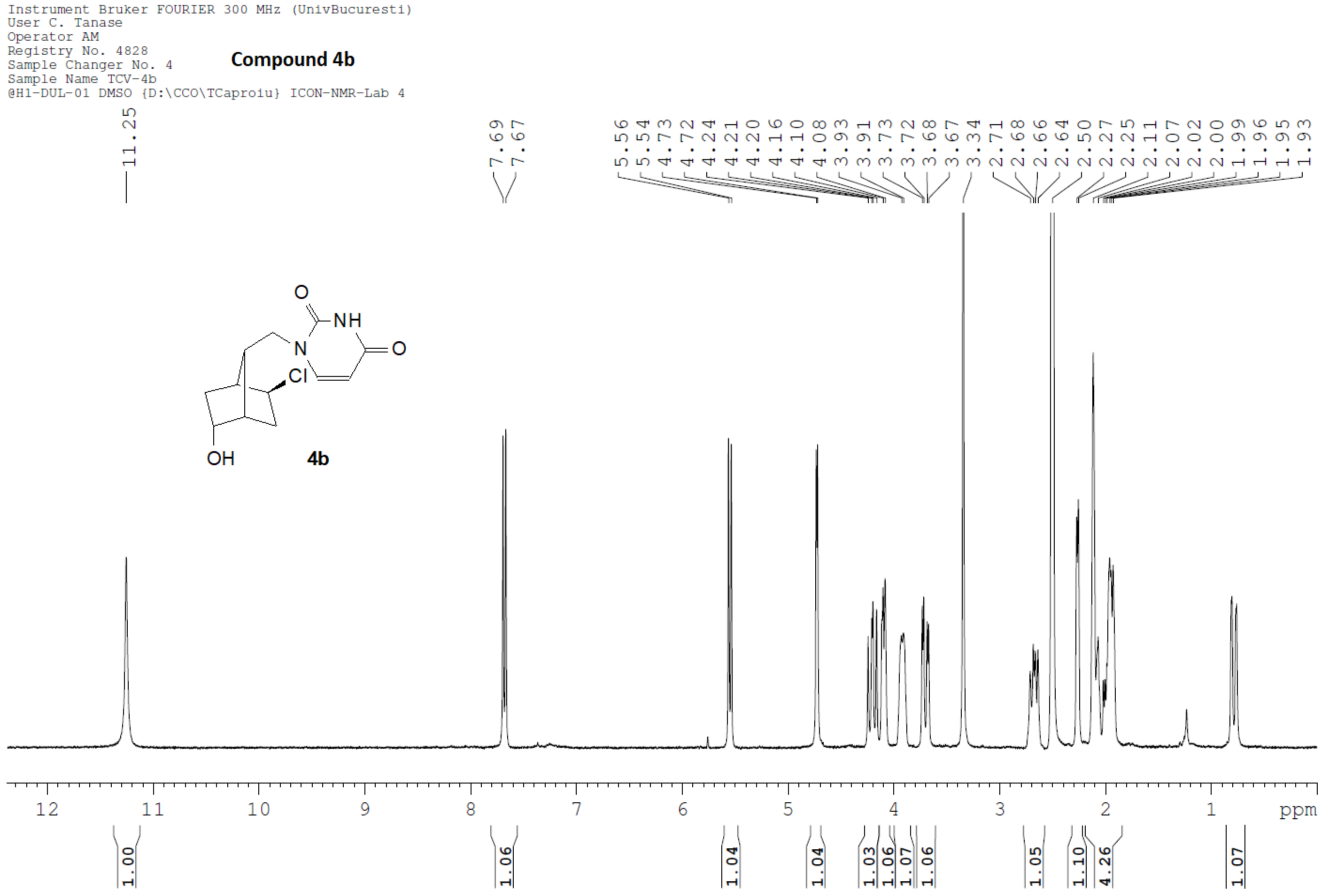

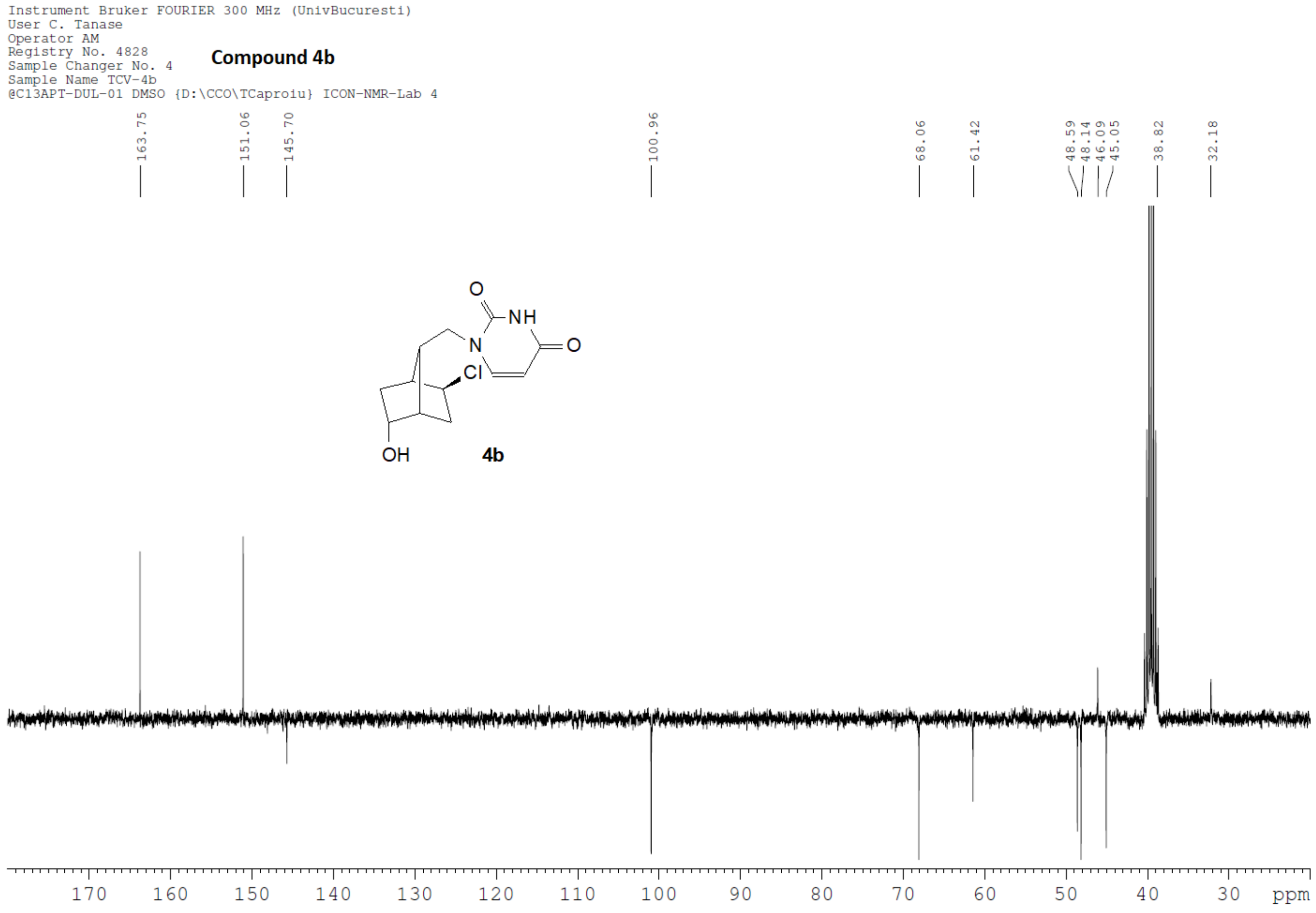

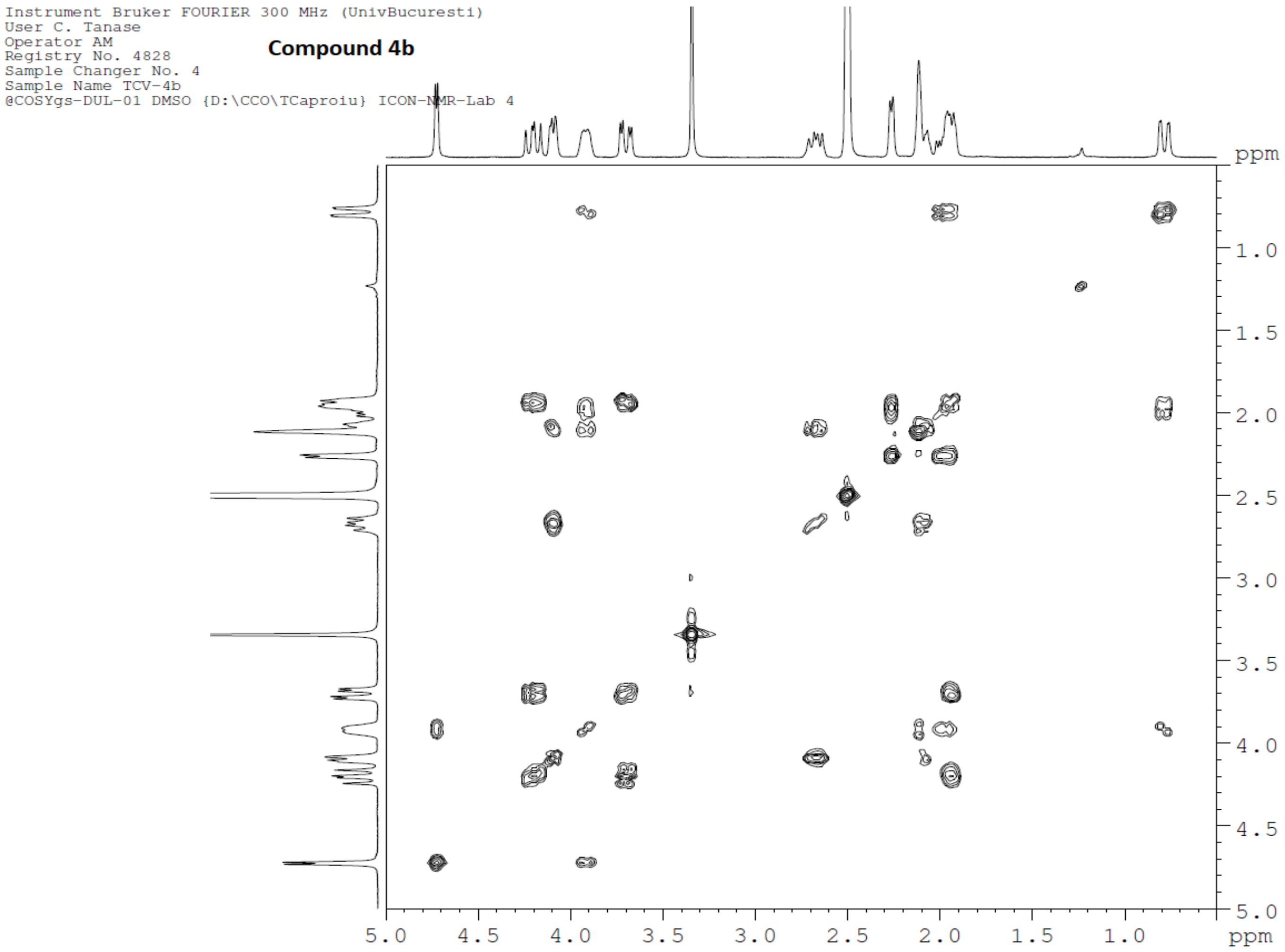
Instrument Bruker FOURIER 300 MHz (UnivBucuresti)
User C. Tanase
Operator AM
Registry No. 4828
Sample Changer No. 4
Sample Name TCV-4b
@COSYgs-DUL-01 DMSO {D:\CCO\TCaproiu} ICON-NMR-Lab 4
Compound 4b
ppm
1.0
1.5
2.0
2.5
3.0
3.5
4.0
4.5
5.0
5.0
4.5
4.0
3.5
3.0
2.5
2.0
1.5
1.0
ppm

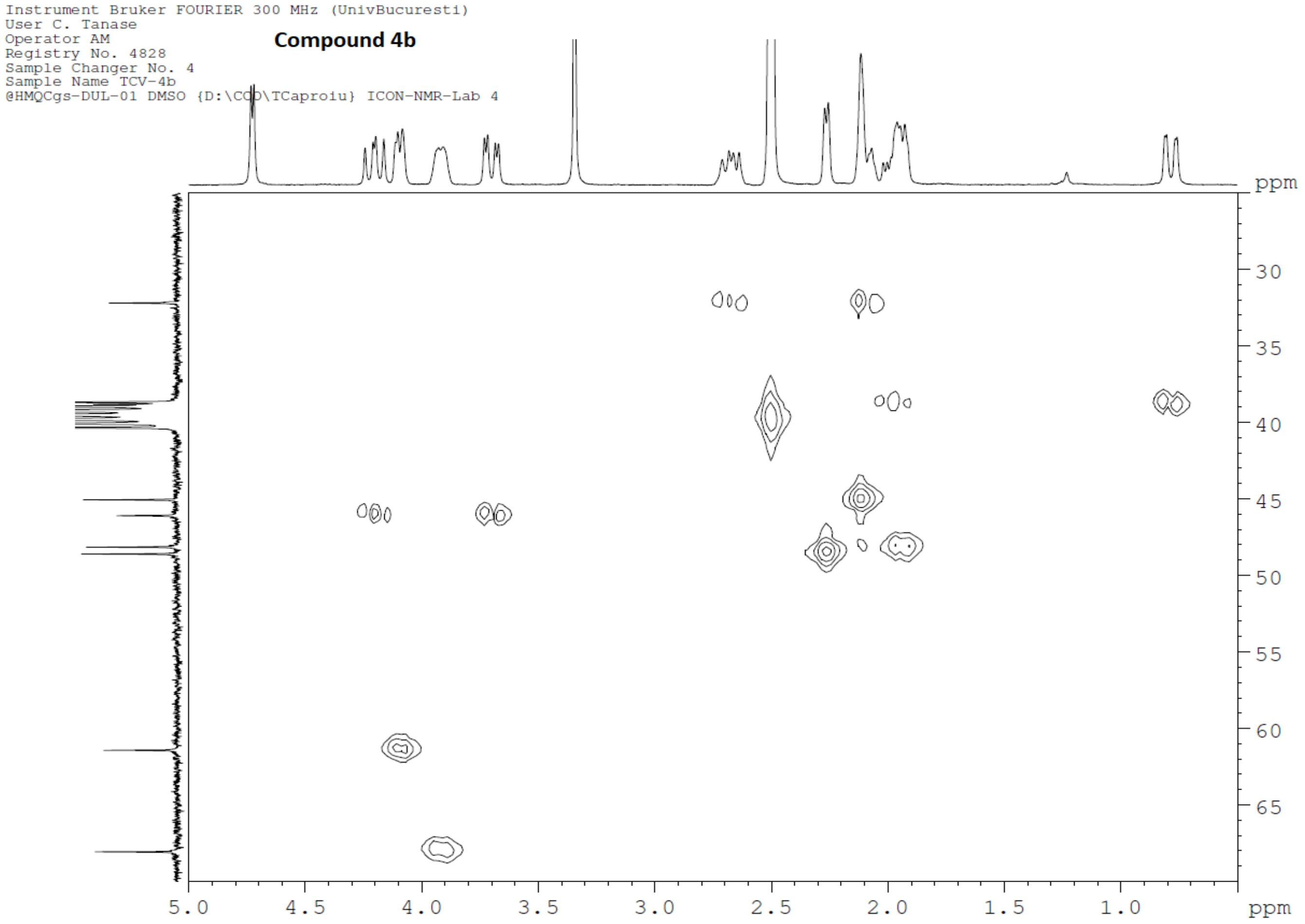
Instrument Bruker FOURIER 300 MHz (UnivBucuresti)
User C. Tanase
Operator AM
Registry No. 4828
Sample Changer No. 4
Sample Name TCV-4b
@HMQCgs-DUL-01 DMSO {D:\CCO\TCaproiu} ICON-NMR-Lab 4
Compound 4b
ppm
30
35
40
45
50
55
60
65
5.0
4.5
4.0
3.5
3.0
2.5
2.0
1.5
1.0
ppm

1.3. ^{1}H, ^{13}C, COSY and HETCOR (aliphatic)-NMR spectra in DMSO of the compound **4c**

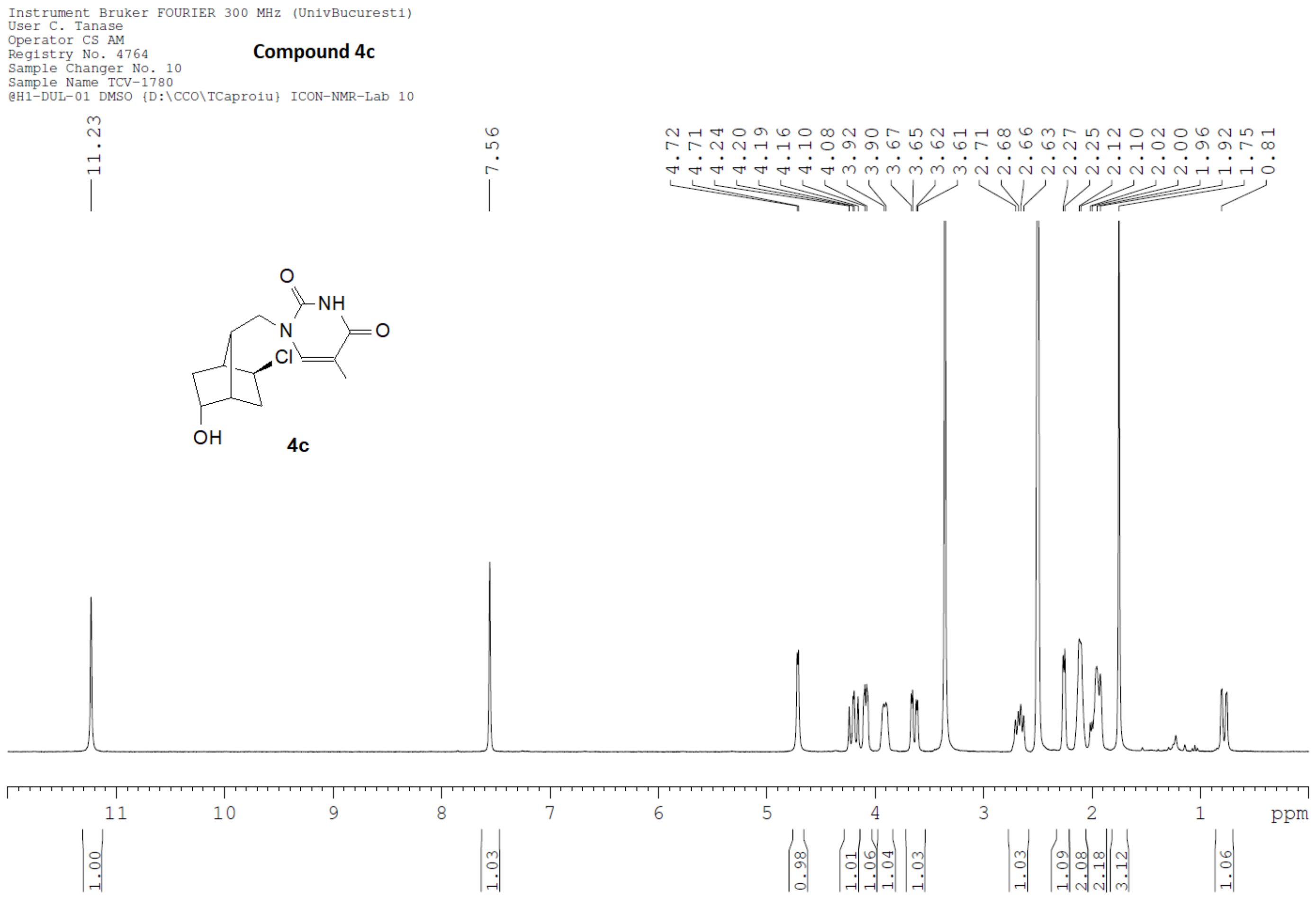

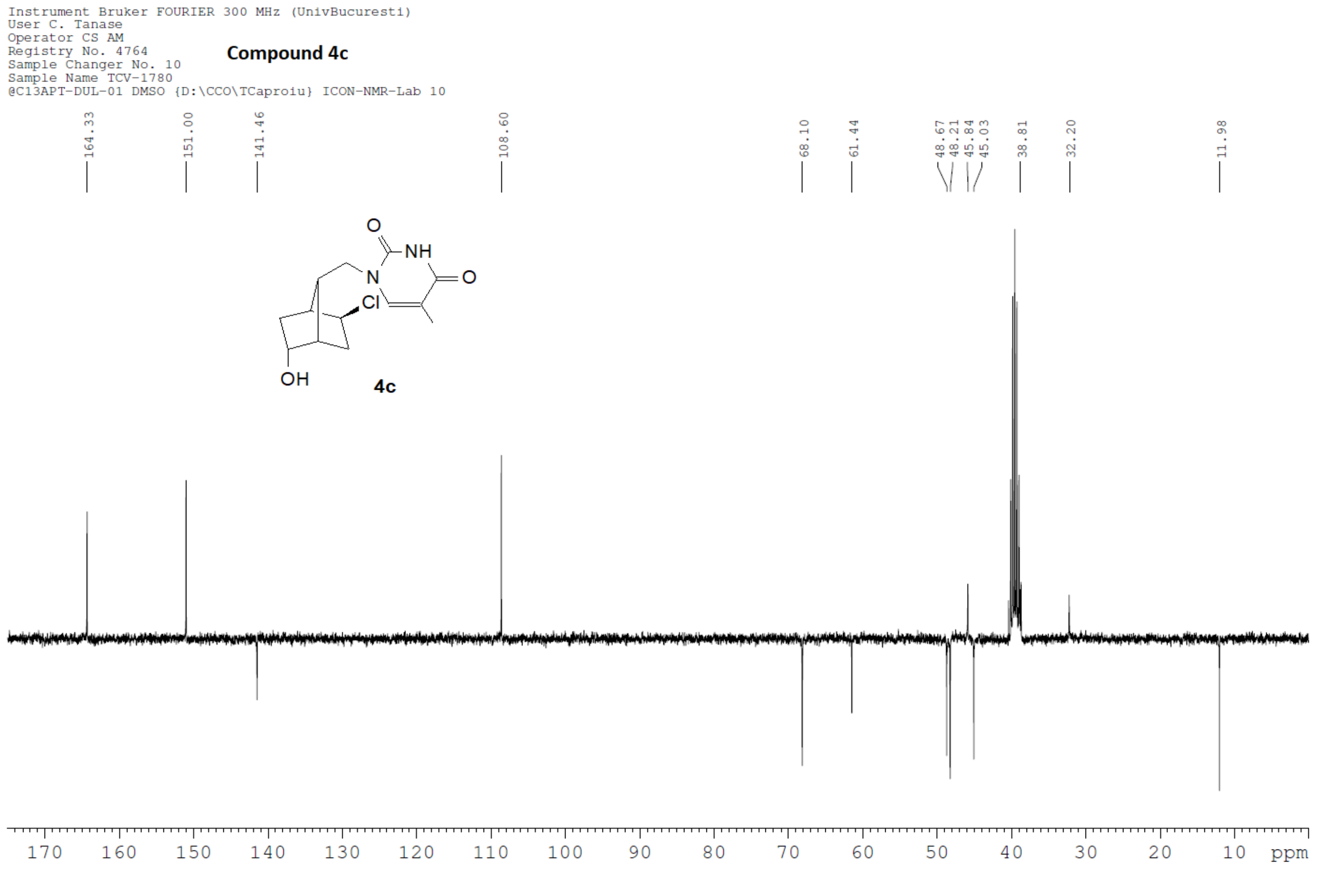

```
Instrument Bruker FOURIER 300 MHz (UnivBucuresti)
User C. Tanase
Operator CS AM
Registry No. 4764
Sample Changer No. 10
Sample Name TCV-1780
@COSYgs-DUL-01 DMSO {D:\CCO\TCaproiu} ICON-NMR-Lab 10
```

Compound 4c

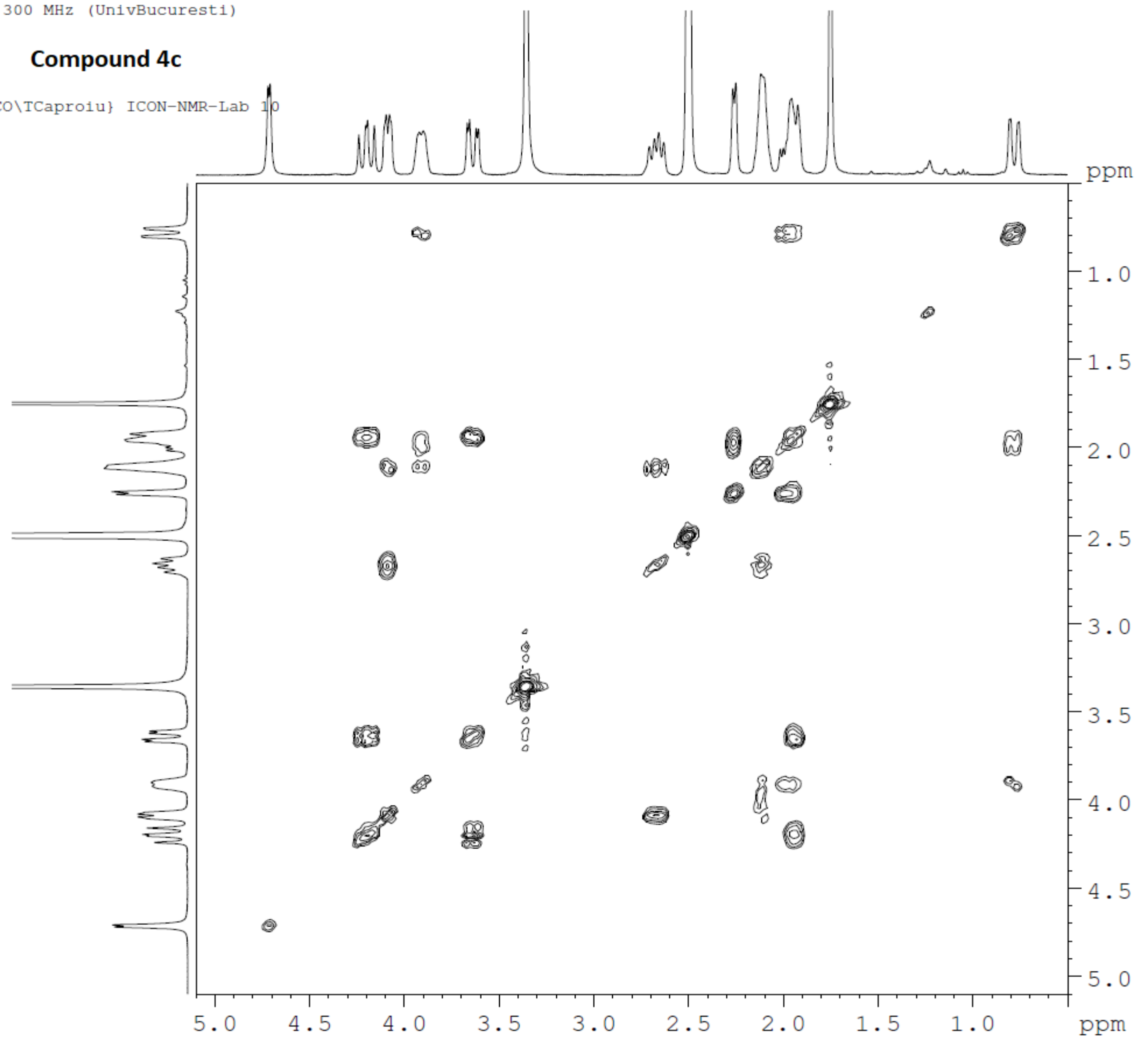

```
Instrument Bruker FOURIER 300 MHz (UnivBucuresti)
User C. Tanase
Operator CS AM
Registry No. 4764
Sample Changer No. 10
Sample Name TCV-1780
@HMQCgs-DUL-01 DMSO {D:\CCO\TCaproiu} ICON-NMR-Lab 10
```

Compound 4c

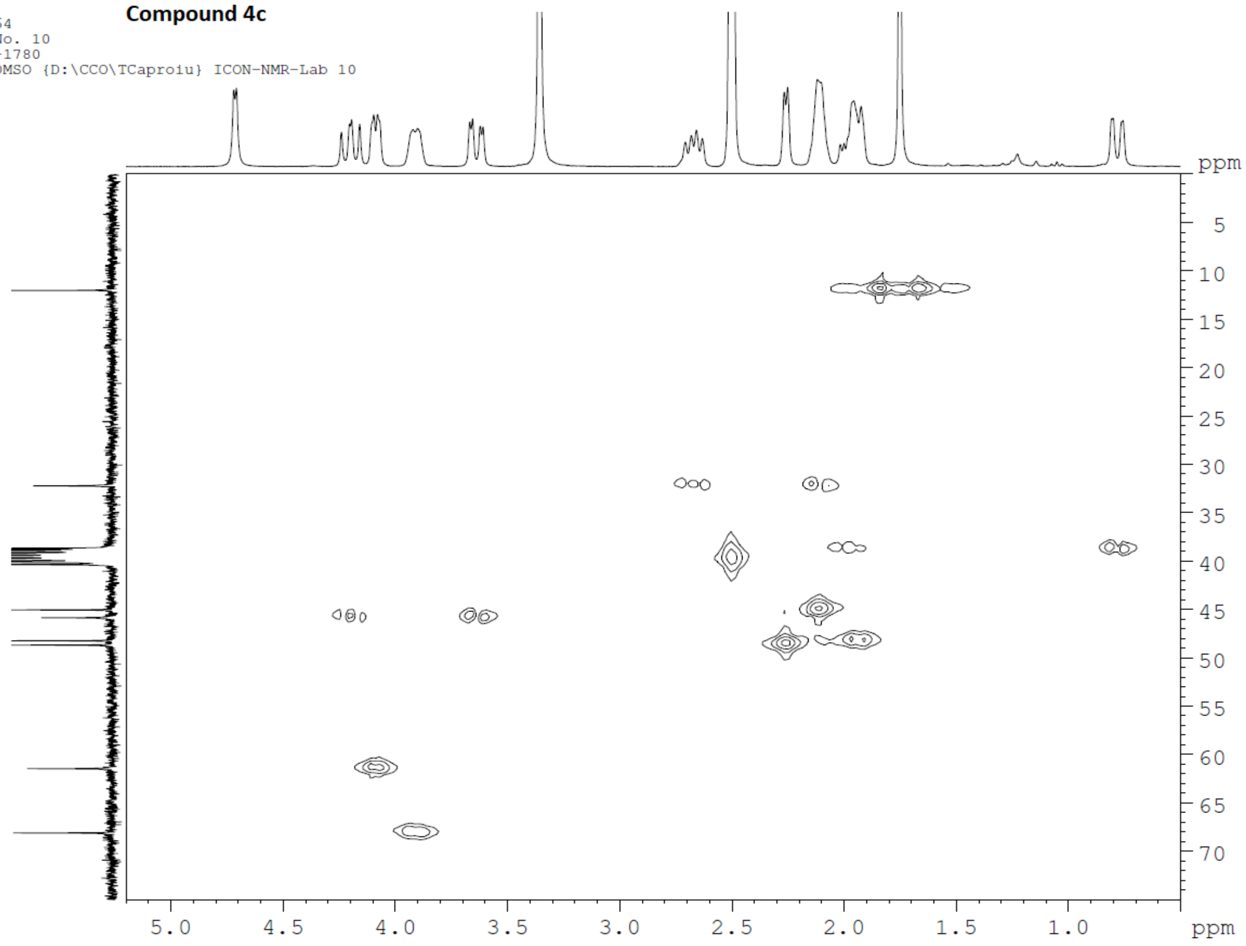

1.4. ^{1}H, ^{13}C, COSY and HETCOR (aliphatic)-NMR spectra in DMSO of the compound **4d**

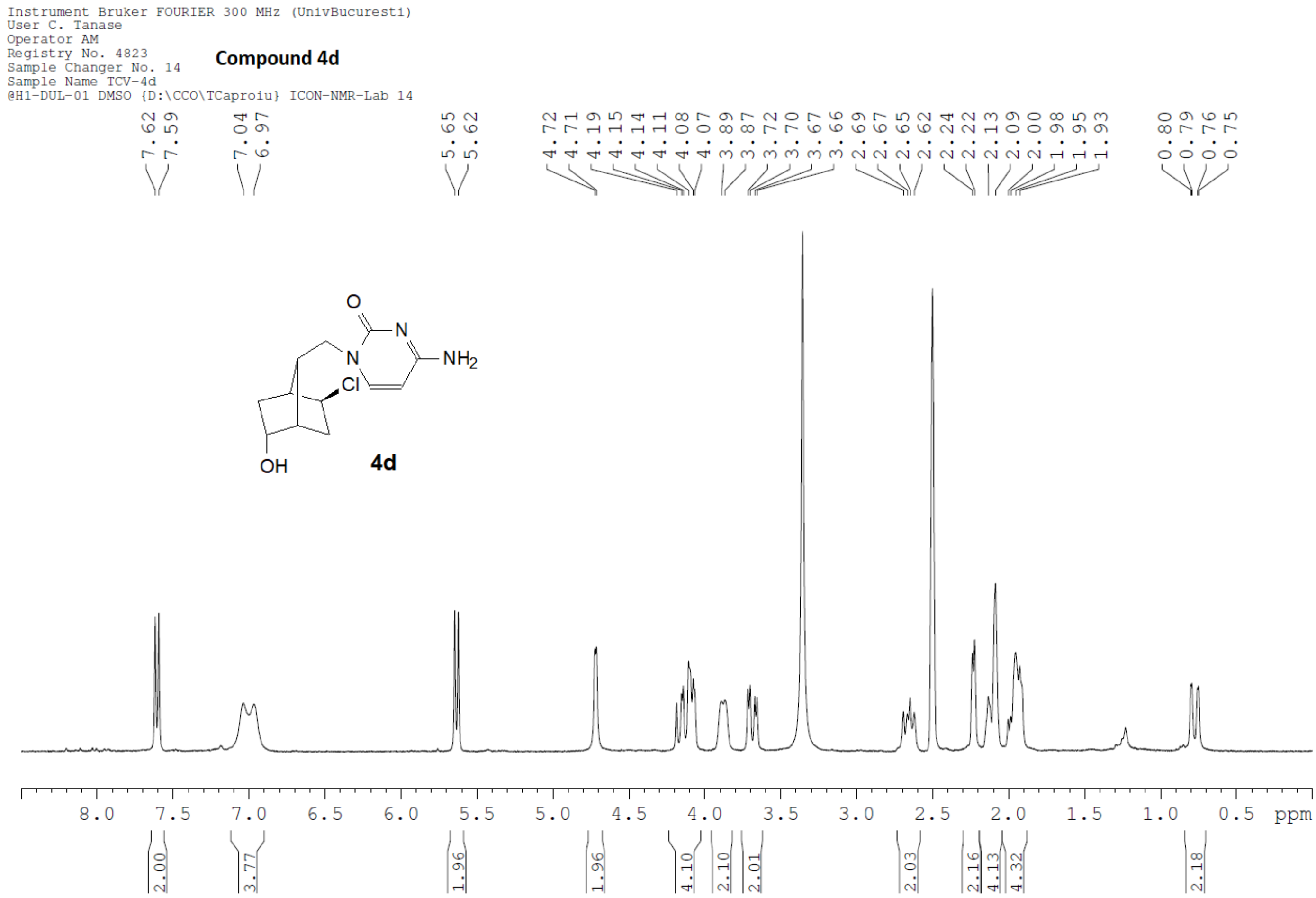

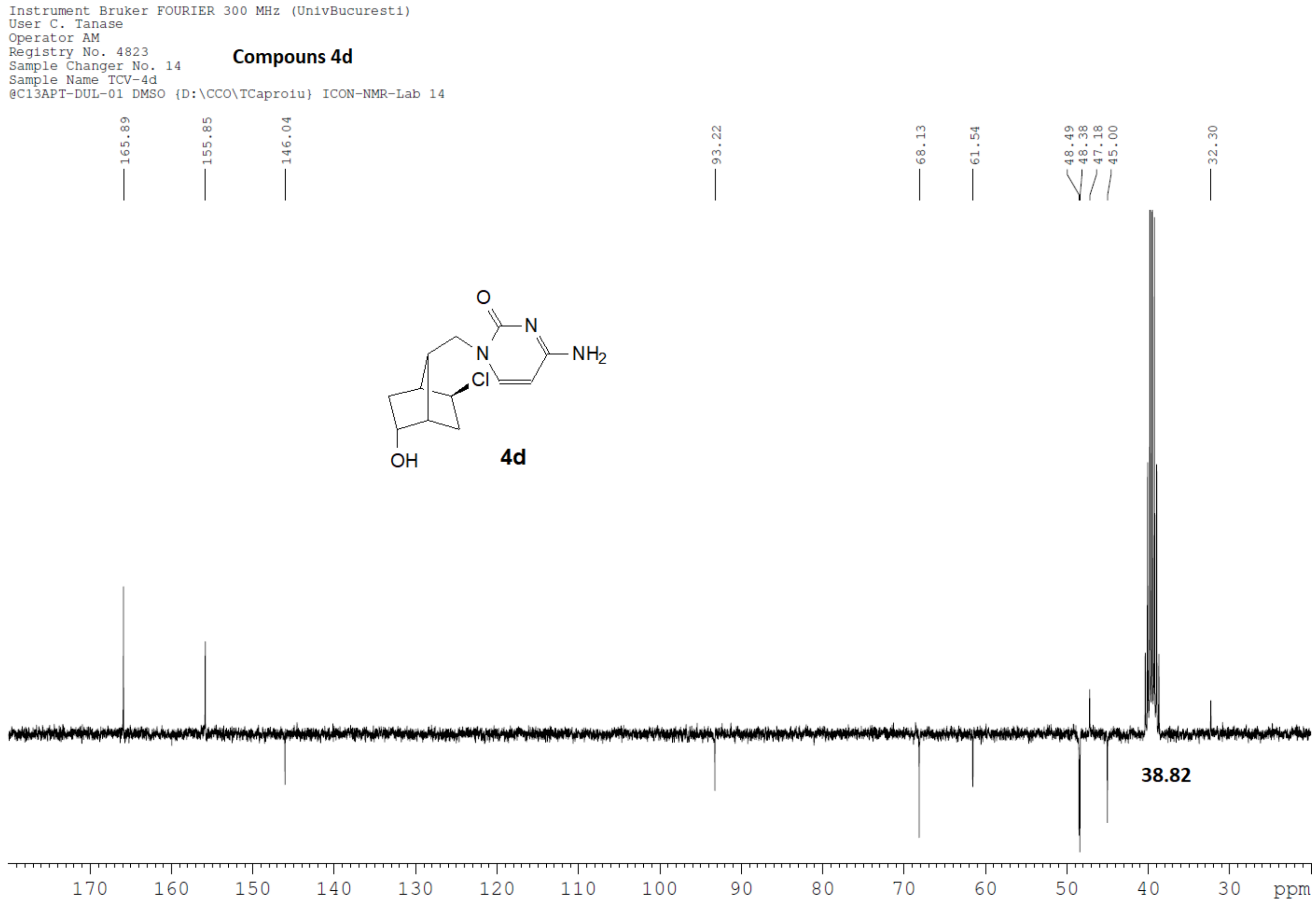

Instrument Bruker FOURIER 300 MHz (UnivBucuresti)
User C. Tanase
Operator AM
Registry No. 4823
Sample Changer No. 14
Sample Name TCV-4d
@COSYgs-DUL-01 DMSO {D:\CCO\TCaproiu} ICON-NMR-Lab 14

Compound 4d

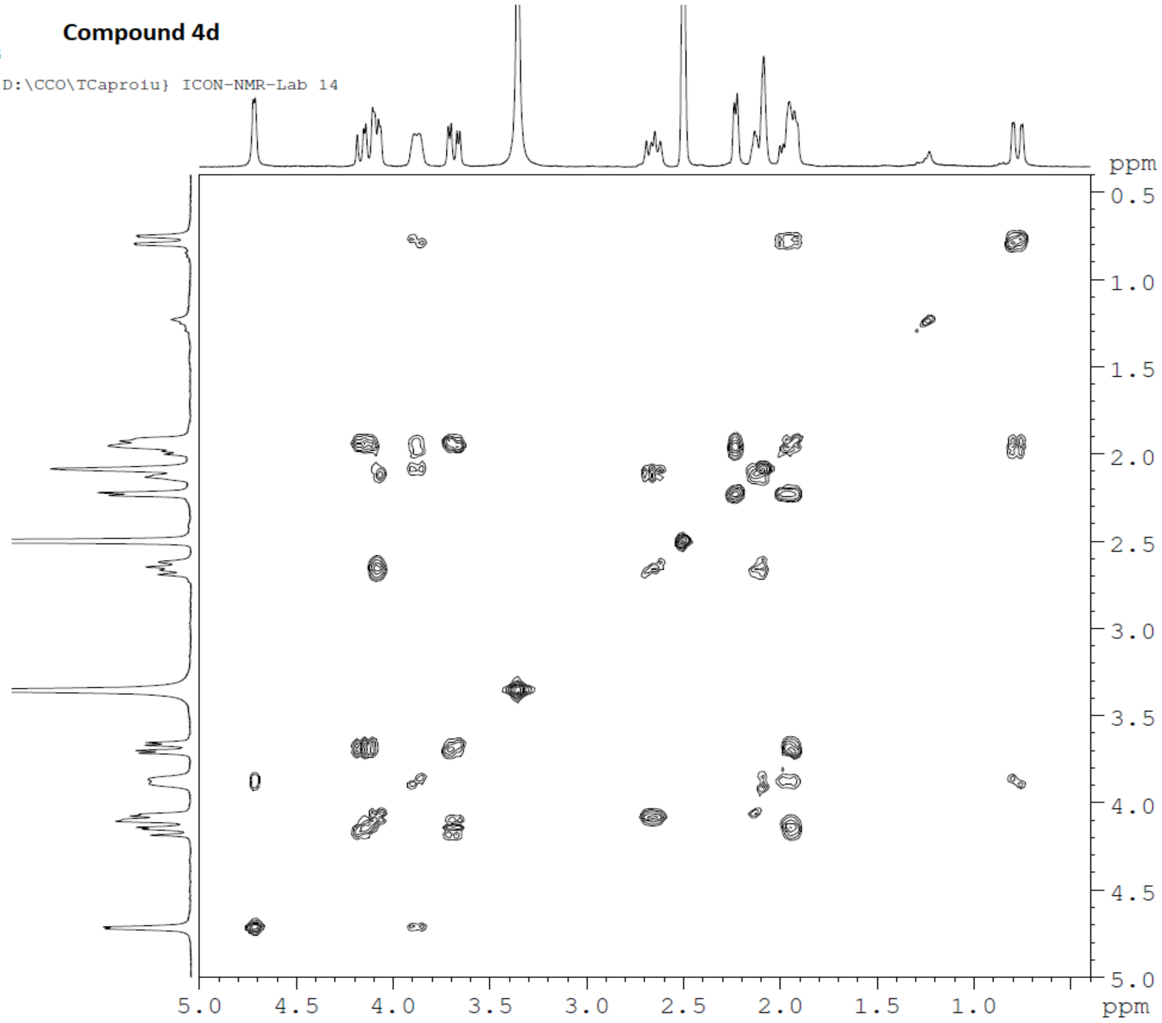

Instrument Bruker FOURIER 300 MHz (UnivBucuresti)
User C. Tanase
Operator AM
Registry No. 4823
Sample Changer No. 14
Sample Name TCV-4d
@HMQCgs-DUL-01 DMSO {D:\CCO\TCaproiu} ICON-NMR-Lab 14

ompound 4d

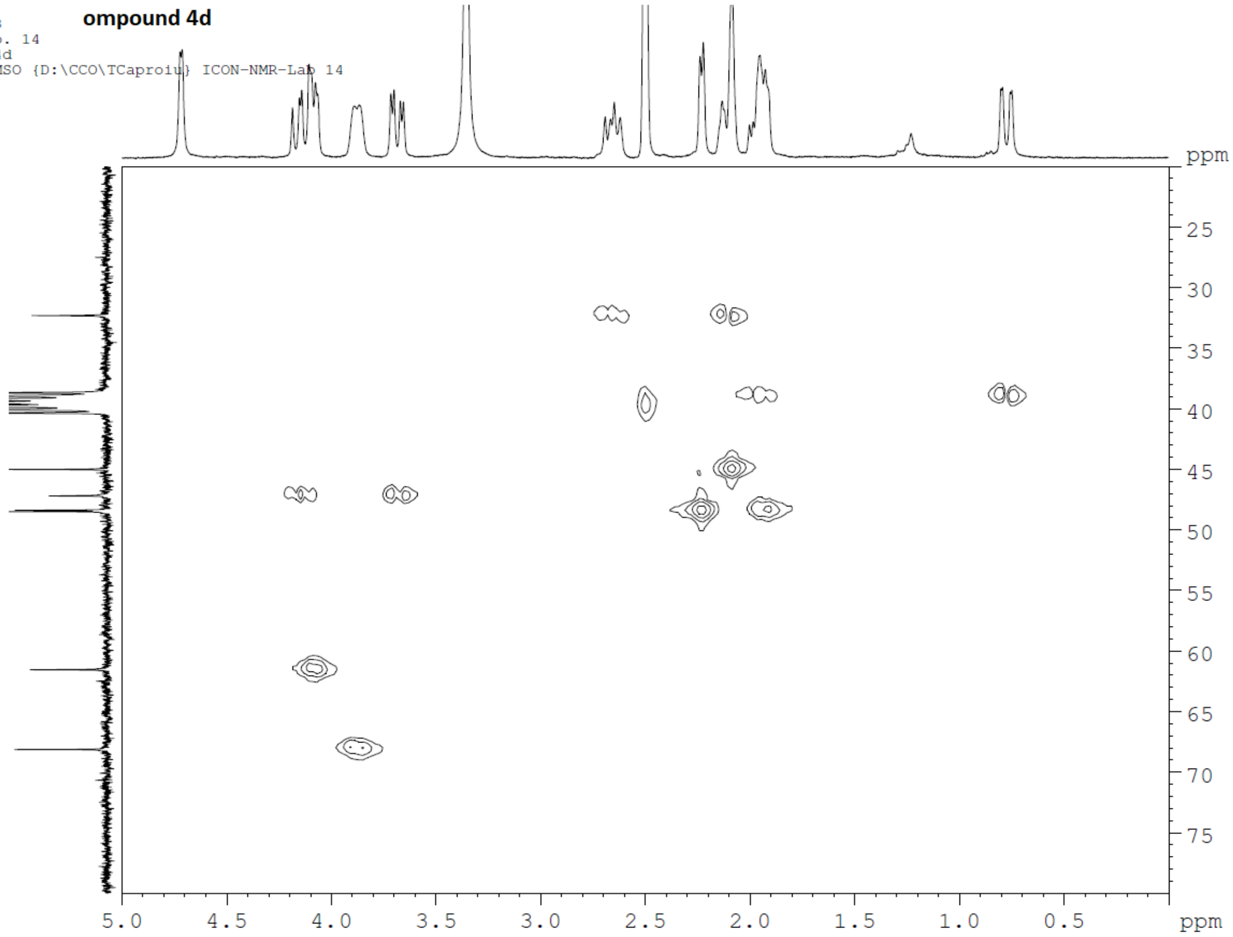

1.5. ^{1}H, ^{13}C, COSY and HETCOR (aliphatic)-NMR spectra in DMSO of the compound **5**

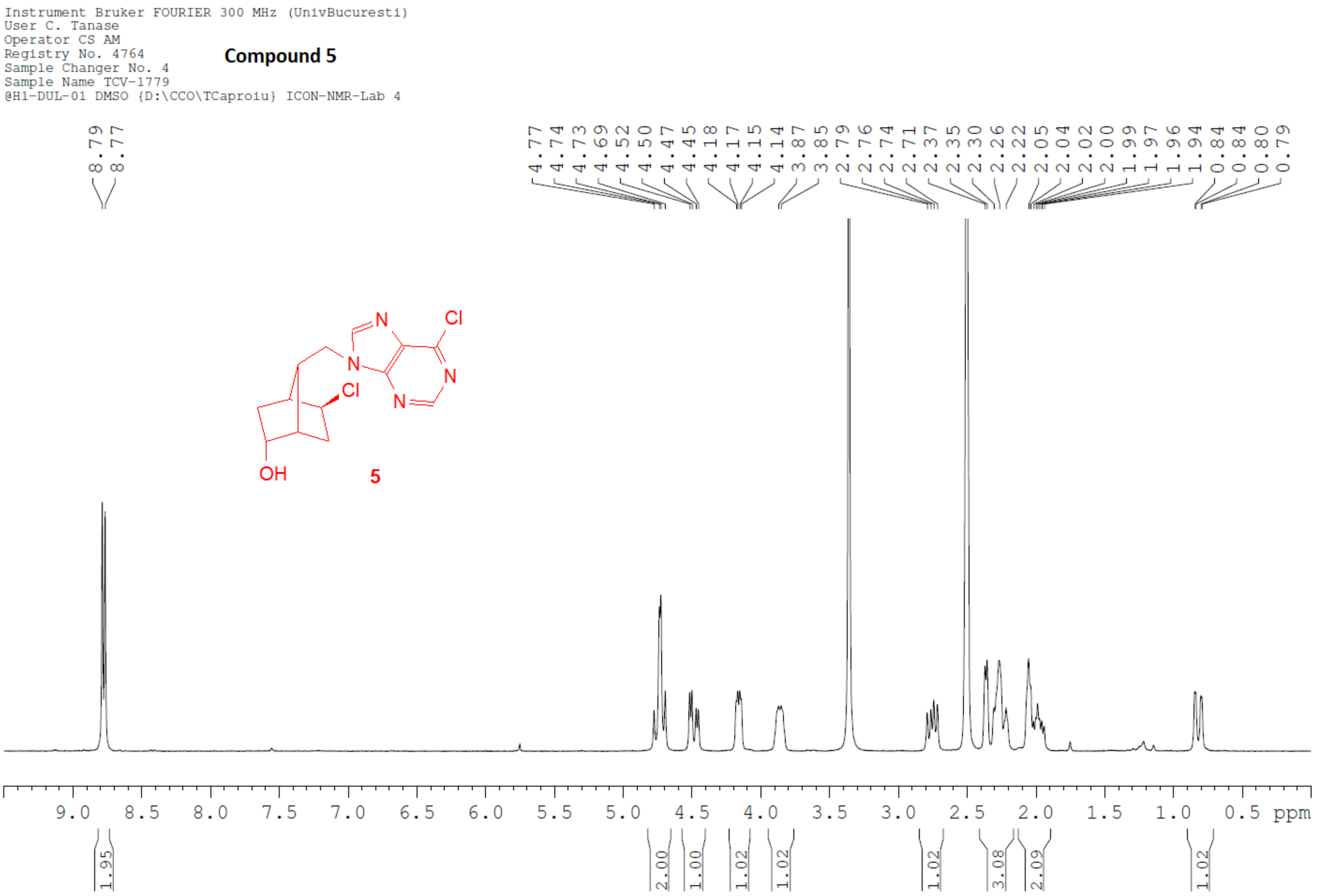

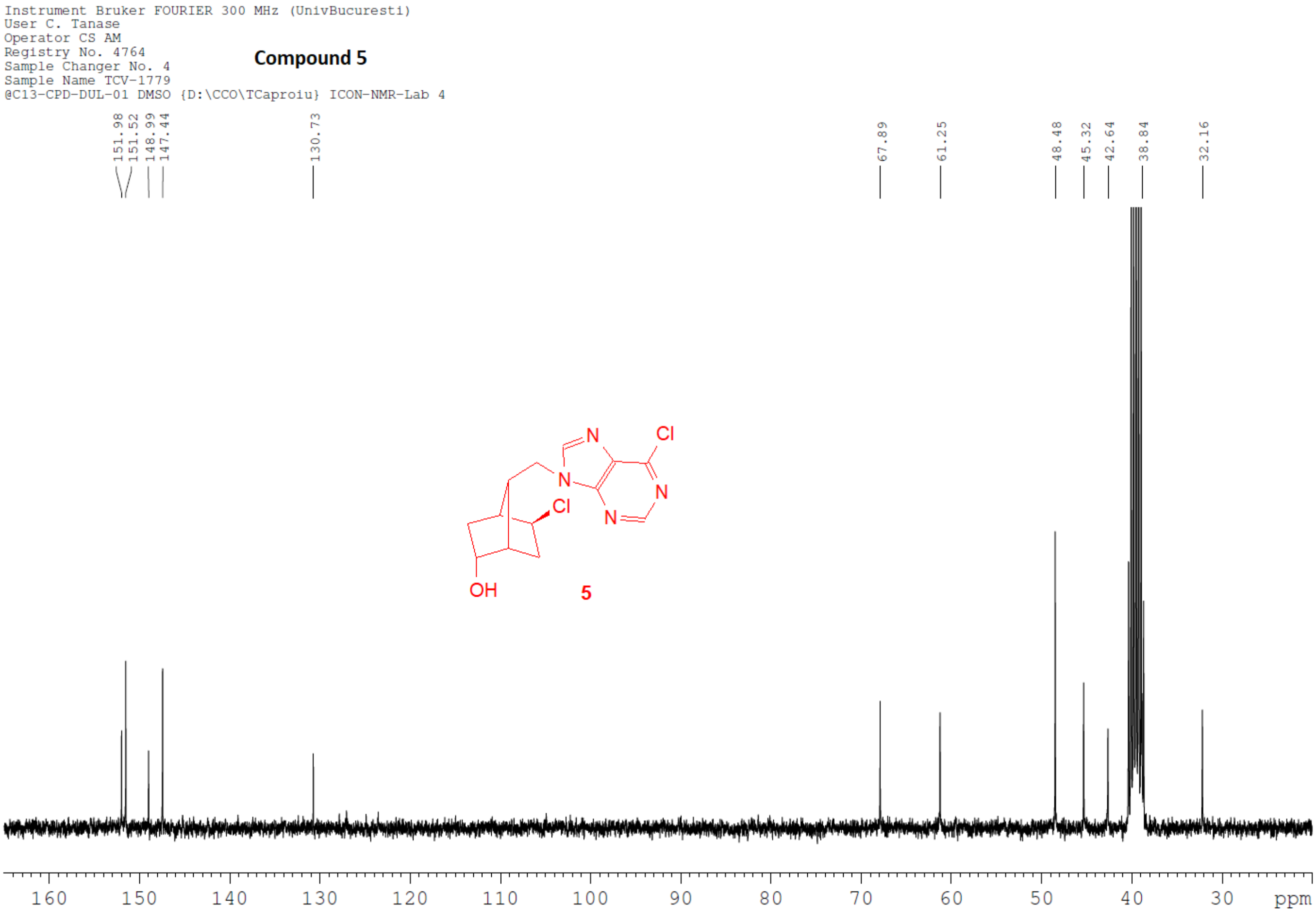

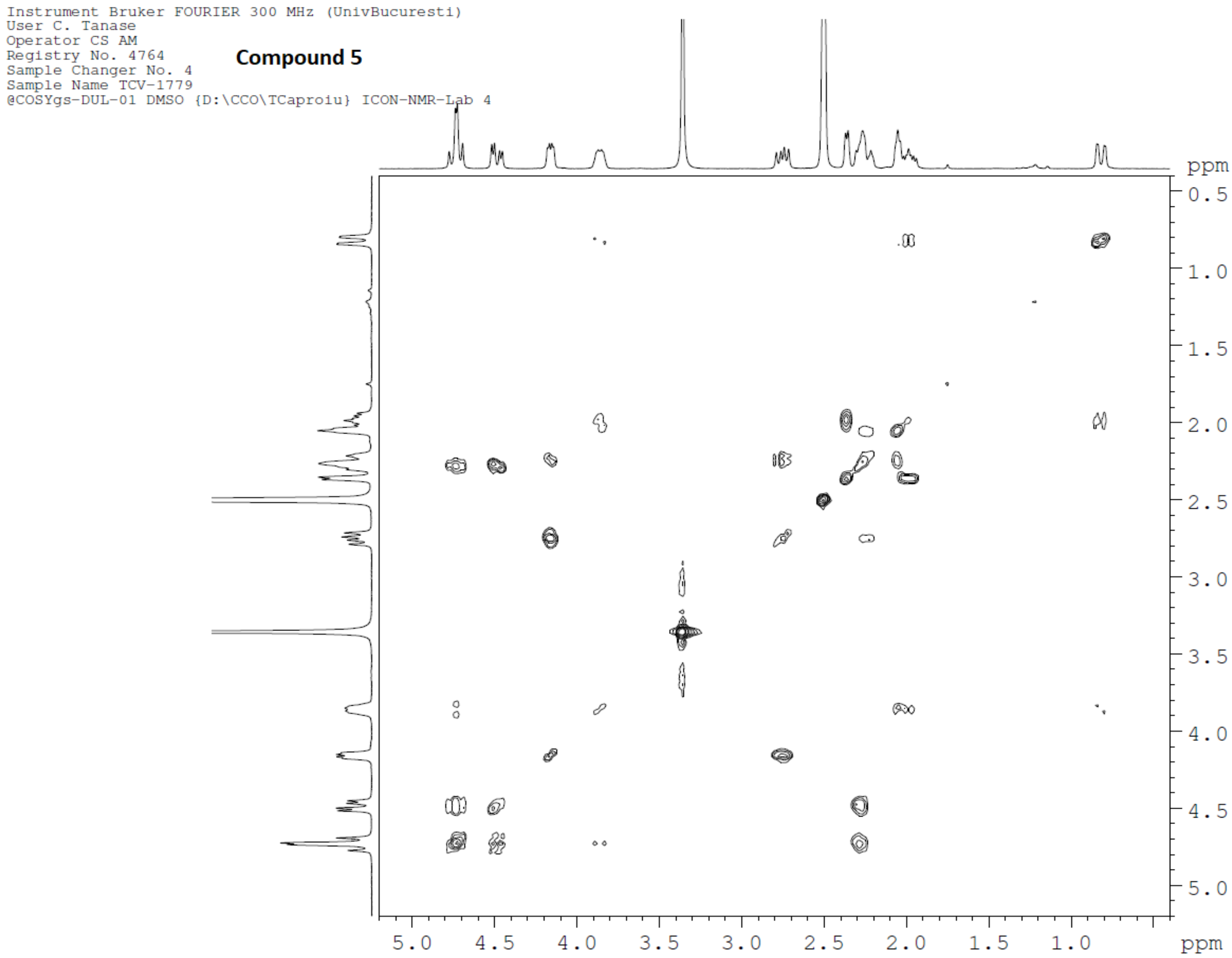
Instrument Bruker FOURIER 300 MHz (UnivBucuresti)
User C. Tanase
Operator CS AM
Registry No. 4764
Sample Changer No. 4
Sample Name TCV-1779
@COSYgs-DUL-01 DMSO {D:\CCO\TCaproiu} ICON-NMR-Lab 4
Compound 5
ppm
0.5
1.0
1.5
2.0
2.5
3.0
3.5
4.0
4.5
5.0
5.0
4.5
4.0
3.5
3.0
2.5
2.0
1.5
1.0
ppm

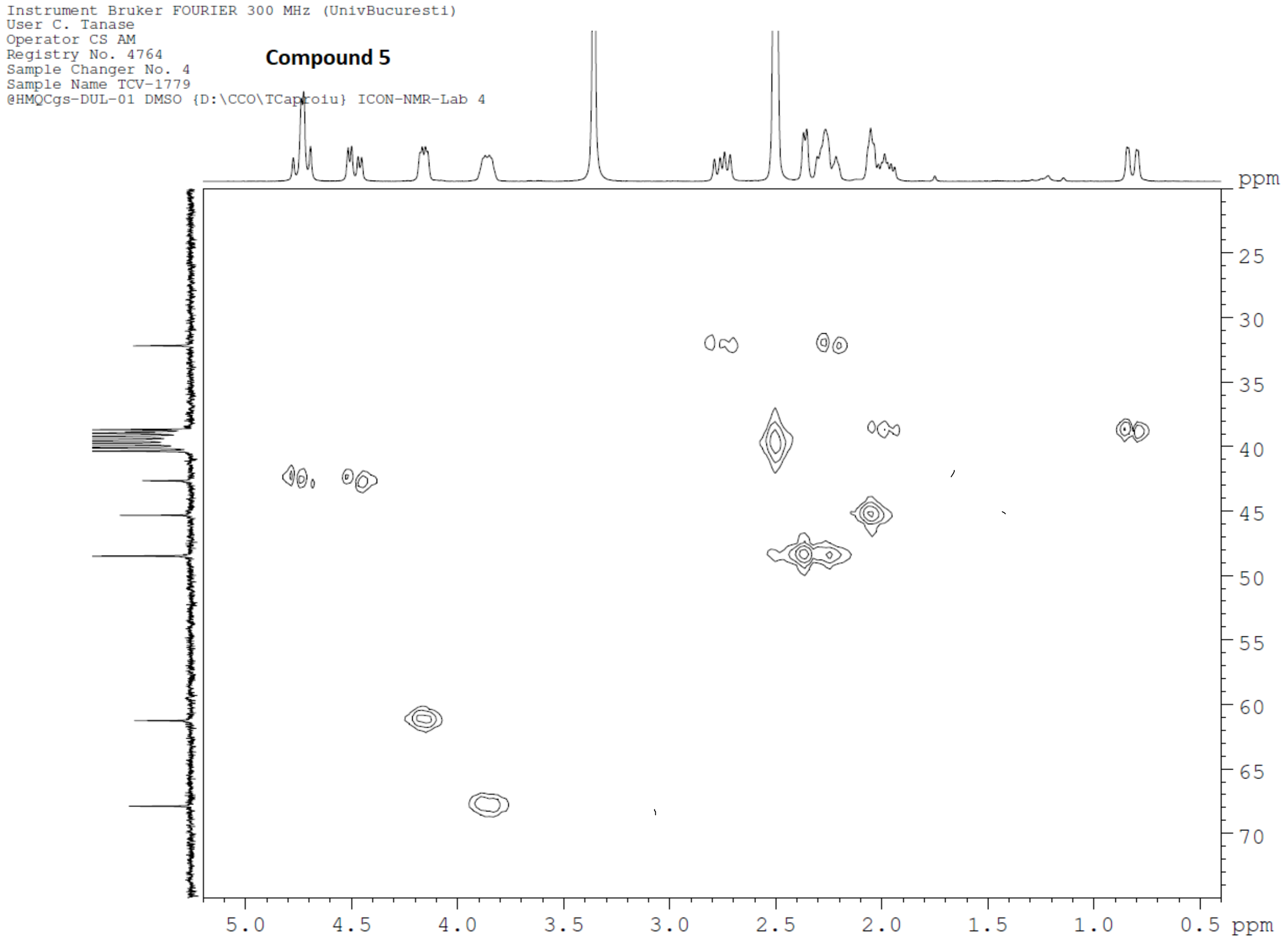
Instrument Bruker FOURIER 300 MHz (UnivBucuresti)
User C. Tanase
Operator CS AM
Registry No. 4764
Sample Changer No. 4
Sample Name TCV-1779
@HMQCgs-DUL-01 DMSO {D:\CCO\TCaproiu} ICON-NMR-Lab 4
Compound 5
ppm
25
30
35
40
45
50
55
60
65
70
5.0
4.5
4.0
3.5
3.0
2.5
2.0
1.5
1.0
0.5
ppm

1.6. ^{1}H, ^{13}C, COSY and HETCOR (aliphatic)-NMR spectra in DMSO of the compound **6a**

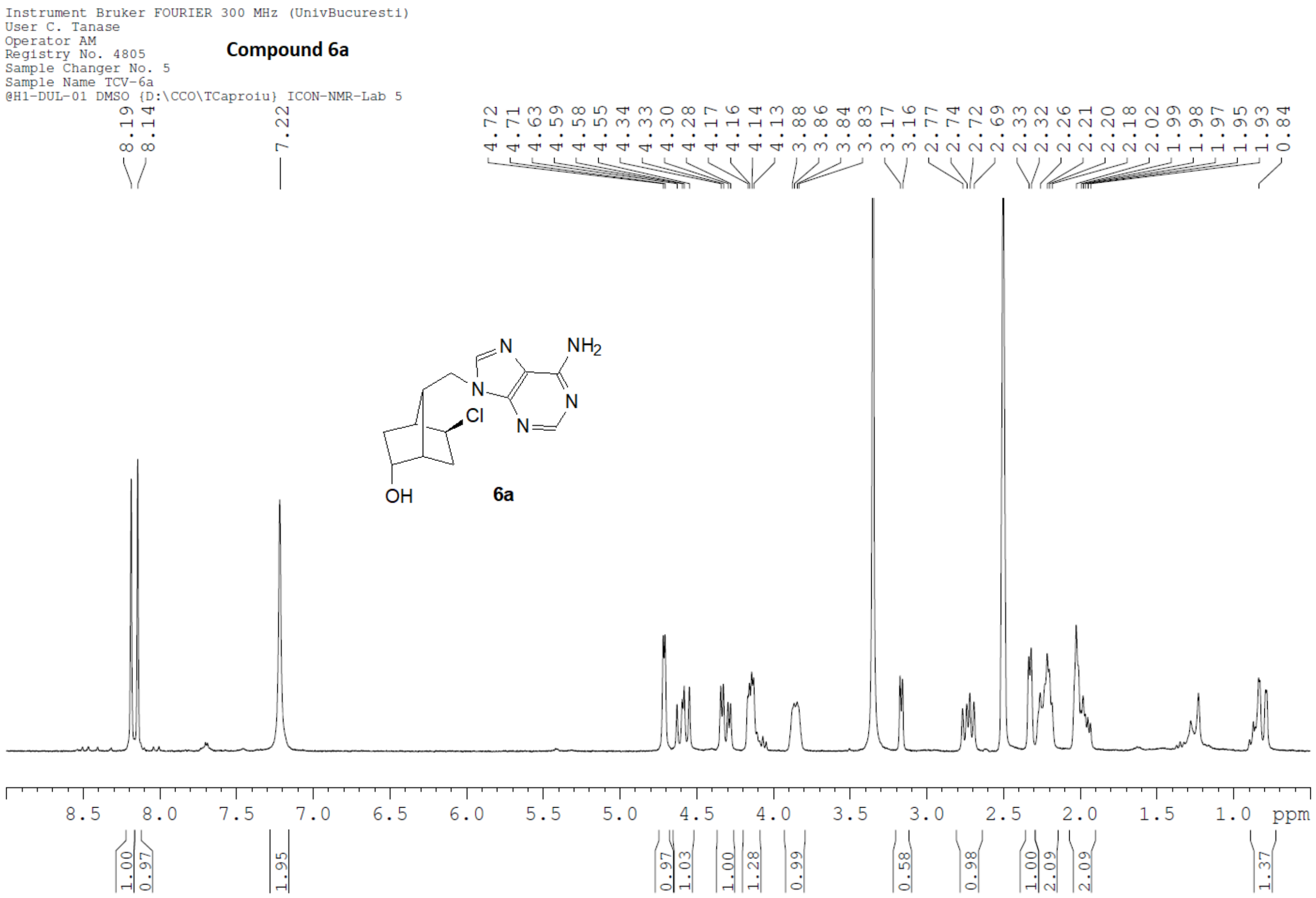

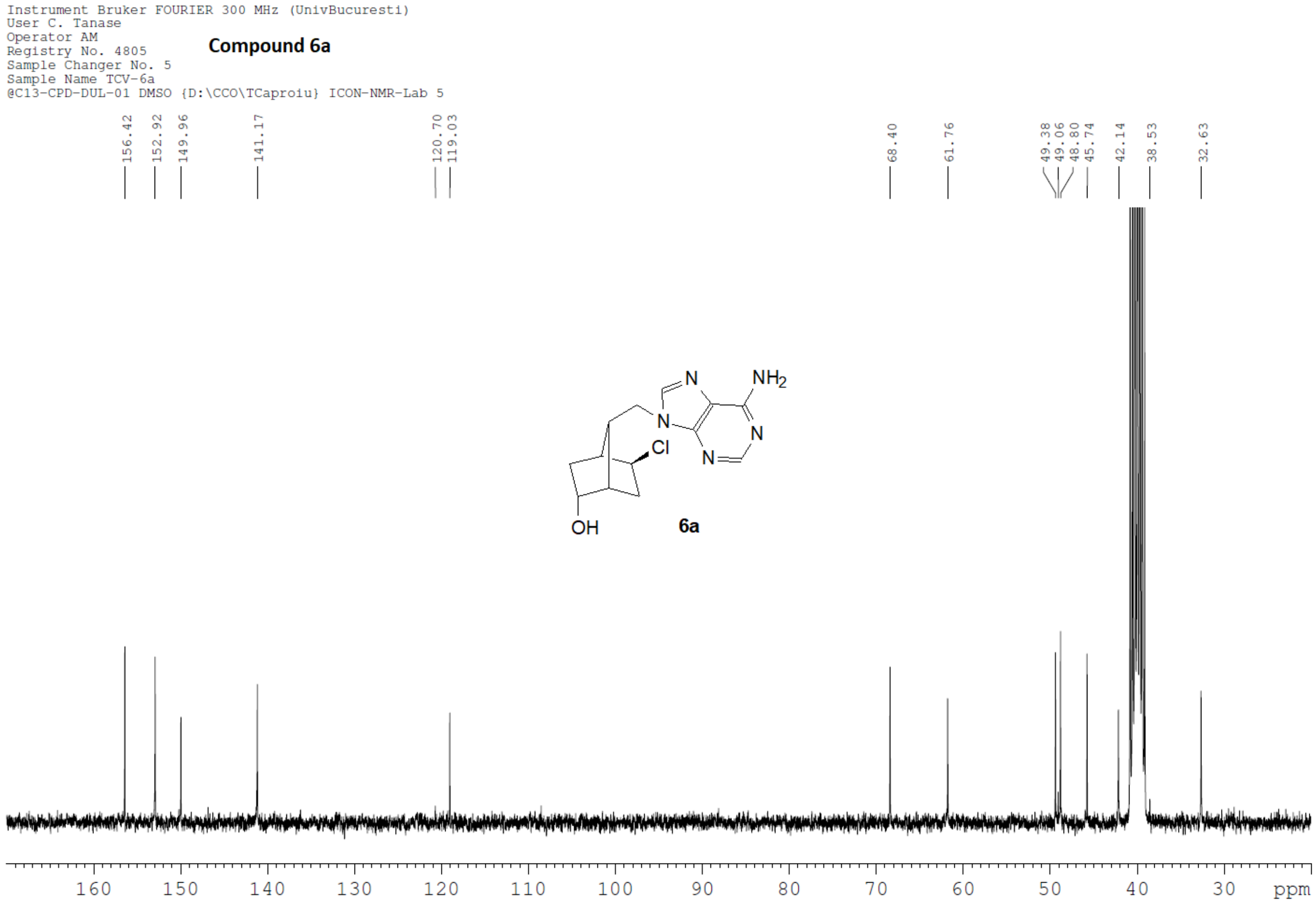

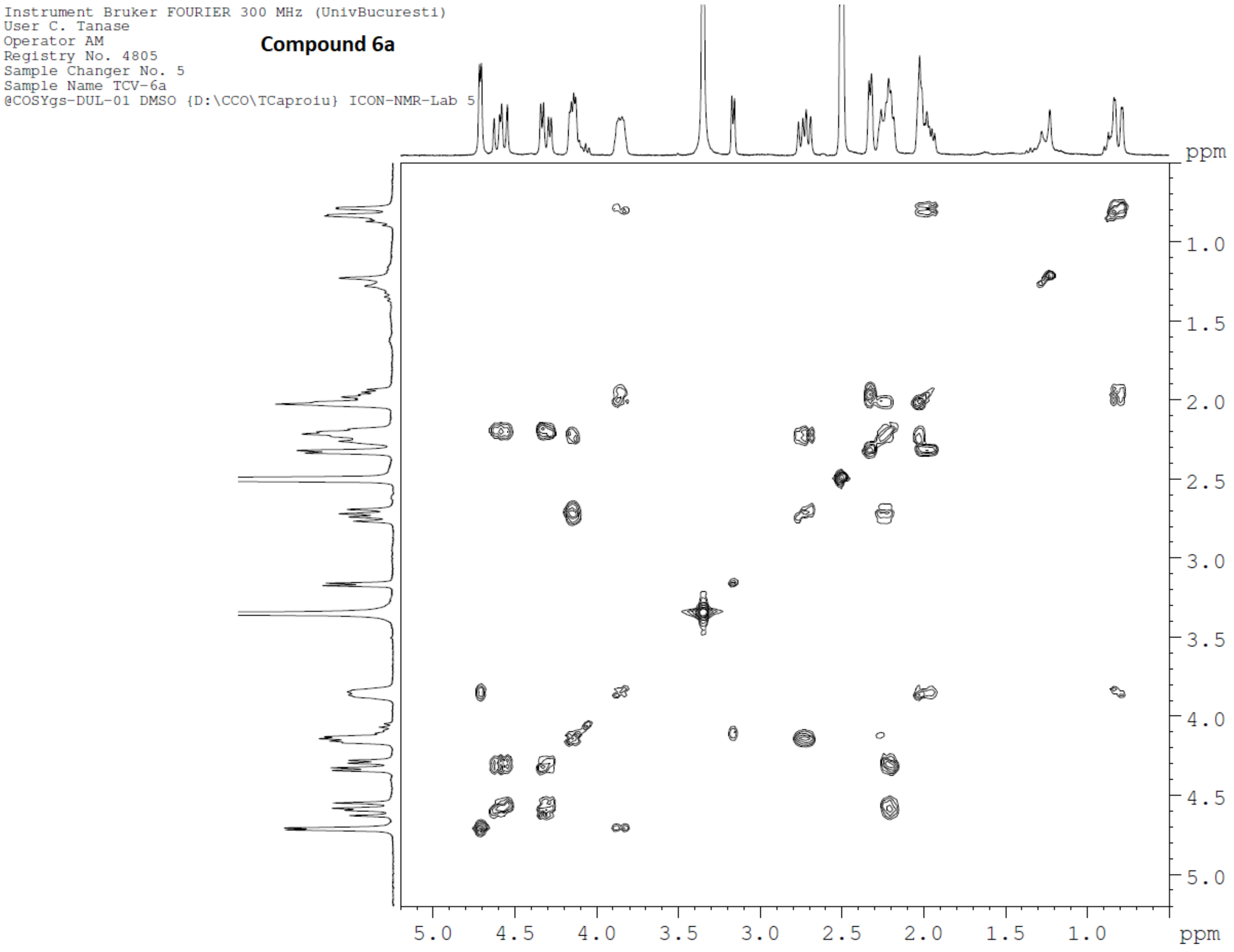
Instrument Bruker FOURIER 300 MHz (UnivBucuresti)
User C. Tanase
Operator AM
Registry No. 4805
Sample Changer No. 5
Sample Name TCV-6a
@COSYgs-DUL-01 DMSO {D:\CCO\TCaproiu} ICON-NMR-Lab 5
Compound 6a
ppm
1.0
1.5
2.0
2.5
3.0
3.5
4.0
4.5
5.0
5.0
4.5
4.0
3.5
3.0
2.5
2.0
1.5
1.0
ppm

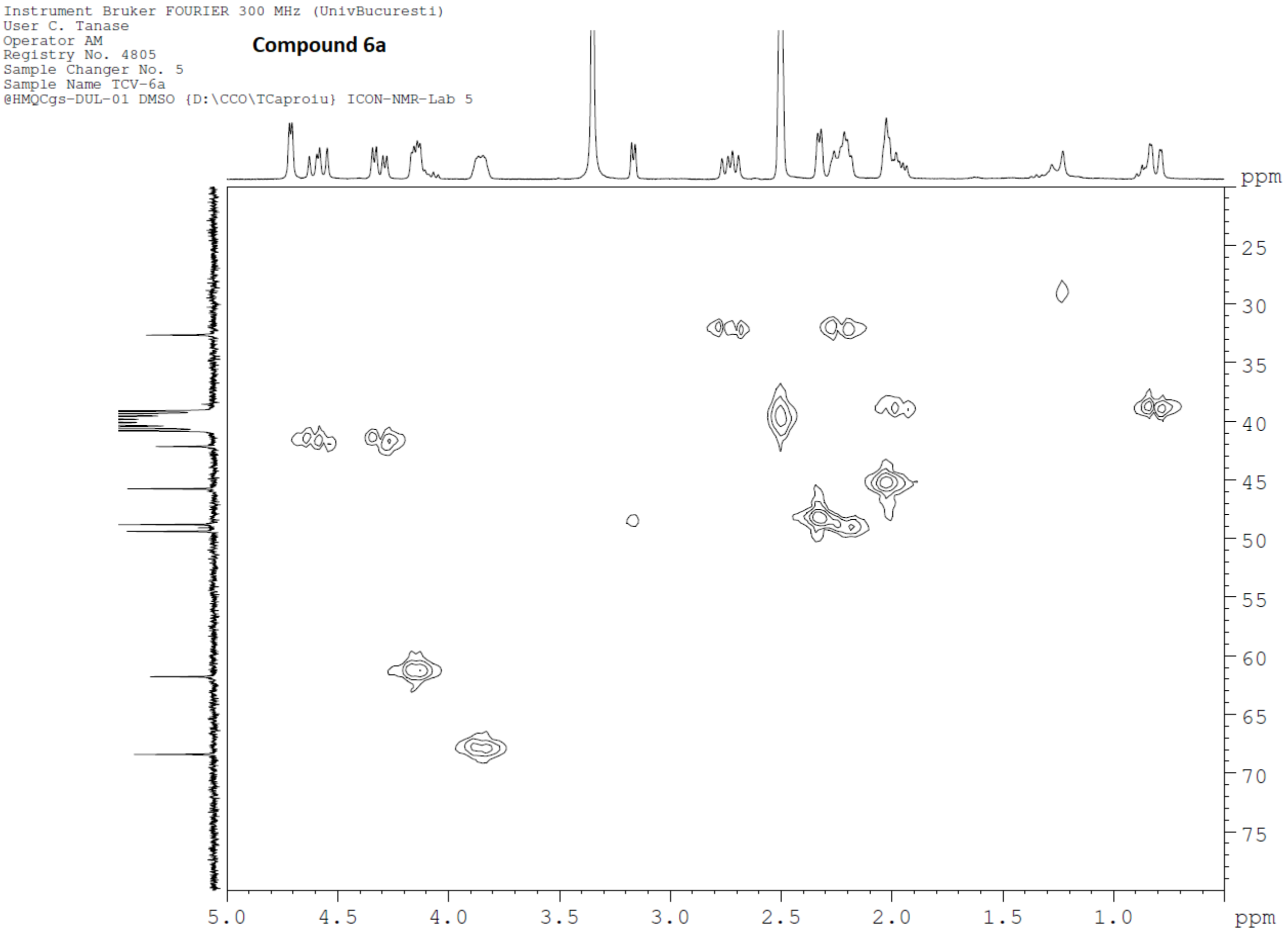
Instrument Bruker FOURIER 300 MHz (UnivBucuresti)
User C. Tanase
Operator AM
Registry No. 4805
Sample Changer No. 5
Sample Name TCV-6a
@HMQCgs-DUL-01 DMSO {D:\CCO\TCaproiu} ICON-NMR-Lab 5
Compound 6a
ppm
25
30
35
40
45
50
55
60
65
70
75
5.0
4.5
4.0
3.5
3.0
2.5
2.0
1.5
1.0
ppm

1.7. ^{1}H, ^{13}C, COSY and HETCOR (aliphatic)-NMR spectra in DMSO of the compound **6b**

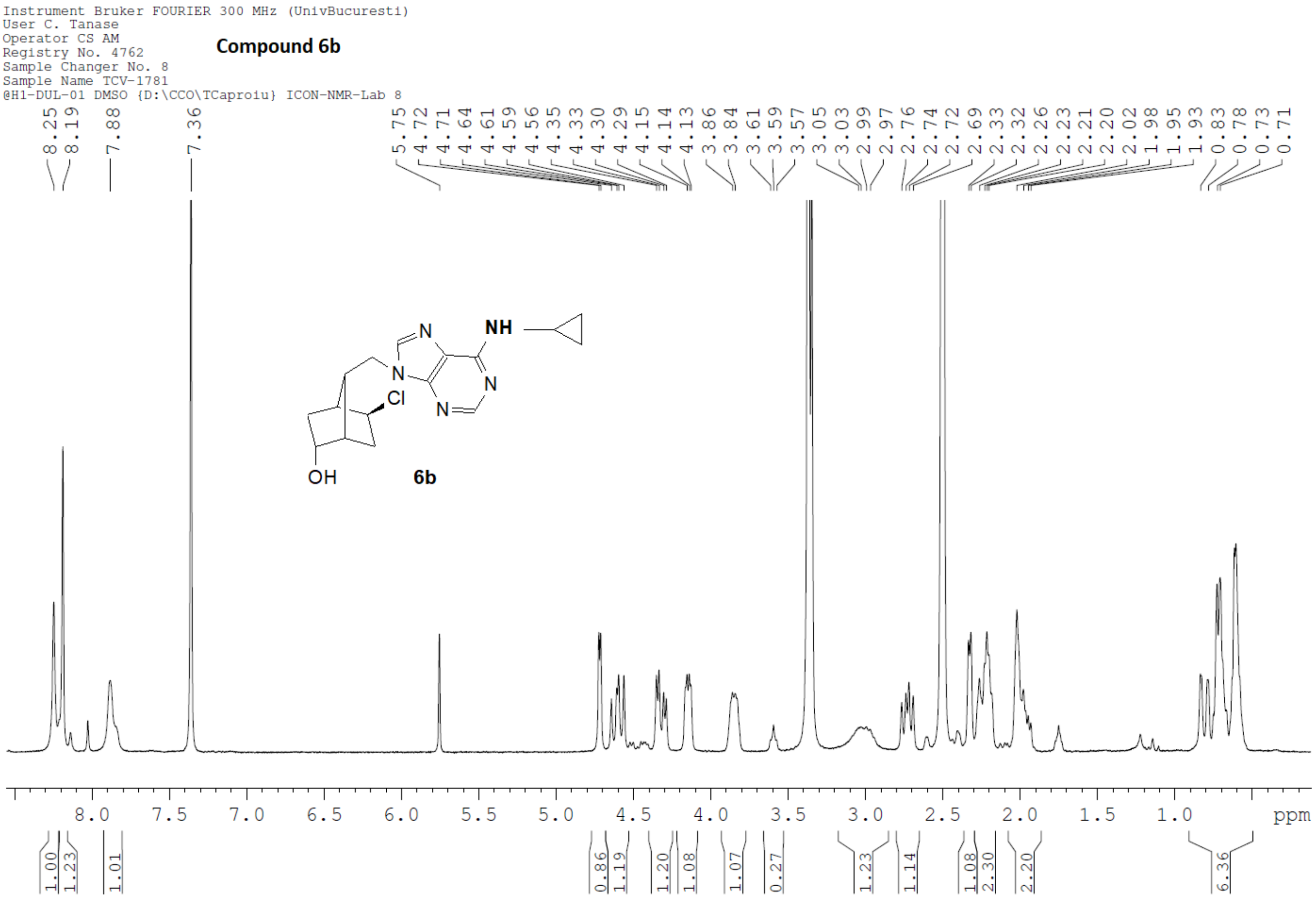

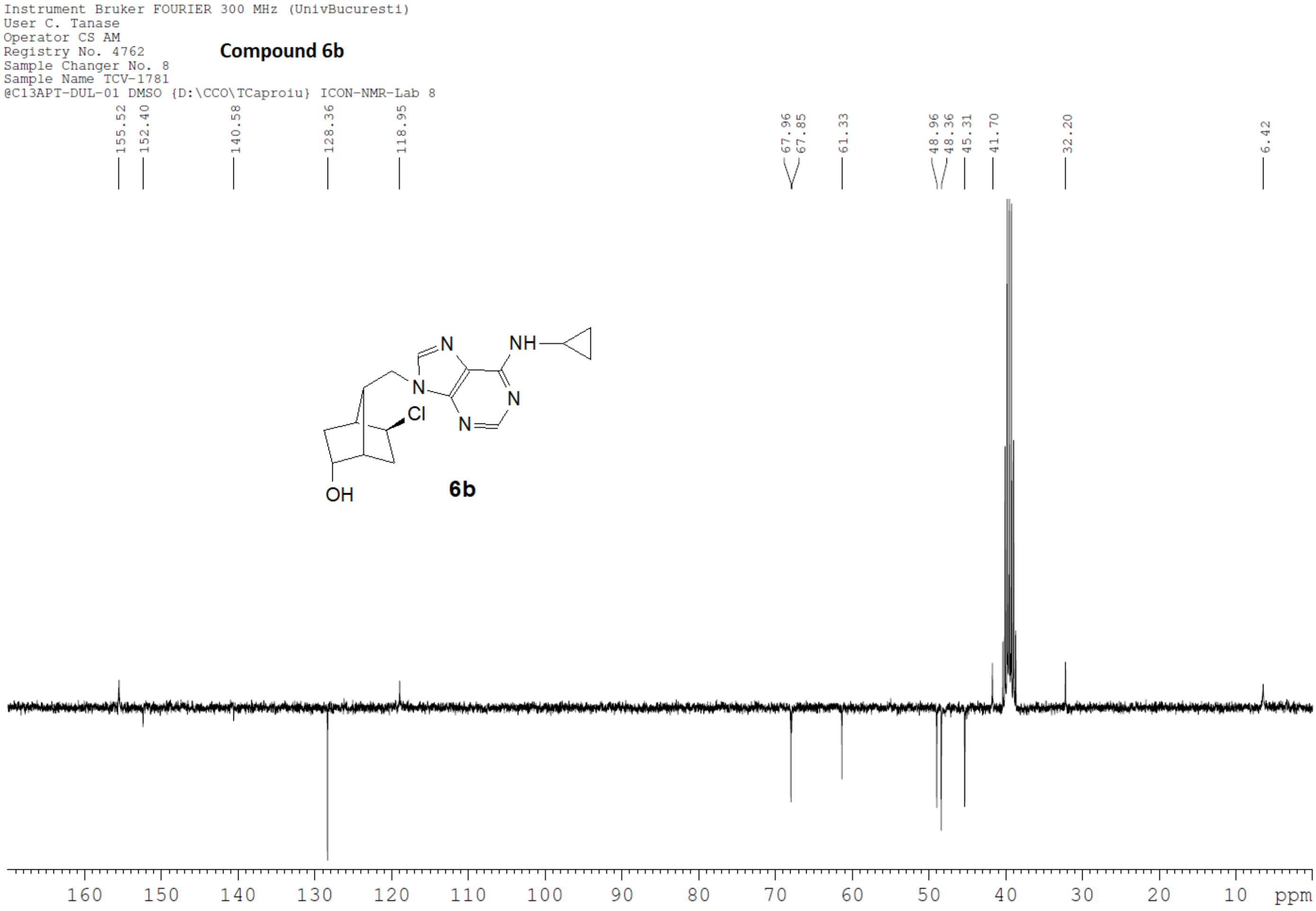

Instrument Bruker FOURIER 300 MHz (UnivBucuresti)
User C. Tanase
Operator CS AM
Registry No. 4762
Sample Changer No. 8
Sample Name TCV-1781
@C13-CPD-DUL-01 DMSO {D:\CCO\TCaproiu} ICON-NMR-Lab 8

Compound 6b

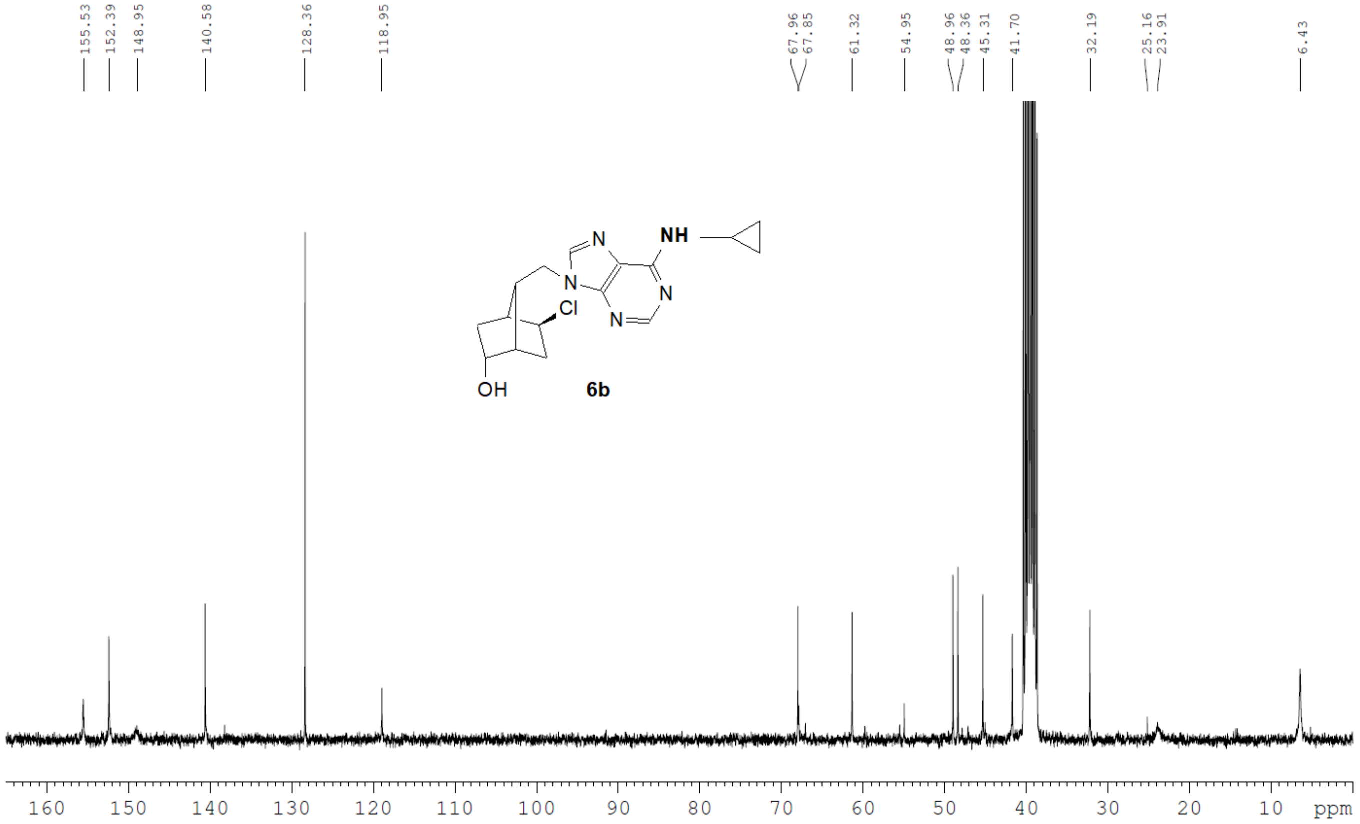

Instrument Bruker FOURIER 300 MHz (UnivBucuresti)
User C. Tanase
Operator CS AM
Registry No. 4762
Sample Changer No. 8
Sample Name TCV-1781
@COSYgs-DUL-01 DMSO {D:\CCO\TCaproiu} ICON-NMR-Lab 8

Compound 6b

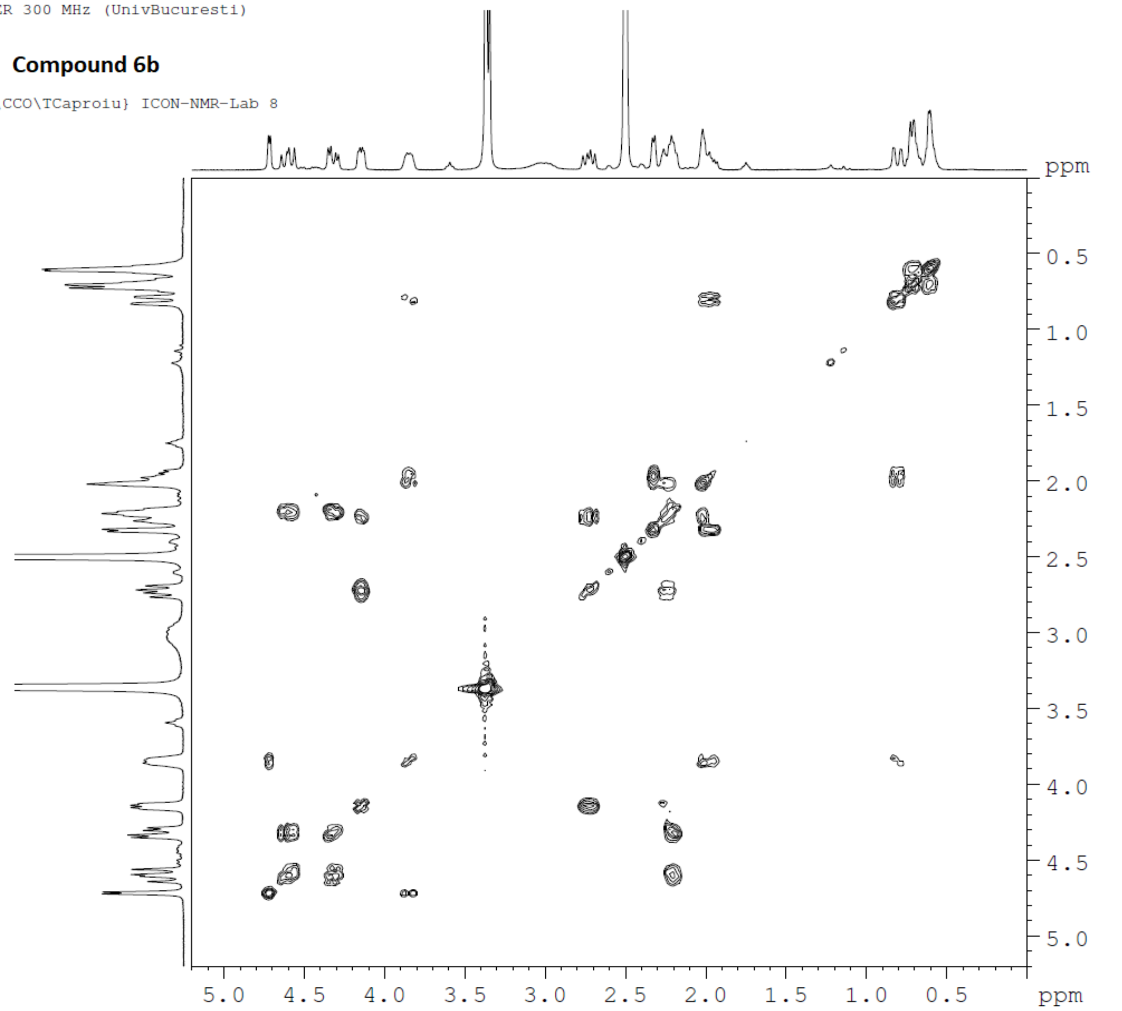

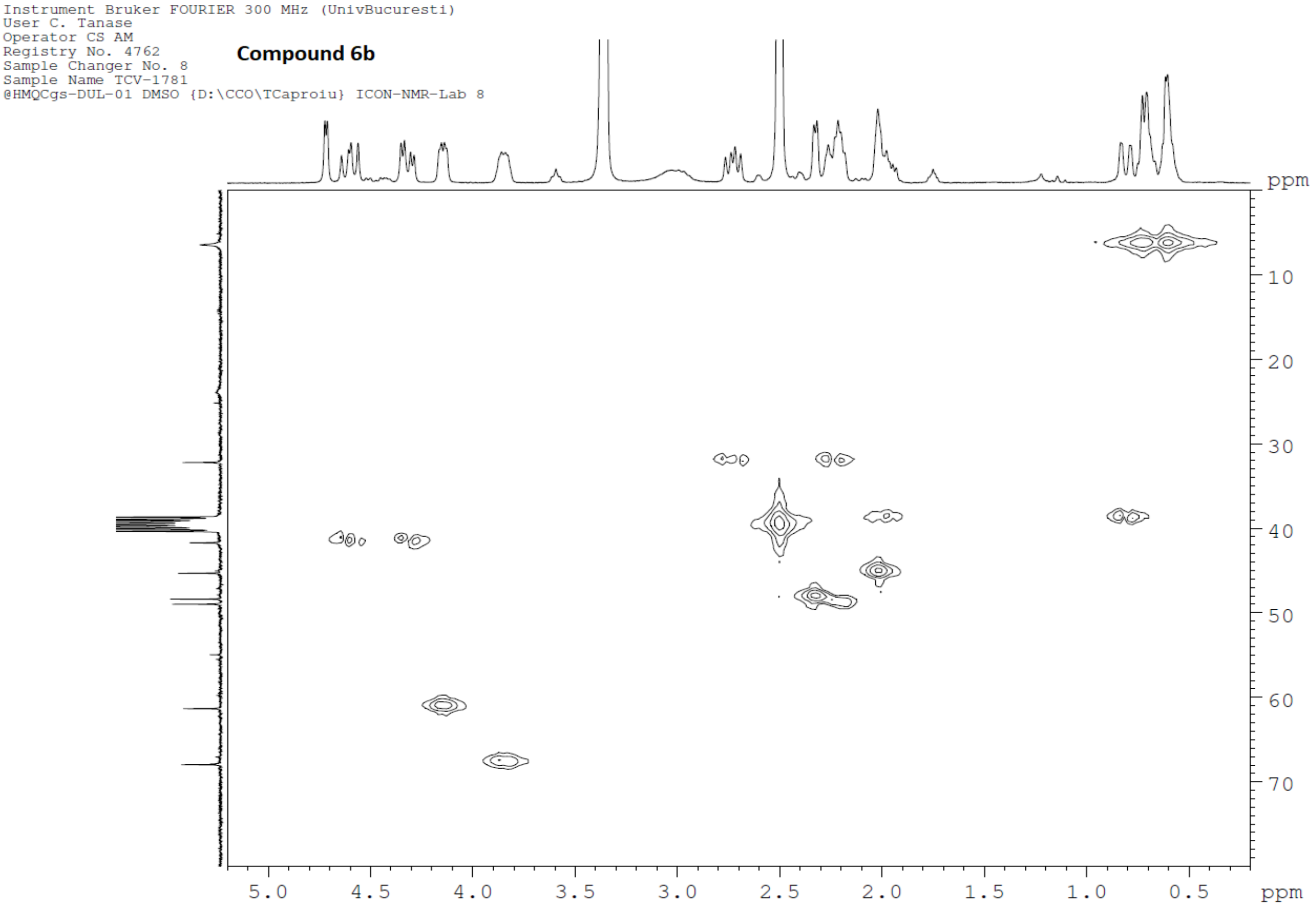

1.8. ^{1}H, ^{13}C, COSY and HETCOR (aliphatic)-NMR spectra in DMSO of the compound **6c**

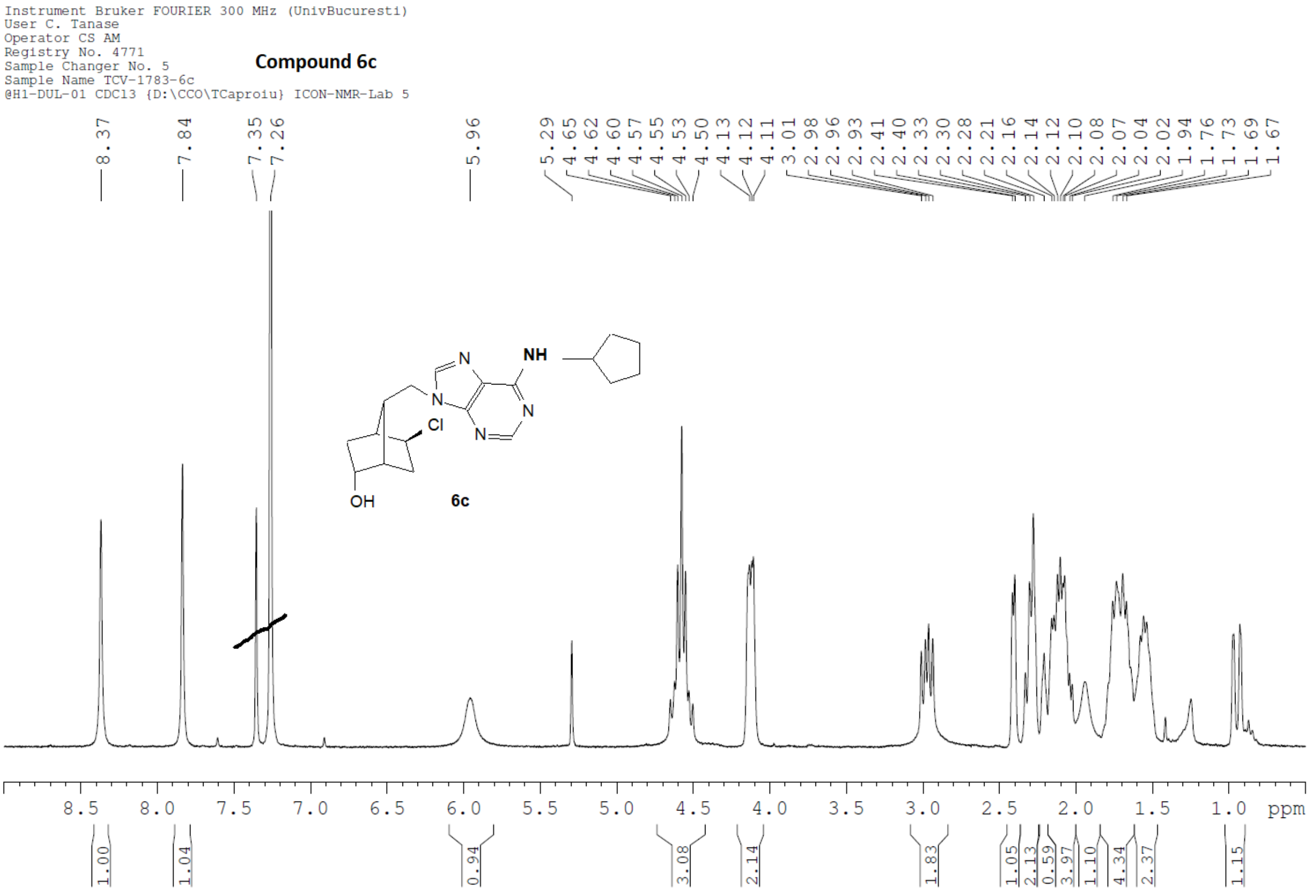

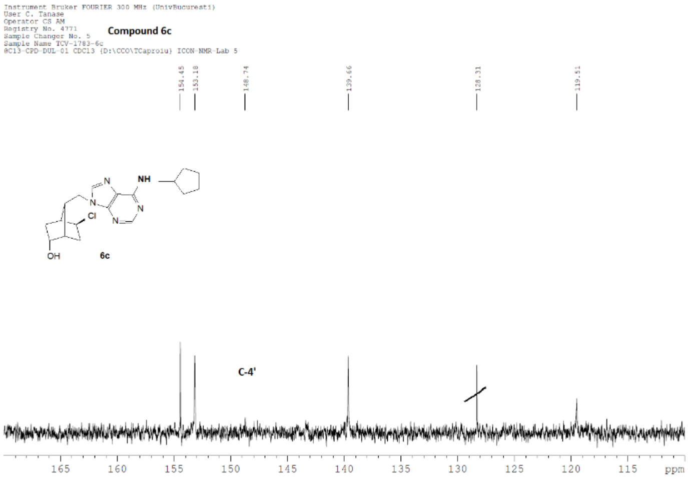
Instrument Bruker FOURIER 300 MHz (UnivBucuresti)
User C. Tanase
Operator CS AM
Registry No. 4771
Sample Changer No. 5
Sample Name TCV-1783-6c
@C13-CPD-DUL-01 CDCl3 {D:\CCO\TCaproiu} ICON-NMR-Lab 5
Compound 6c
154.45
153.18
148.74
139.66
128.31
119.51
NH
N
Cl
OH
6c
C-4'
165
160
155
150
145
140
135
130
125
120
115
ppm

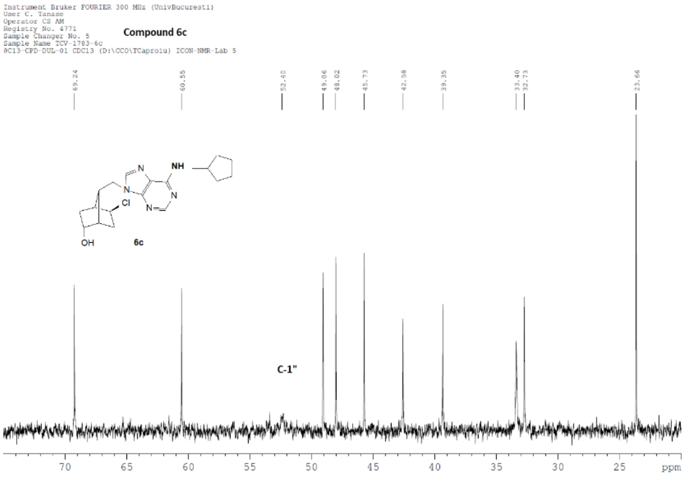
Instrument Bruker FOURIER 300 MHz (UnivBucuresti)
User C. Tanase
Operator CS AM
Registry No. 4771
Sample Changer No. 5
Sample Name TCV-1783-6c
@C13-CPD-DUL-01 CDCl3 {D:\CCO\TCaproiu} ICON-NMR-Lab 5
Compound 6c
69.24
60.55
52.40
49.06
48.02
45.73
42.58
39.35
33.40
32.73
23.66
NH
N
Cl
OH
6c
C-1''
70
65
60
55
50
45
40
35
30
25
ppm

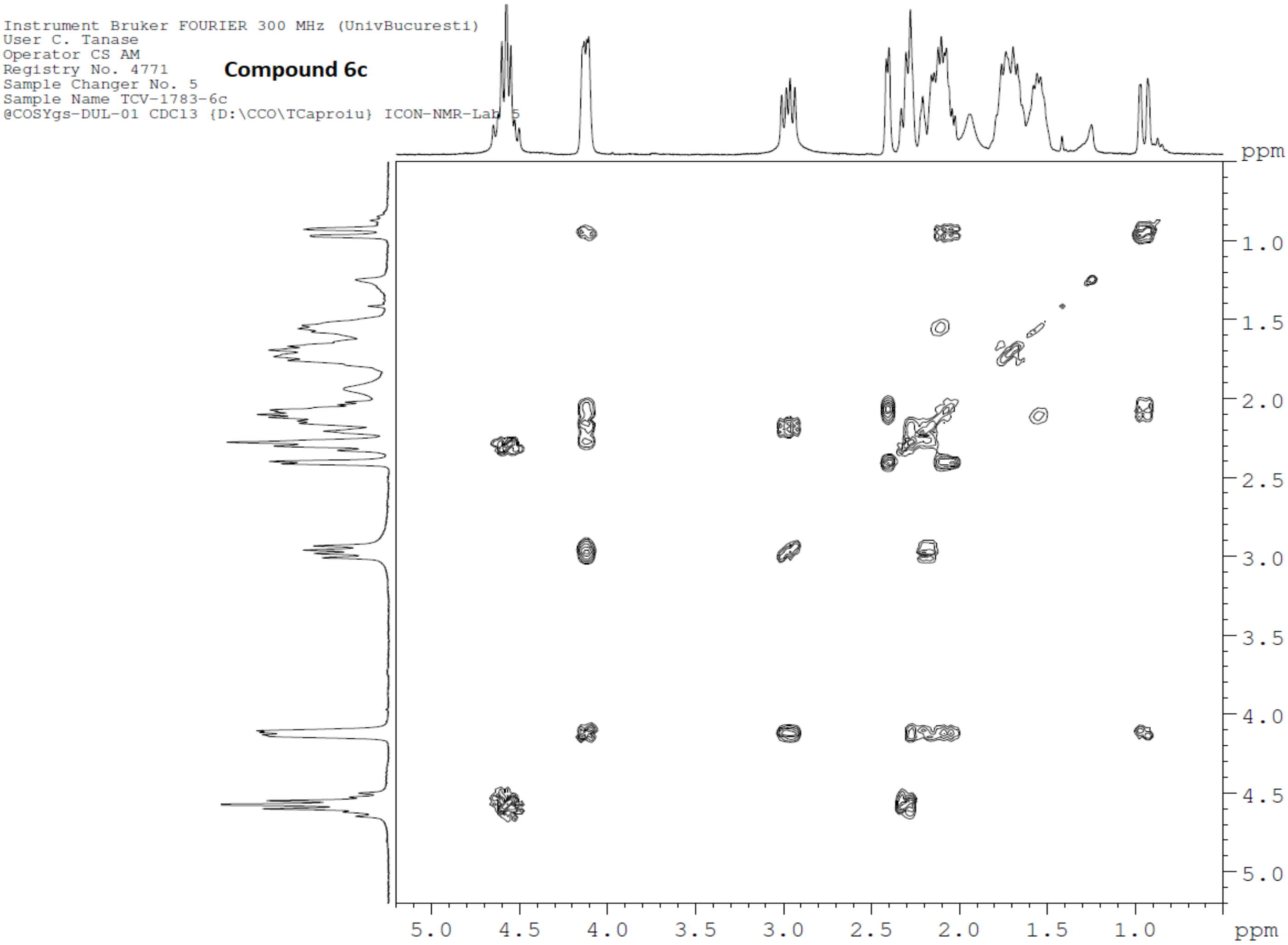
Instrument Bruker FOURIER 300 MHz (UnivBucuresti)
User C. Tanase
Operator CS AM
Registry No. 4771
Sample Changer No. 5
Sample Name TCV-1783-6c
@COSYgs-DUL-01 CDCl3 {D:\CCO\TCaproiu} ICON-NMR-Lab 5
Compound 6c
ppm
1.0
1.5
2.0
2.5
3.0
3.5
4.0
4.5
5.0
5.0
4.5
4.0
3.5
3.0
2.5
2.0
1.5
1.0
ppm

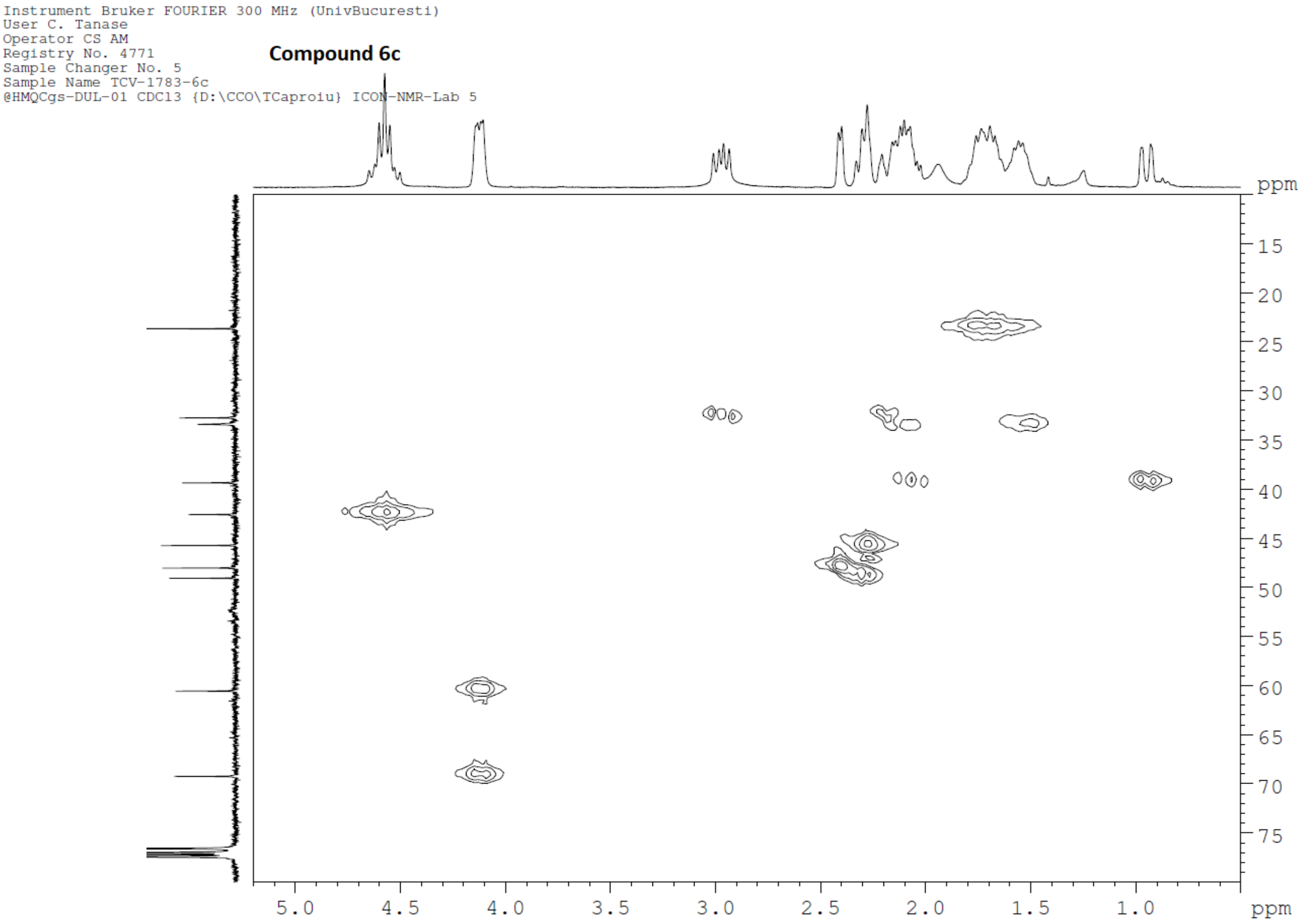

1.9. ^{1}H, ^{13}C, COSY and HETCOR (aliphatic)-NMR spectra in DMSO of the compound **6d**

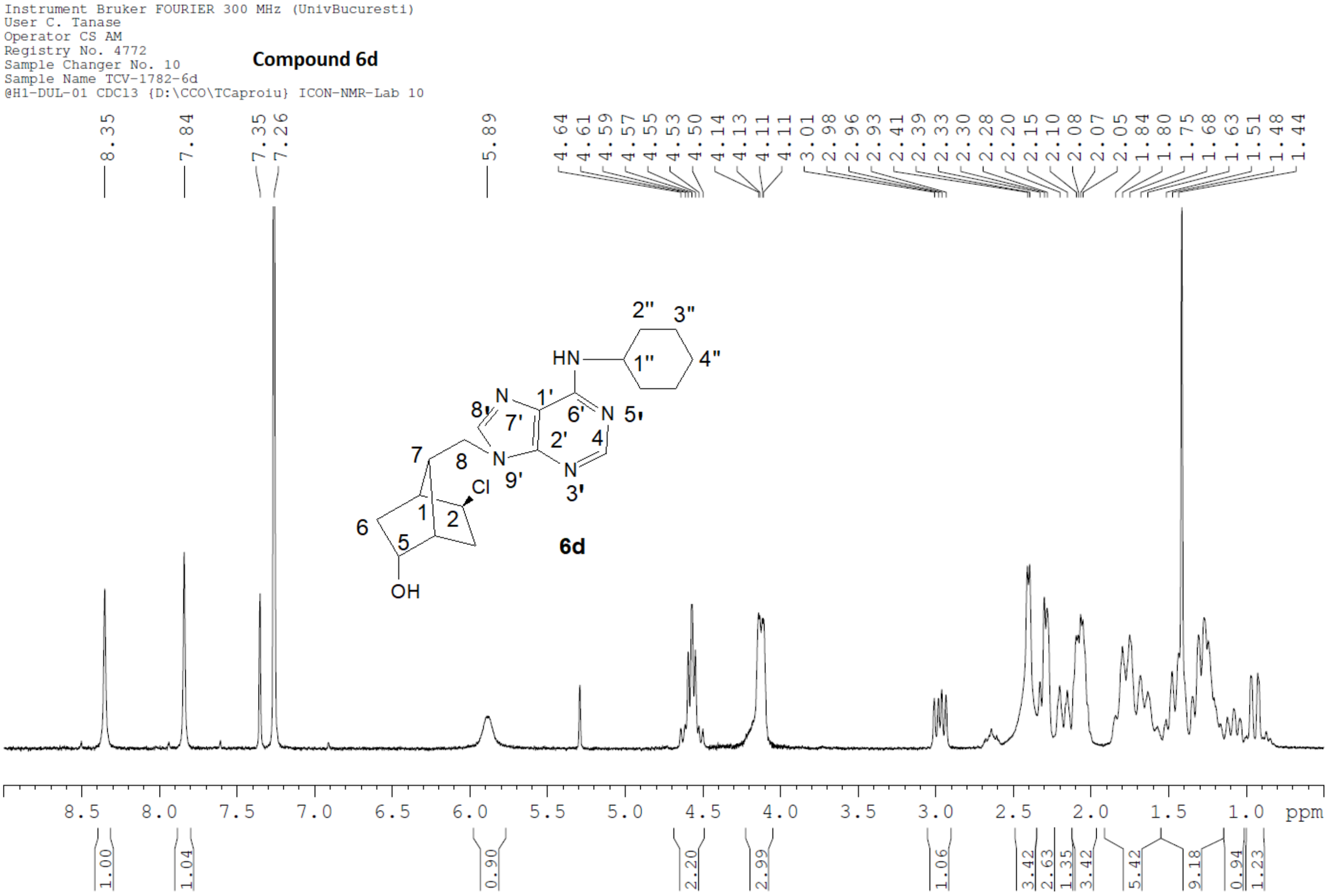

Instrument Bruker FOURIER 300 MHz (UnivBucuresti)
User C. Tanase
Operator CS AM
Registry No. 4772
Sample Changer No. 10
Sample Name TCV-1782-6d
@C13APT-DUL-01 CDCl3 {D:\CCO\TCaproiu} ICON-NMR-Lab 10

Compound 6d

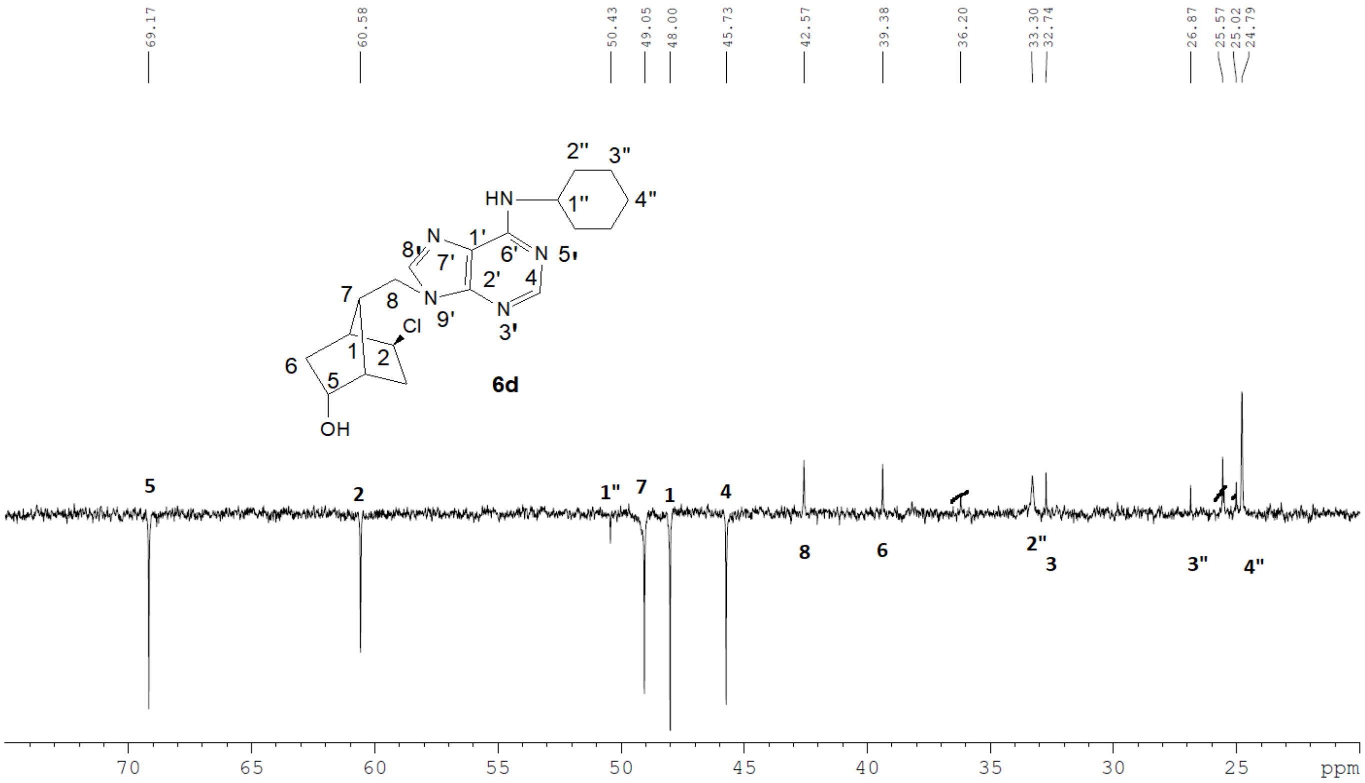

Instrument Bruker FOURIER 300 MHz (UnivBucuresti)
User C. Tanase
Operator CS AM
Registry No. 4772
Sample Changer No. 10
Sample Name TCV-1782-6d
@COSYgs-DUL-01 CDCl3 {D:\CCO\TCaproiu} ICON-NMR-Lab 10

Compound 6d

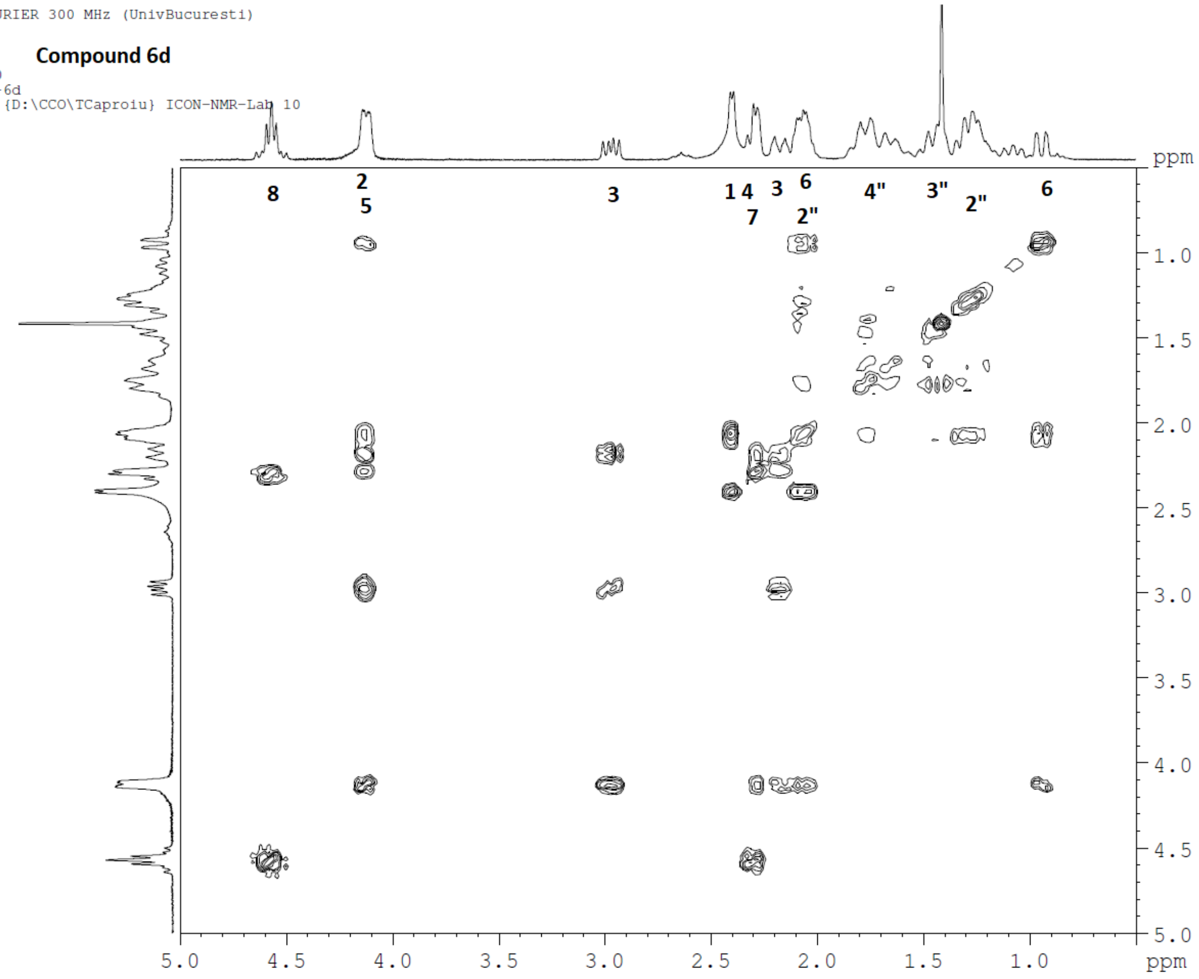

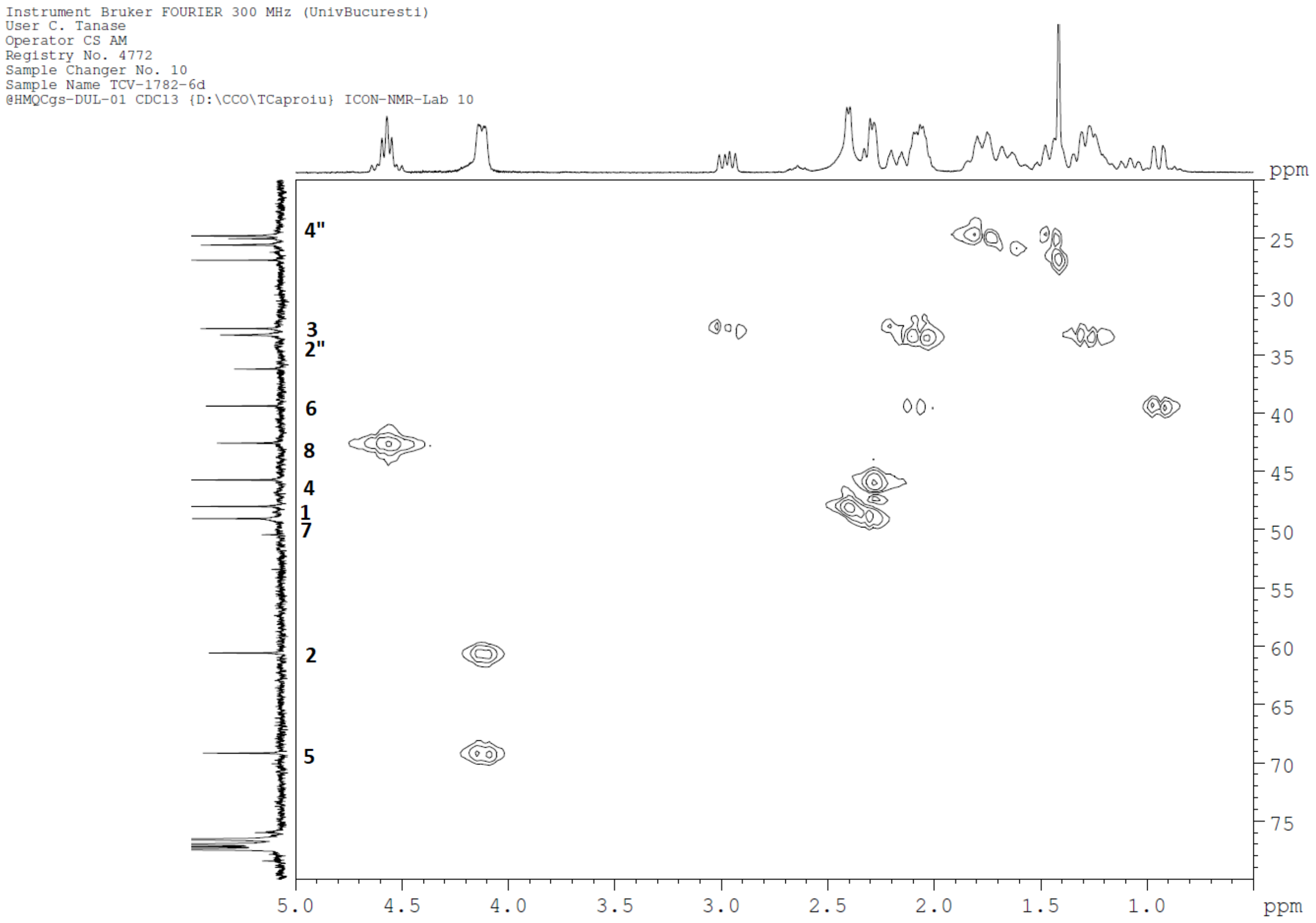

1.10. ^{1}H, ^{13}C, COSY and HETCOR (aliphatic)-NMR spectra in DMSO of the compound **6e**

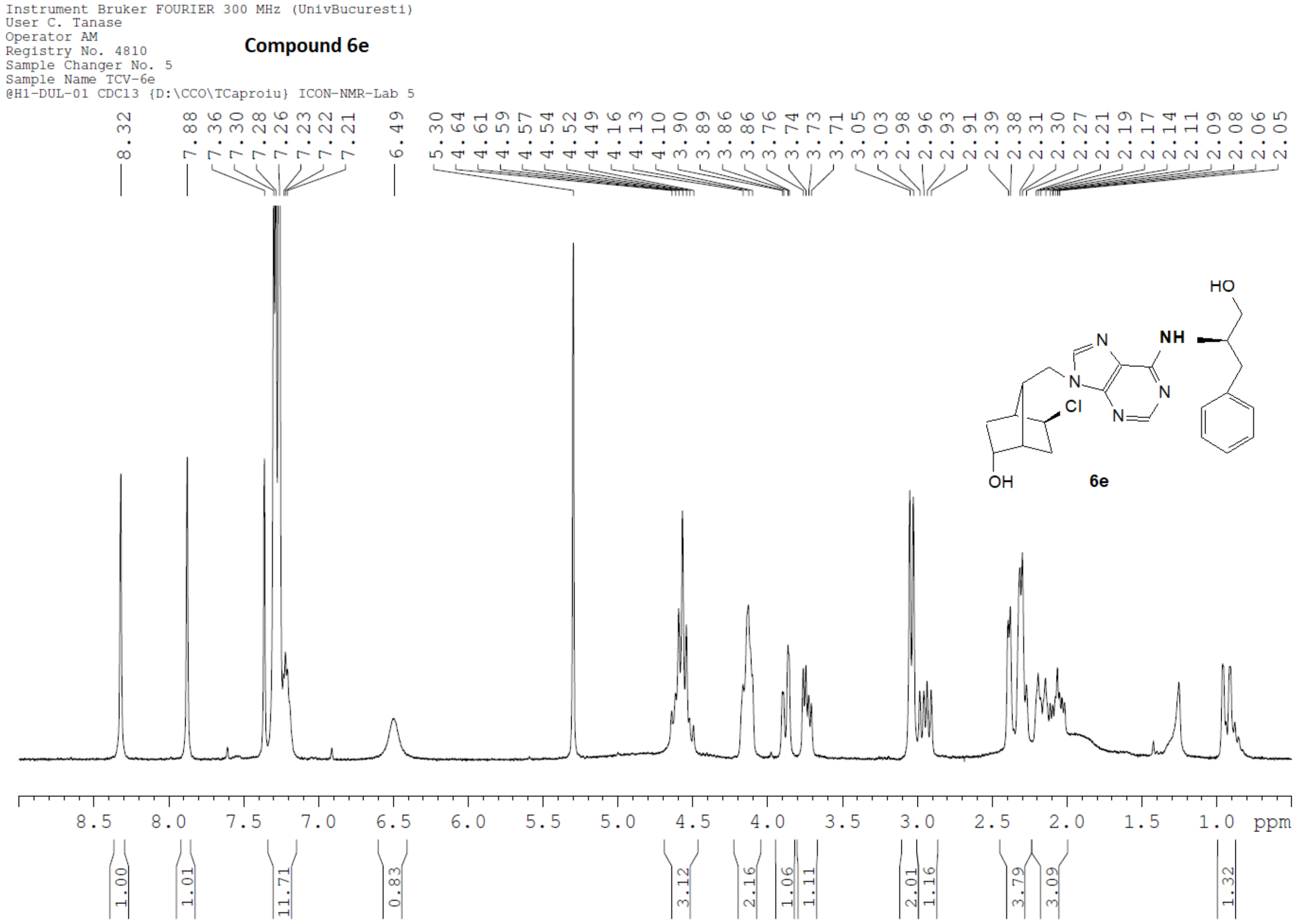

Instrument Bruker FOURIER 300 MHz (UnivBucuresti)
User C. Tanase
Operator AM
Registry No. 4810
Sample Changer No. 5
Sample Name TCV-6e
+TFA
@H1-DUL-01 CDC13 {D:\CCO\TCaproiu} ICON-NMR-Lab 5

Compound 6e, Proton spectrum + TFA

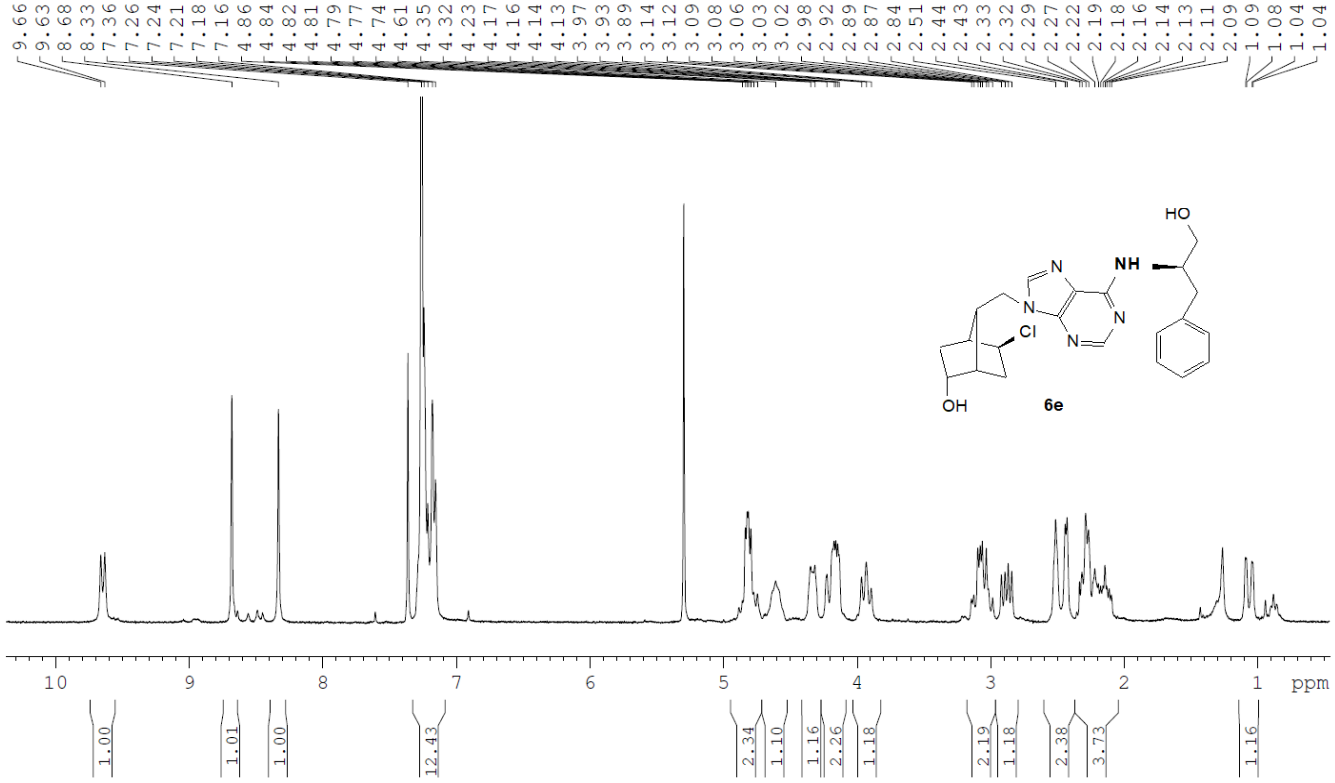

Instrument Bruker FOURIER 300 MHz (UnivBucuresti)
User C. Tanase
Operator AM
Registry No. 4810
Sample Changer No. 5
Sample Name TCV-6e
@C13-CPD-DUL-01 CDC13 {D:\CCO\TCaproiu} ICON-NMR-Lab 5

Compound 6e

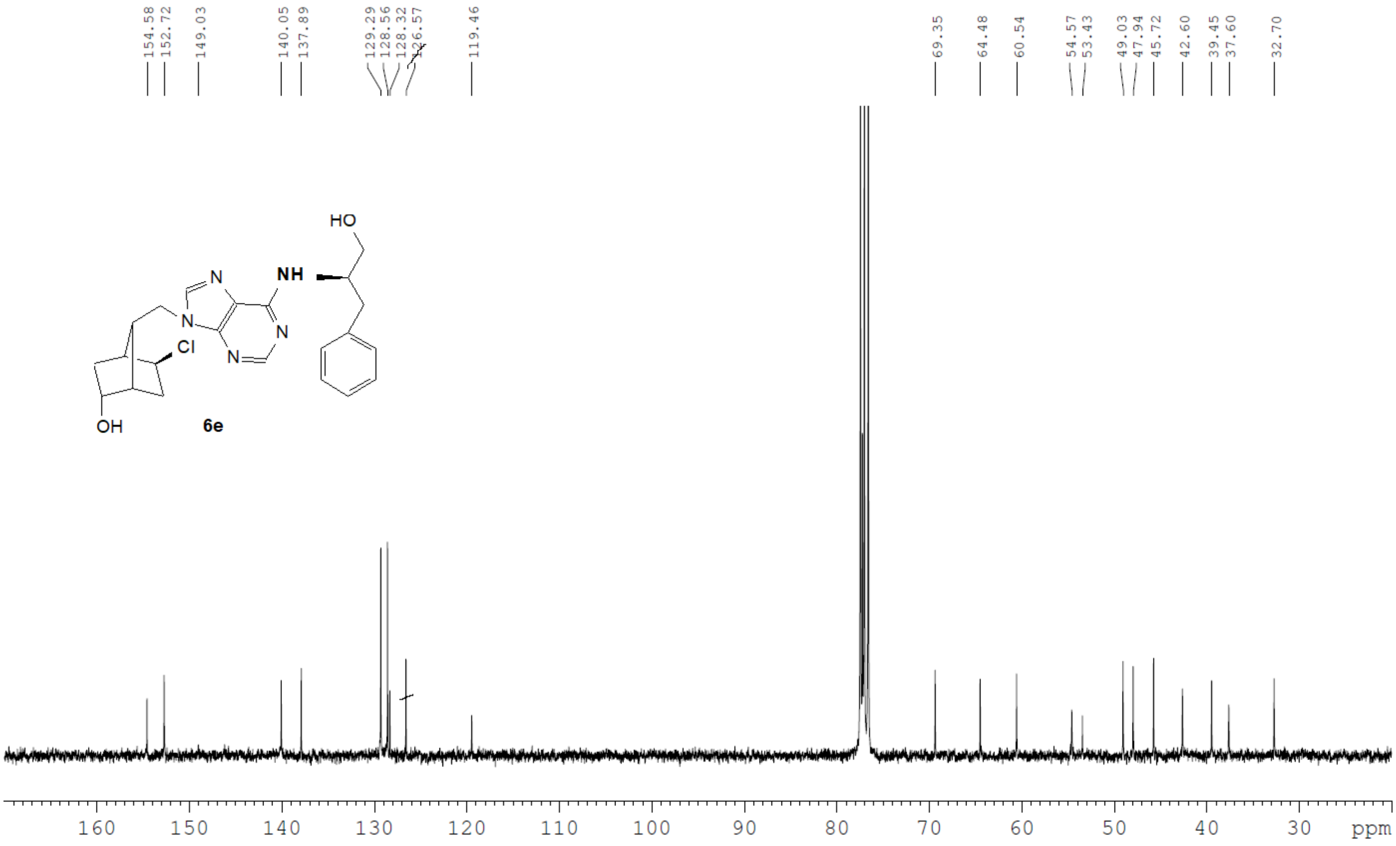

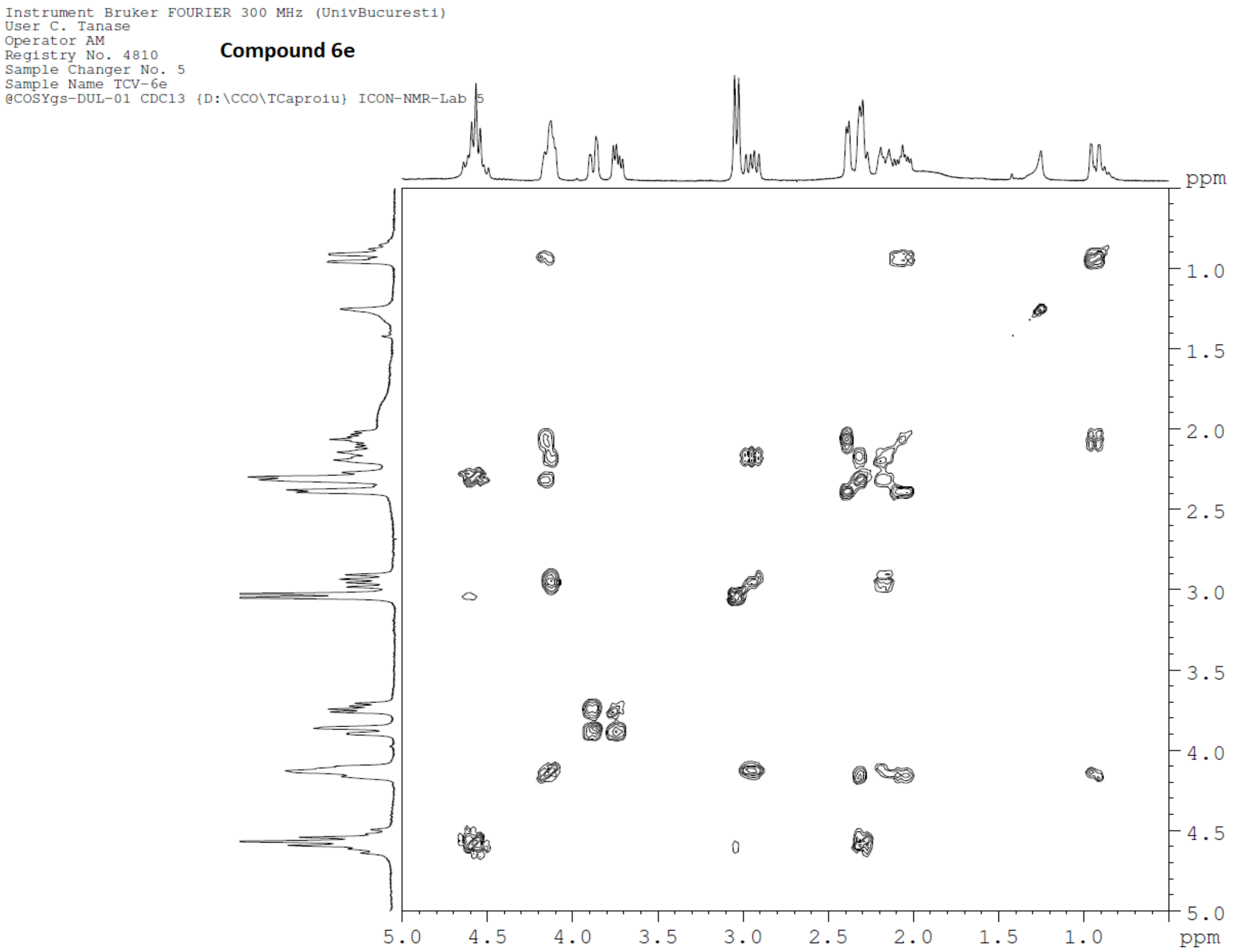
Instrument Bruker FOURIER 300 MHz (UnivBucuresti)
User C. Tanase
Operator AM
Registry No. 4810
Sample Changer No. 5
Sample Name TCV-6e
@COSYgs-DUL-01 CDCl3 {D:\CCO\TCaproiu} ICON-NMR-Lab 5
Compound 6e
ppm
1.0
1.5
2.0
2.5
3.0
3.5
4.0
4.5
5.0
5.0
4.5
4.0
3.5
3.0
2.5
2.0
1.5
1.0
ppm

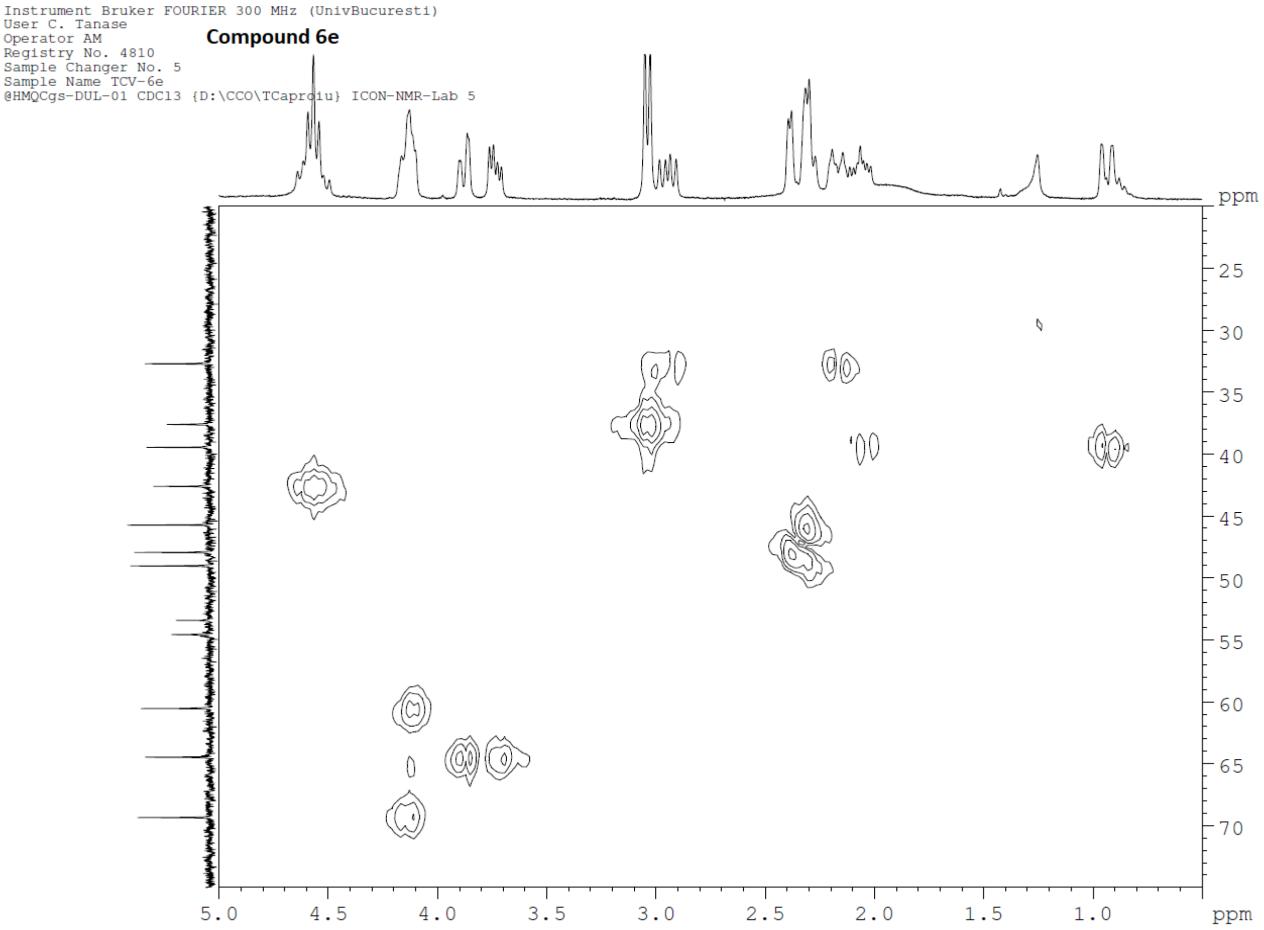
Instrument Bruker FOURIER 300 MHz (UnivBucuresti)
User C. Tanase
Operator AM
Registry No. 4810
Sample Changer No. 5
Sample Name TCV-6e
@HMQCgs-DUL-01 CDCl3 {D:\CCO\TCaproiu} ICON-NMR-Lab 5
Compound 6e
ppm
25
30
35
40
45
50
55
60
65
70
5.0
4.5
4.0
3.5
3.0
2.5
2.0
1.5
1.0
ppm

1.11. ^{1}H, ^{13}C, COSY and HETCOR (aliphatic)-NMR spectra in DMSO of the compound **6f**

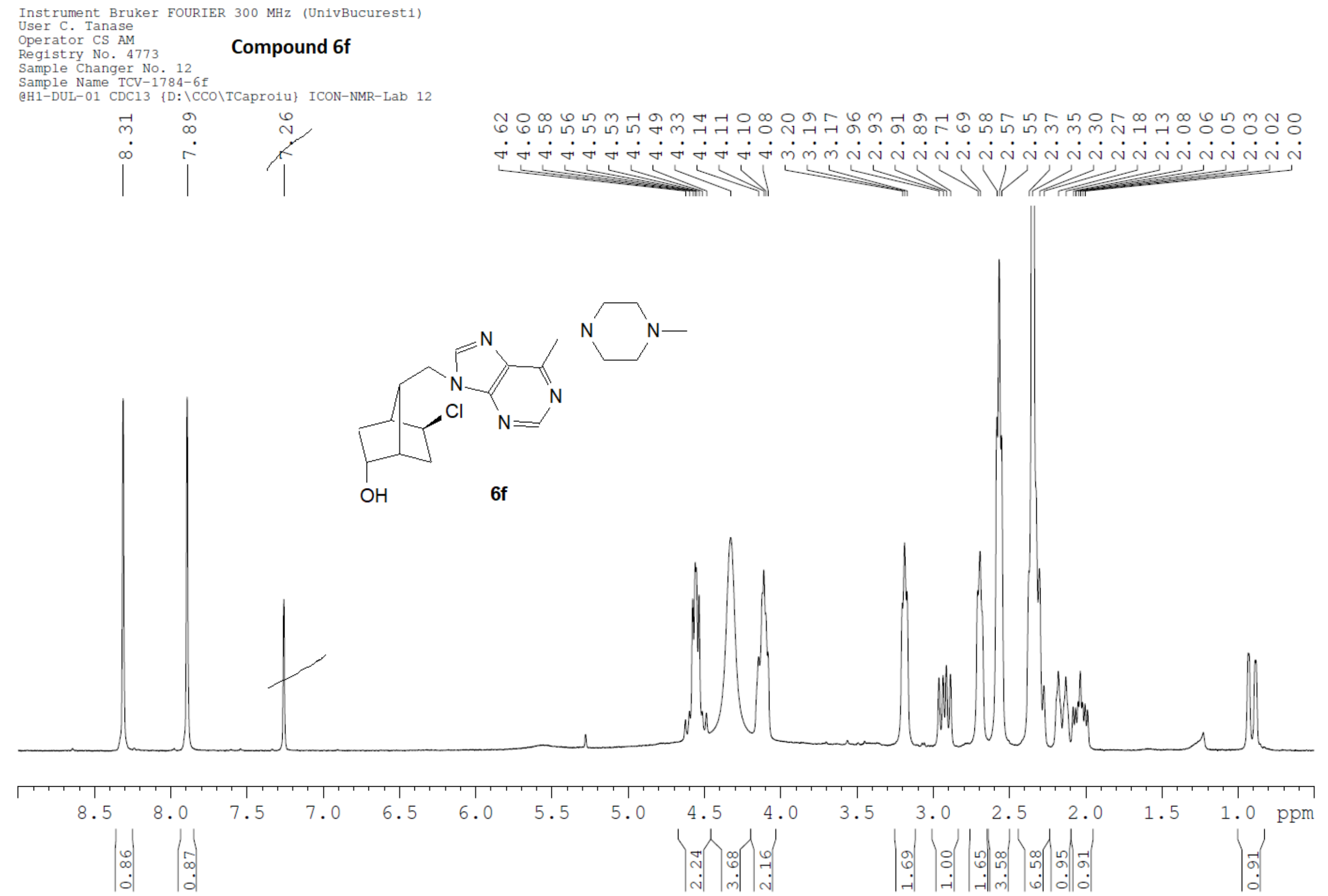

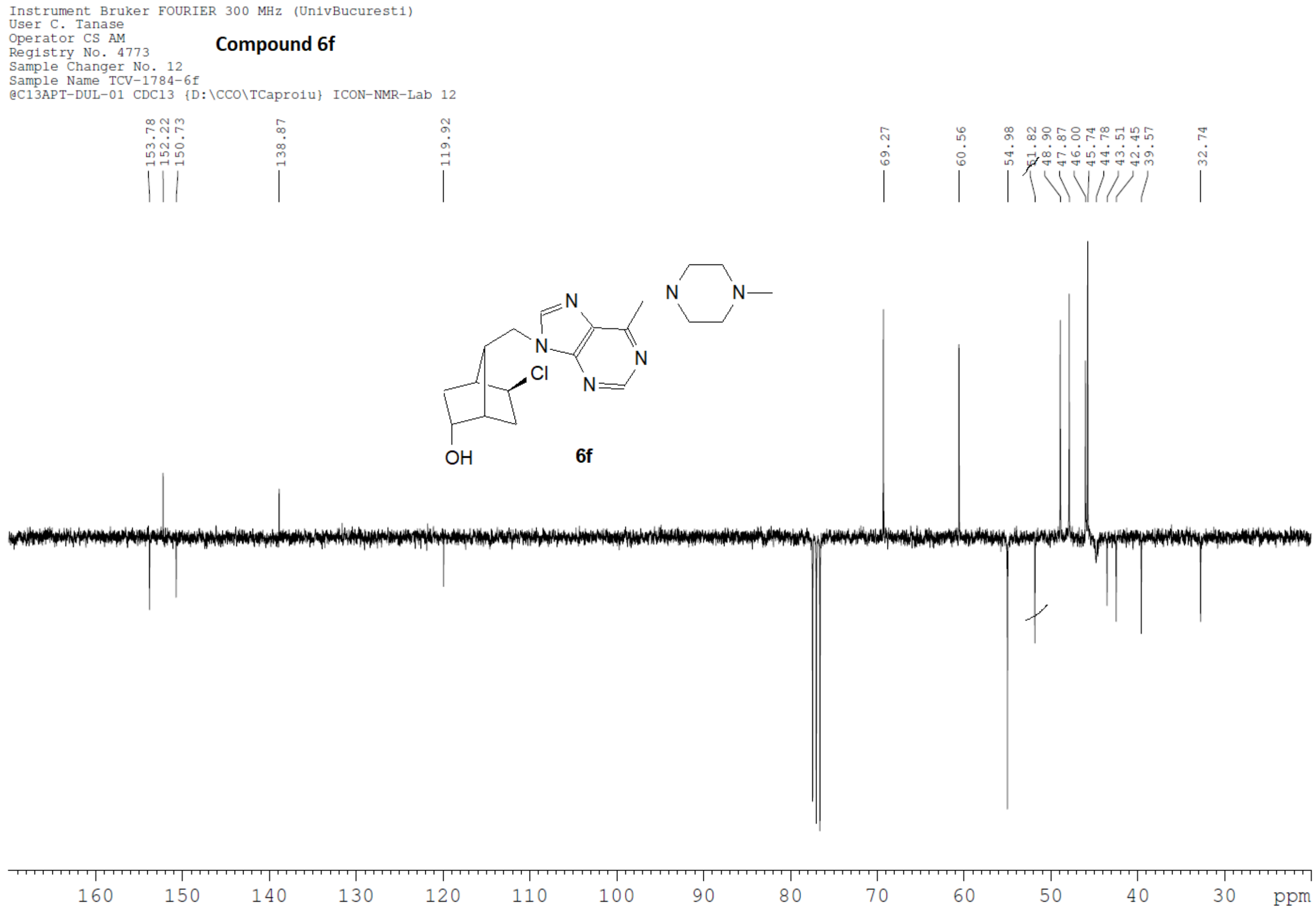

Instrument Bruker FOURIER 300 MHz (UnivBucuresti)
User C. Tanase
Operator CS AM
Registry No. 4773
Sample Changer No. 12
Sample Name TCV-1784-6f
@COSYgs-DUL-01 CDC13 {D:\CCO\TCaproiu} ICON-NMR-Lab 12

Compound 6f

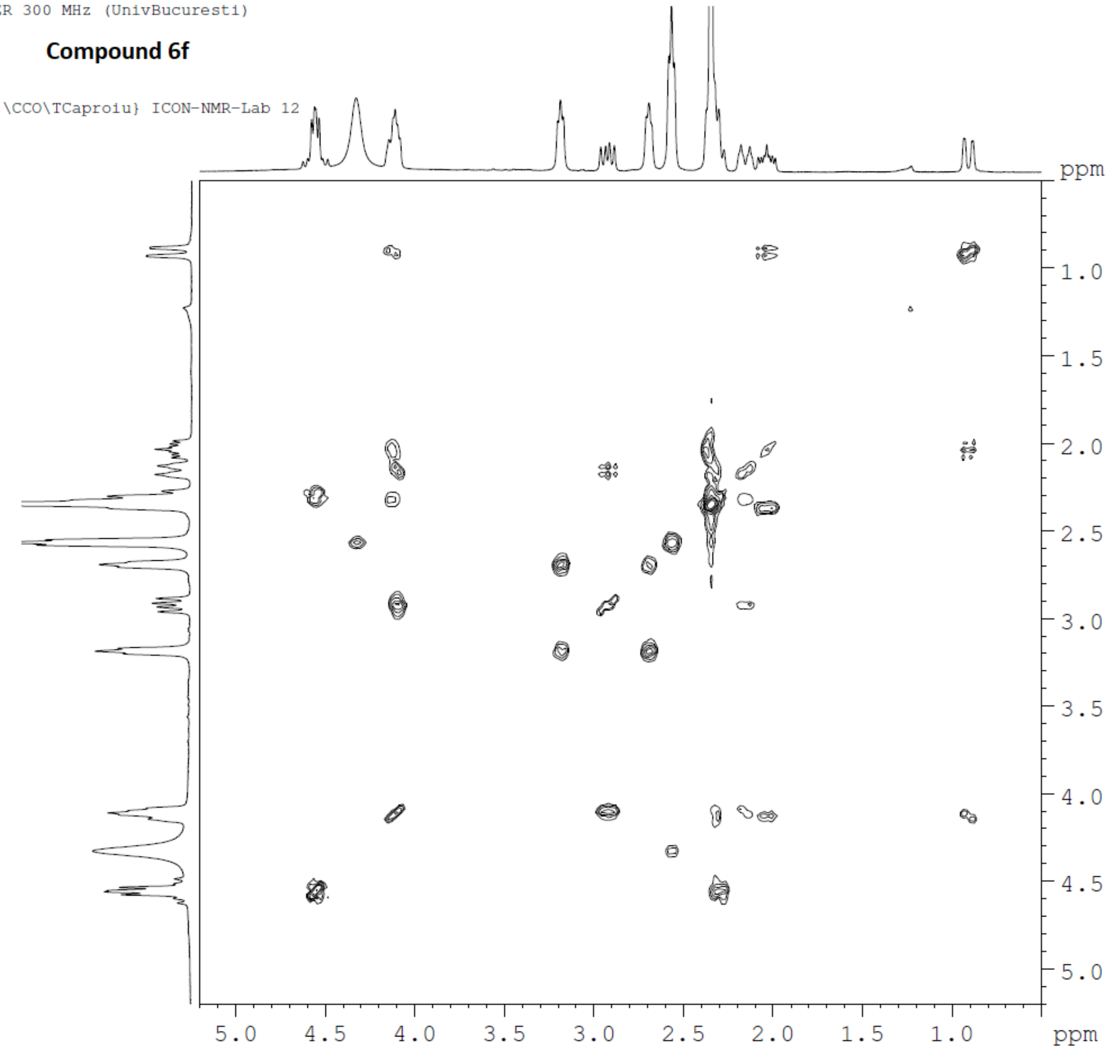

Instrument Bruker FOURIER 300 MHz (UnivBucuresti)
User C. Tanase
Operator CS AM
Registry No. 4773
Sample Changer No. 12
Sample Name TCV-1784-6f
@HMQCgs-DUL-01 CDC13 {D:\CCO\TCaproiu} ICON-NMR-Lab 12

Compound 6f

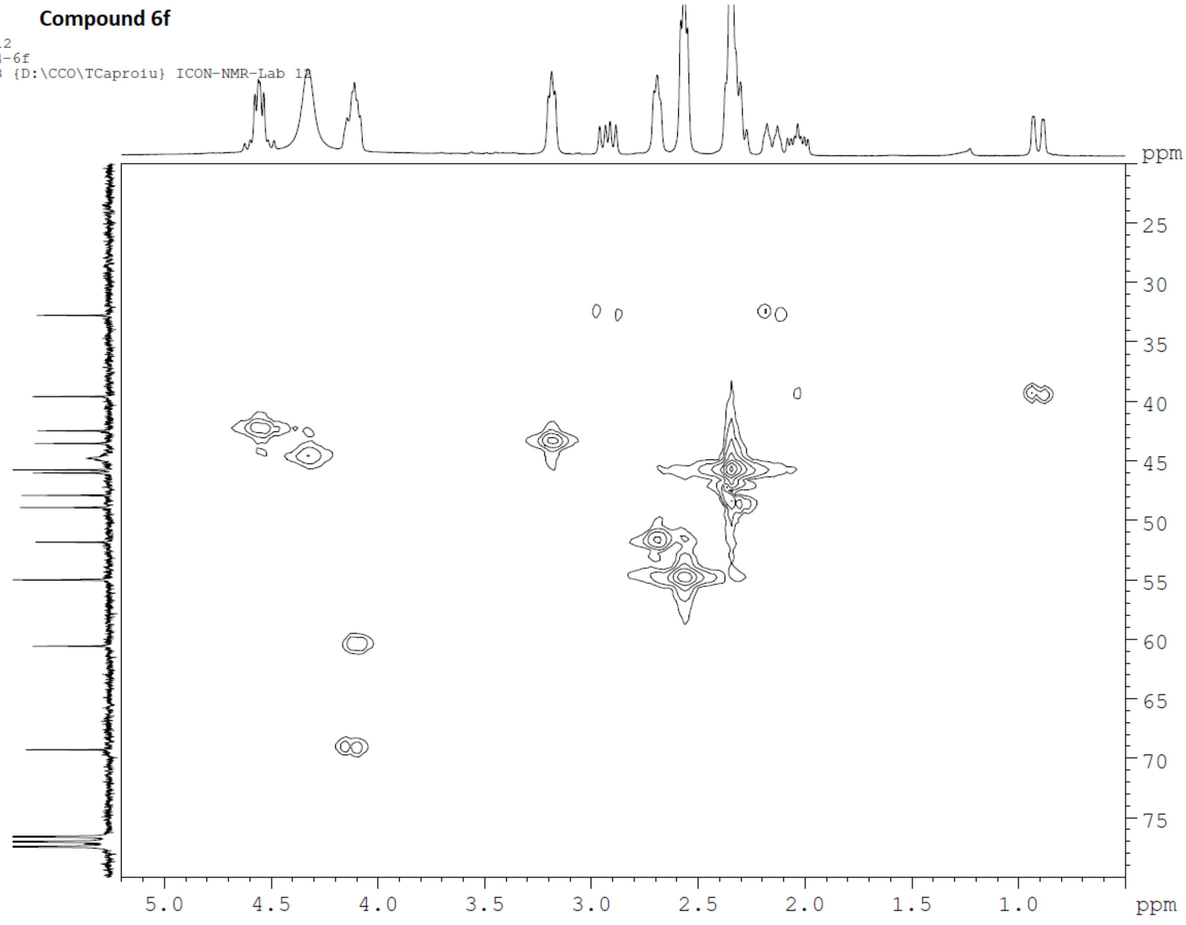

1.12. ^{1}H, ^{13}C, COSY and HETCOR (aliphatic)-NMR spectra in DMSO of the compound **6g**

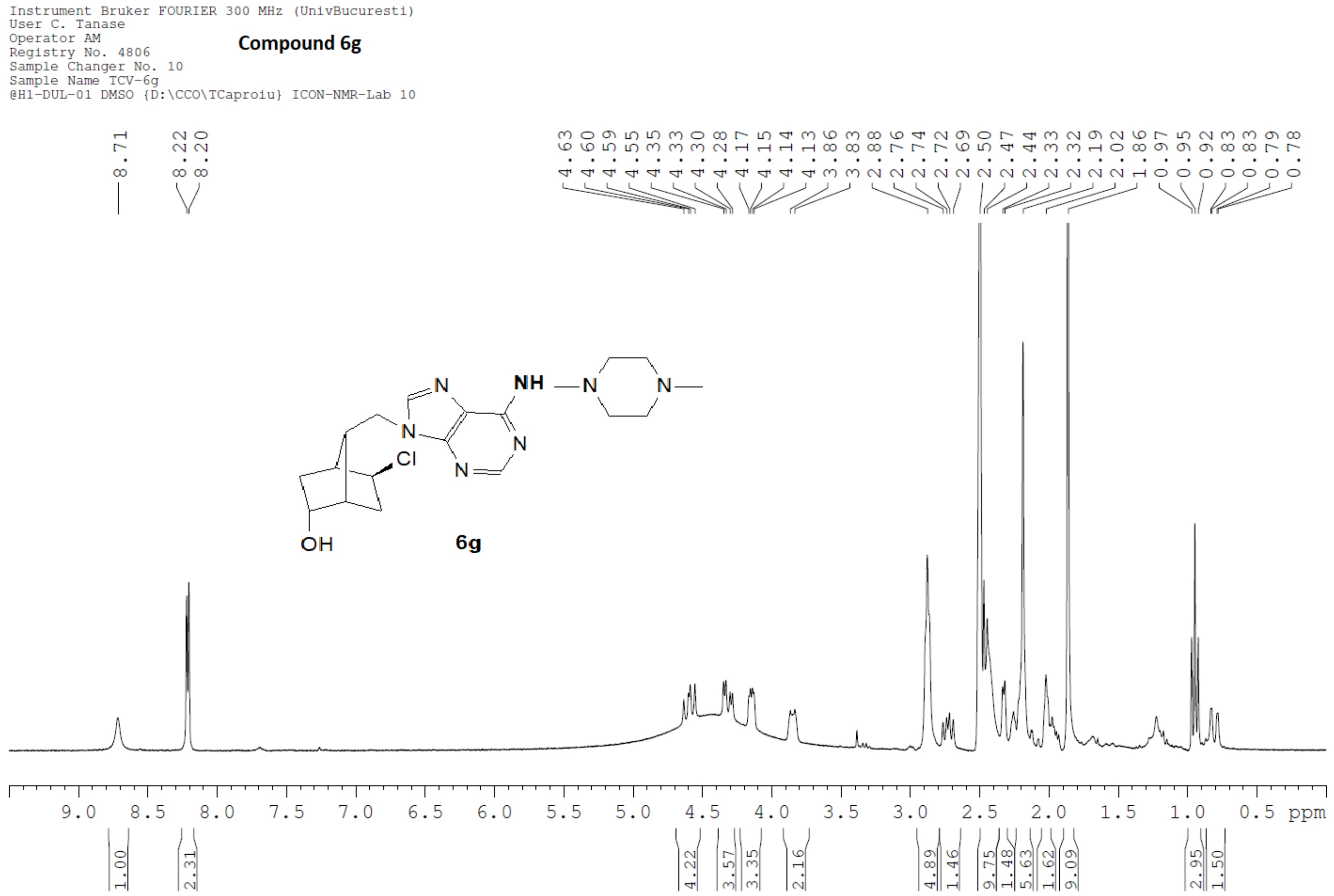

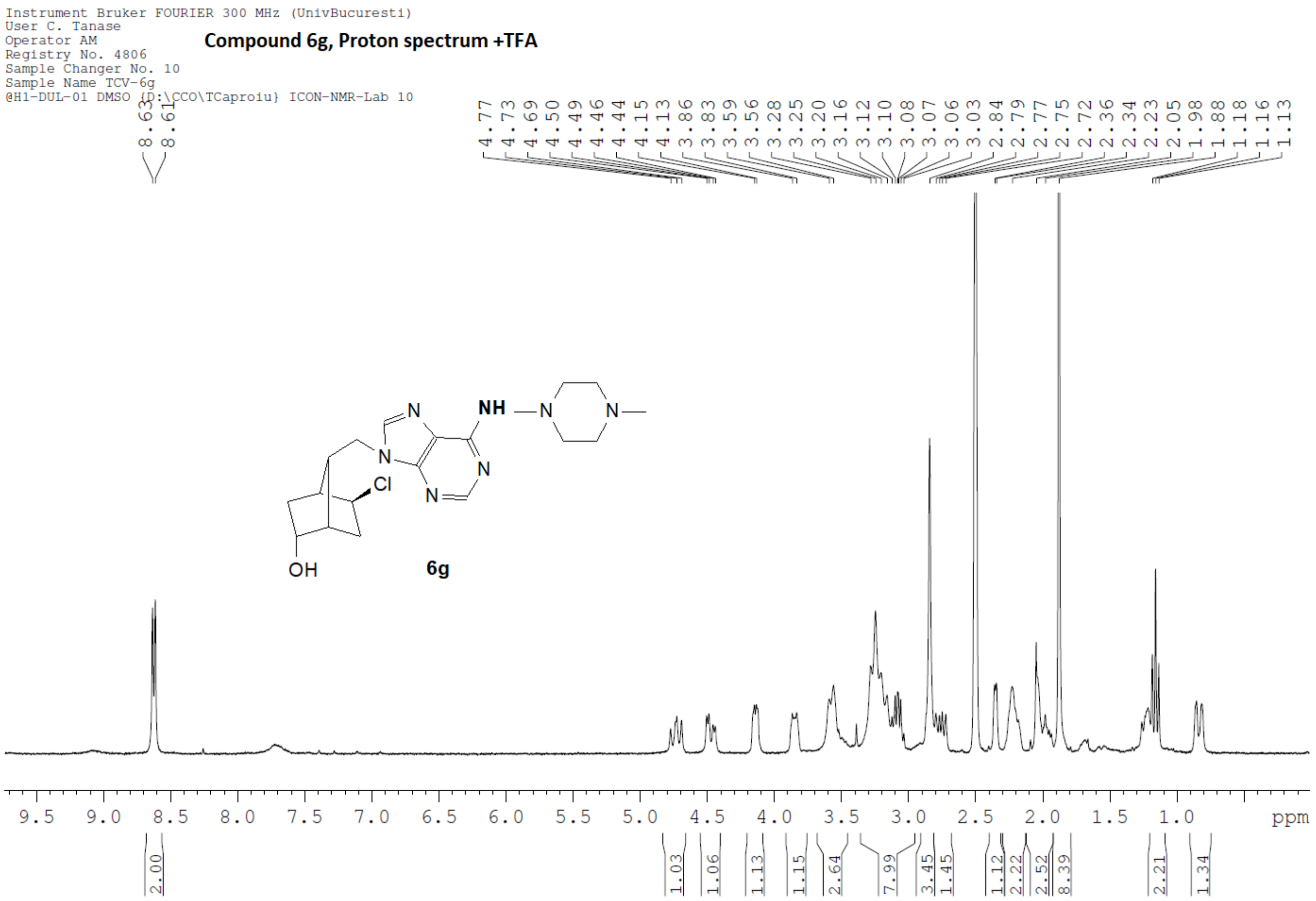

Instrument Bruker FOURIER 300 MHz (UnivBucuresti)
User C. Tanase
Operator AM
Registry No. 4806
Sample Changer No. 10
Sample Name TCV-6g
@COSYgs-DUL-01 DMSO {D:\CCO\TCaproiu} ICON-NMR-Lab 10

Compound 6g

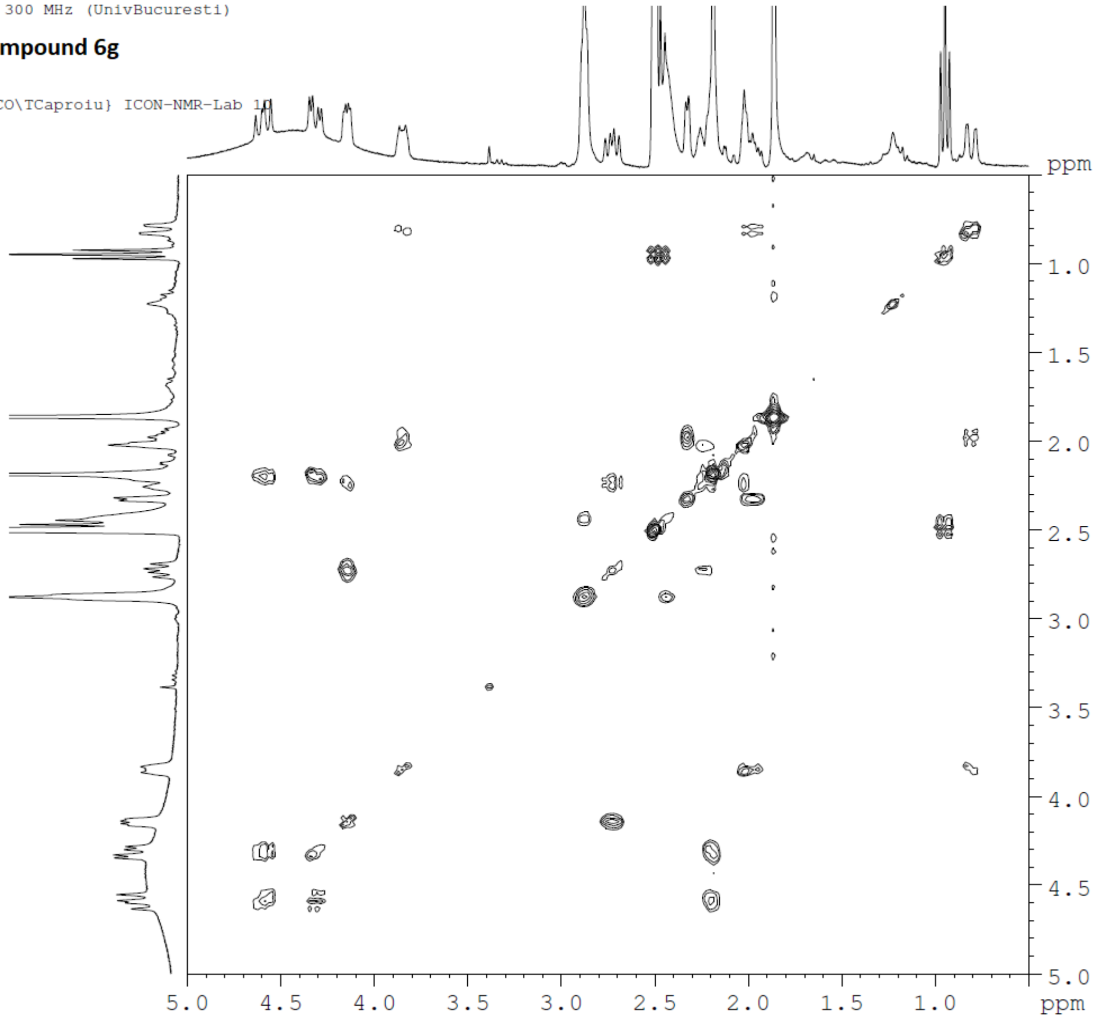

Instrument Bruker FOURIER 300 MHz (UnivBucuresti)
User C. Tanase
Operator AM
Registry No. 4806
Sample Changer No. 10
Sample Name TCV-6g
@HMQCgs-DUL-01 DMSO {D:\CCO\TCaproiu} ICON-NMR-Lab 10

Compound 6g

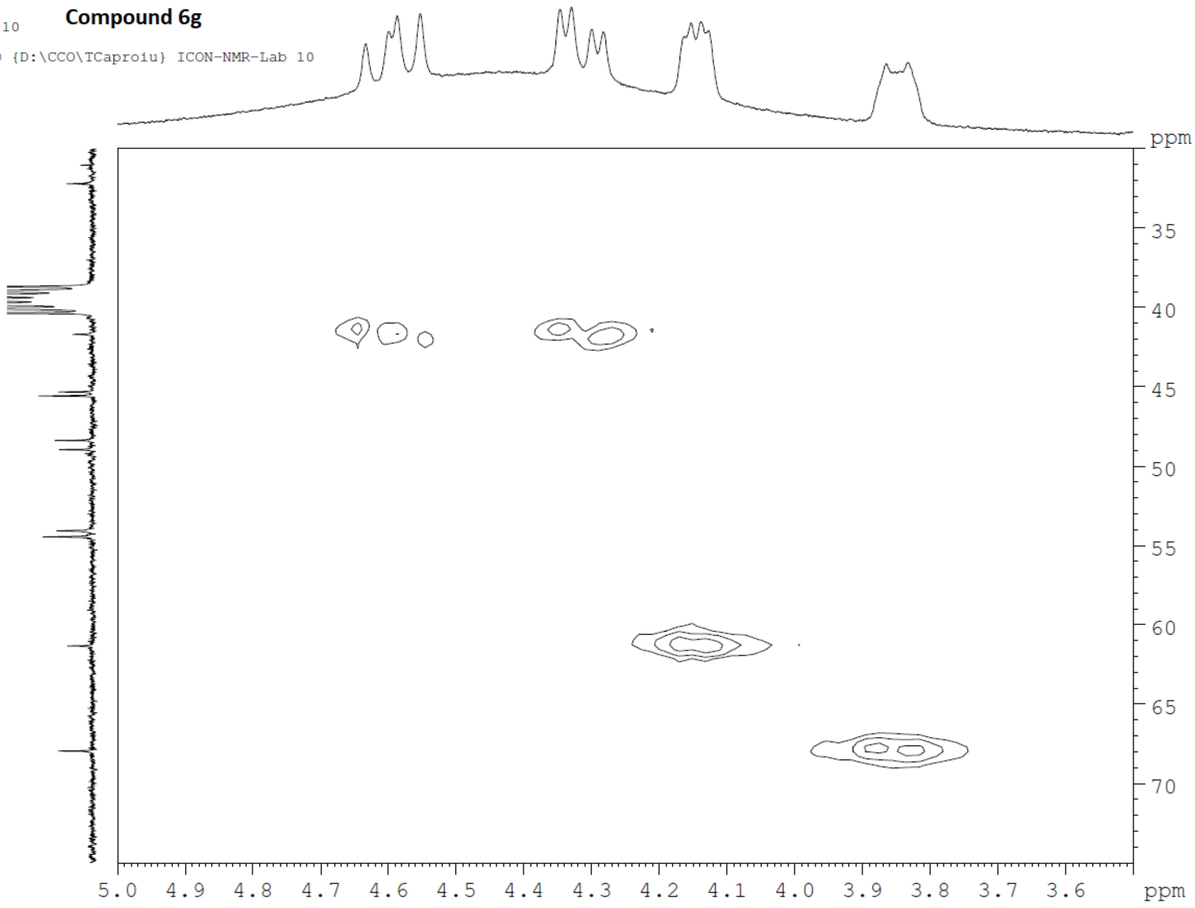

1.13. ^{1}H, ^{13}C, COSY and HETCOR (aliphatic)-NMR spectra in DMSO of the compound **6h**

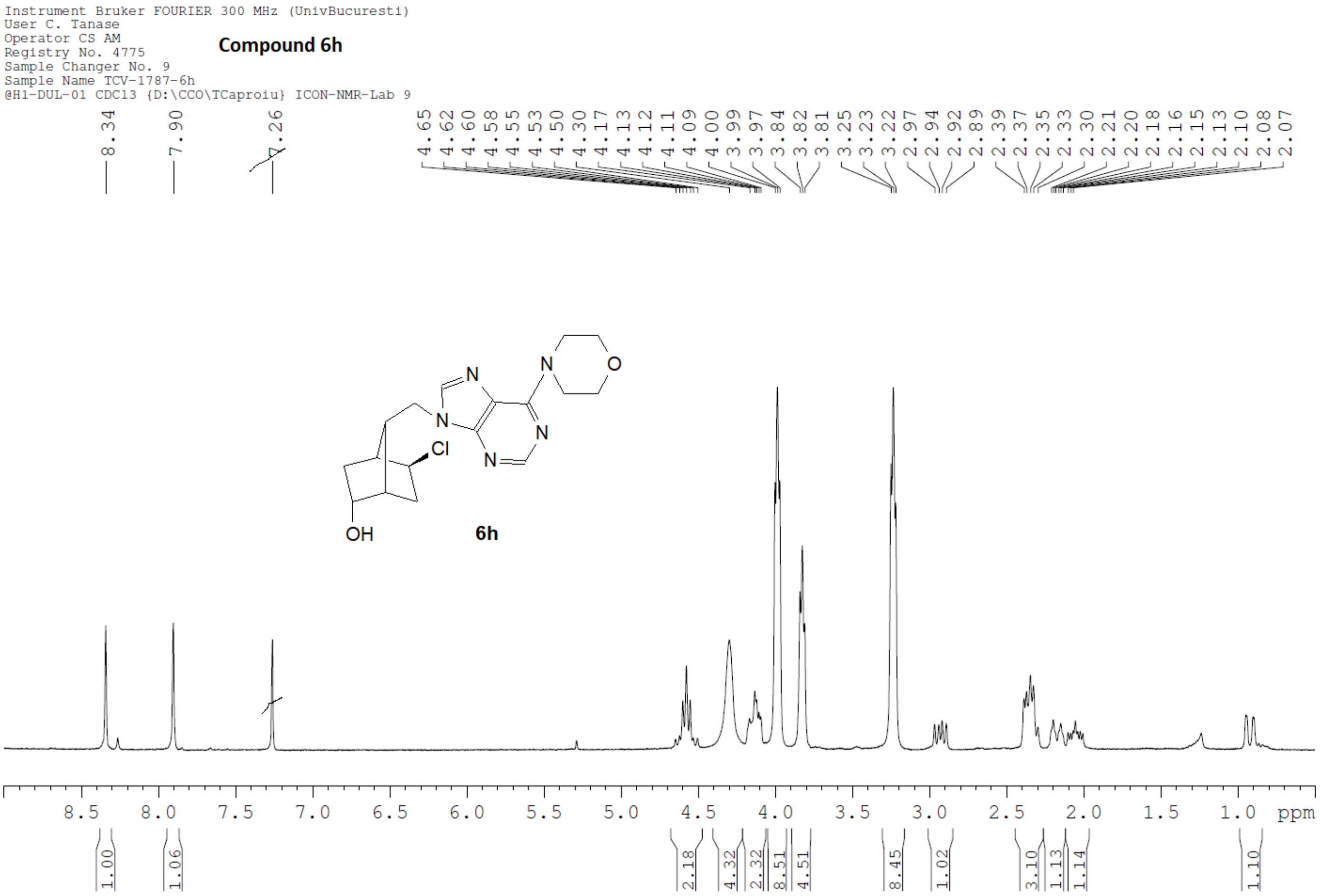

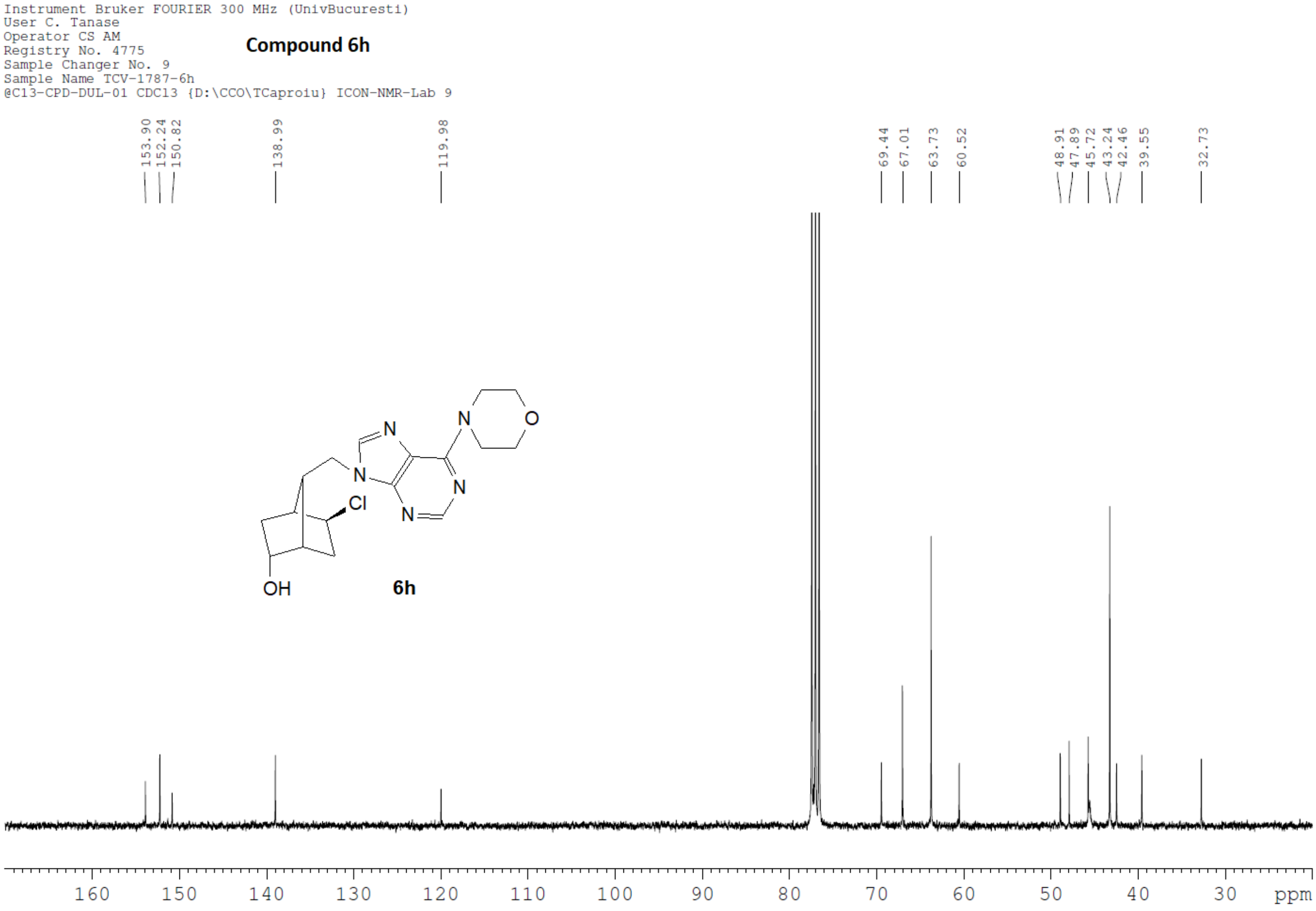

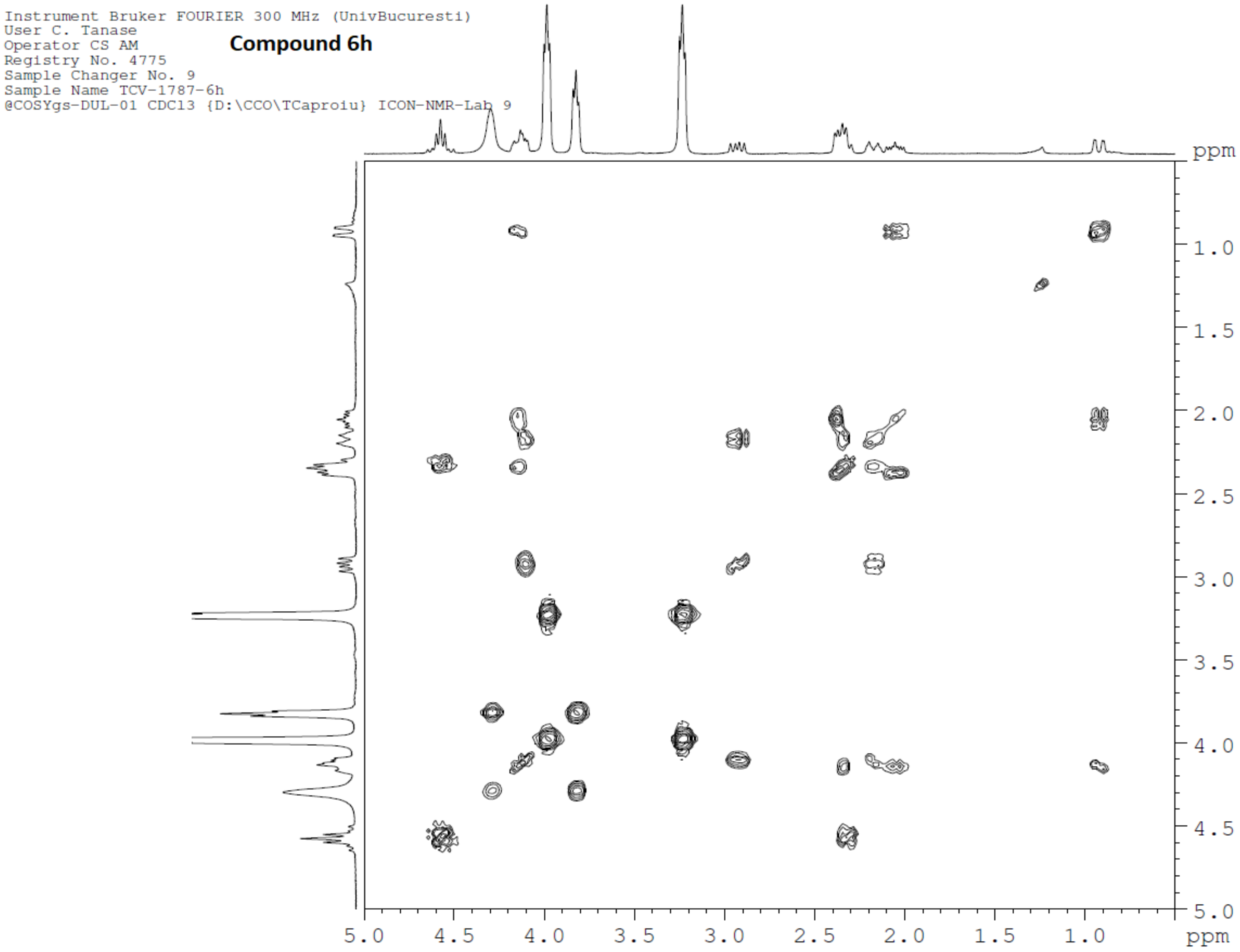
Instrument Bruker FOURIER 300 MHz (UnivBucuresti)
User C. Tanase
Operator CS AM
Registry No. 4775
Sample Changer No. 9
Sample Name TCV-1787-6h
@COSYgs-DUL-01 CDCl3 {D:\CCO\TCaproiu} ICON-NMR-Lab 9
Compound 6h
ppm

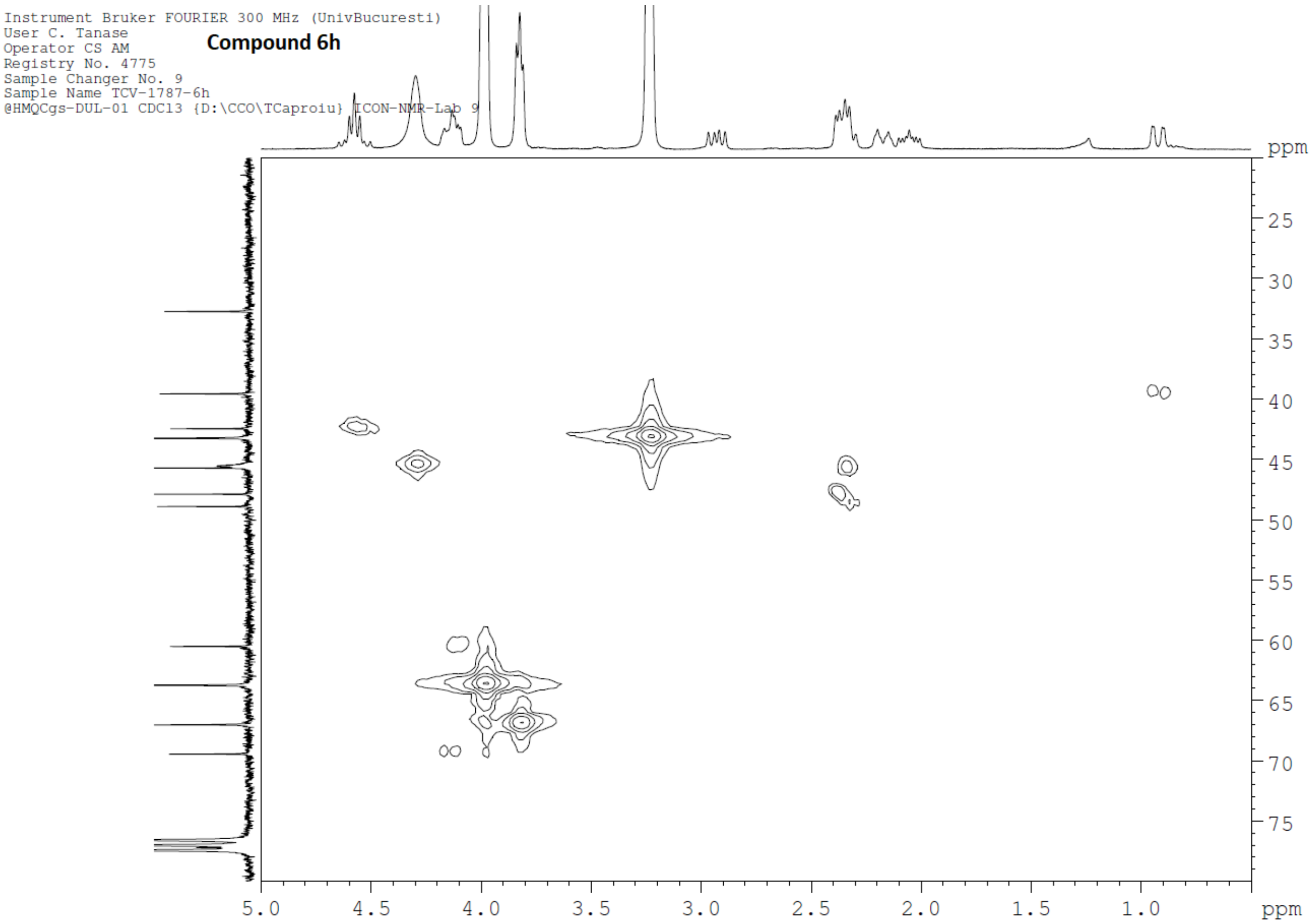
Instrument Bruker FOURIER 300 MHz (UnivBucuresti)
User C. Tanase
Operator CS AM
Registry No. 4775
Sample Changer No. 9
Sample Name TCV-1787-6h
@HMQCgs-DUL-01 CDCl3 {D:\CCO\TCaproiu} ICON-NMR-Lab 9
Compound 6h
ppm

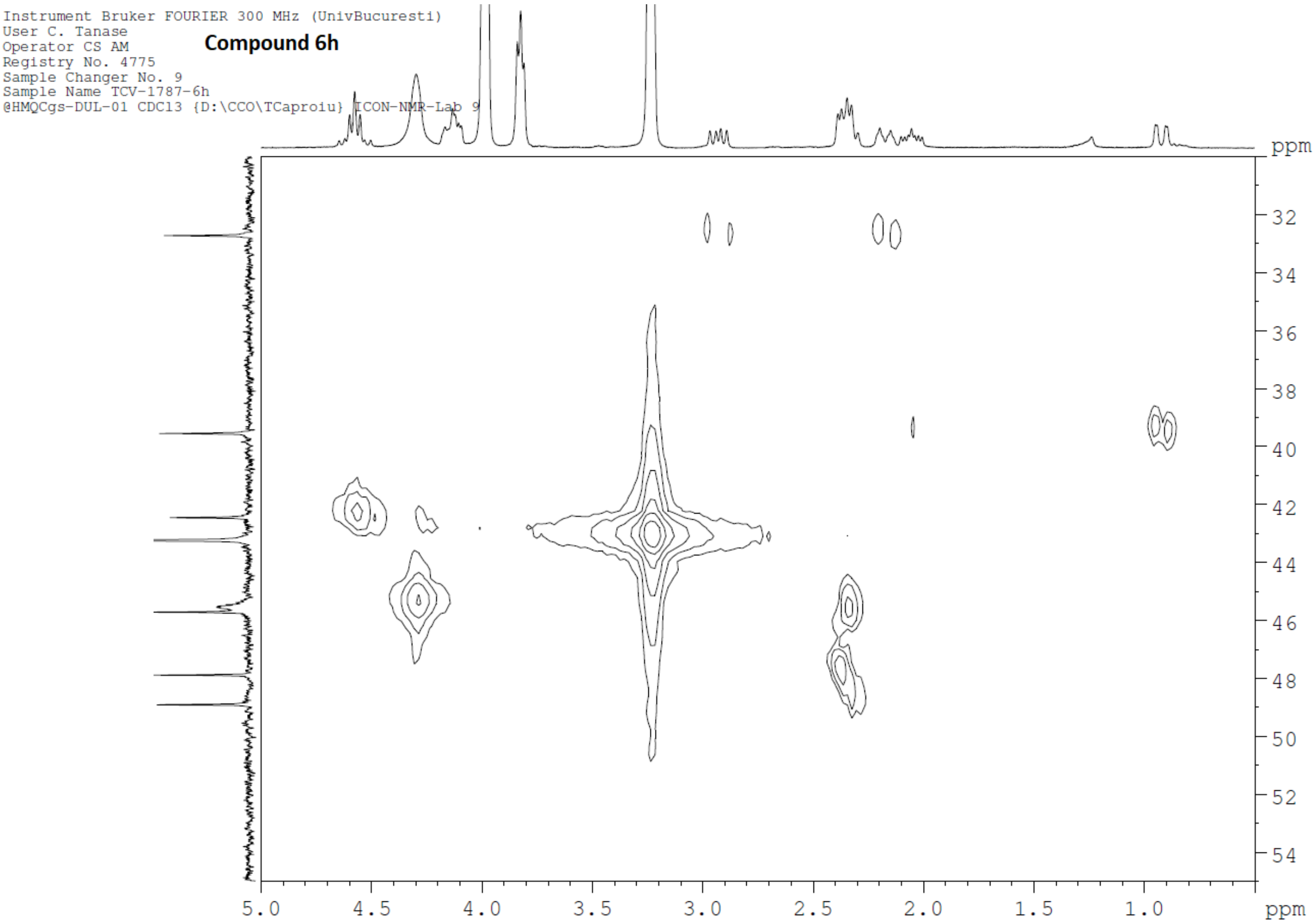

1.14. ^{1}H, ^{13}C, COSY and HETCOR (aliphatic)-NMR spectra in DMSO of the compound **6i**

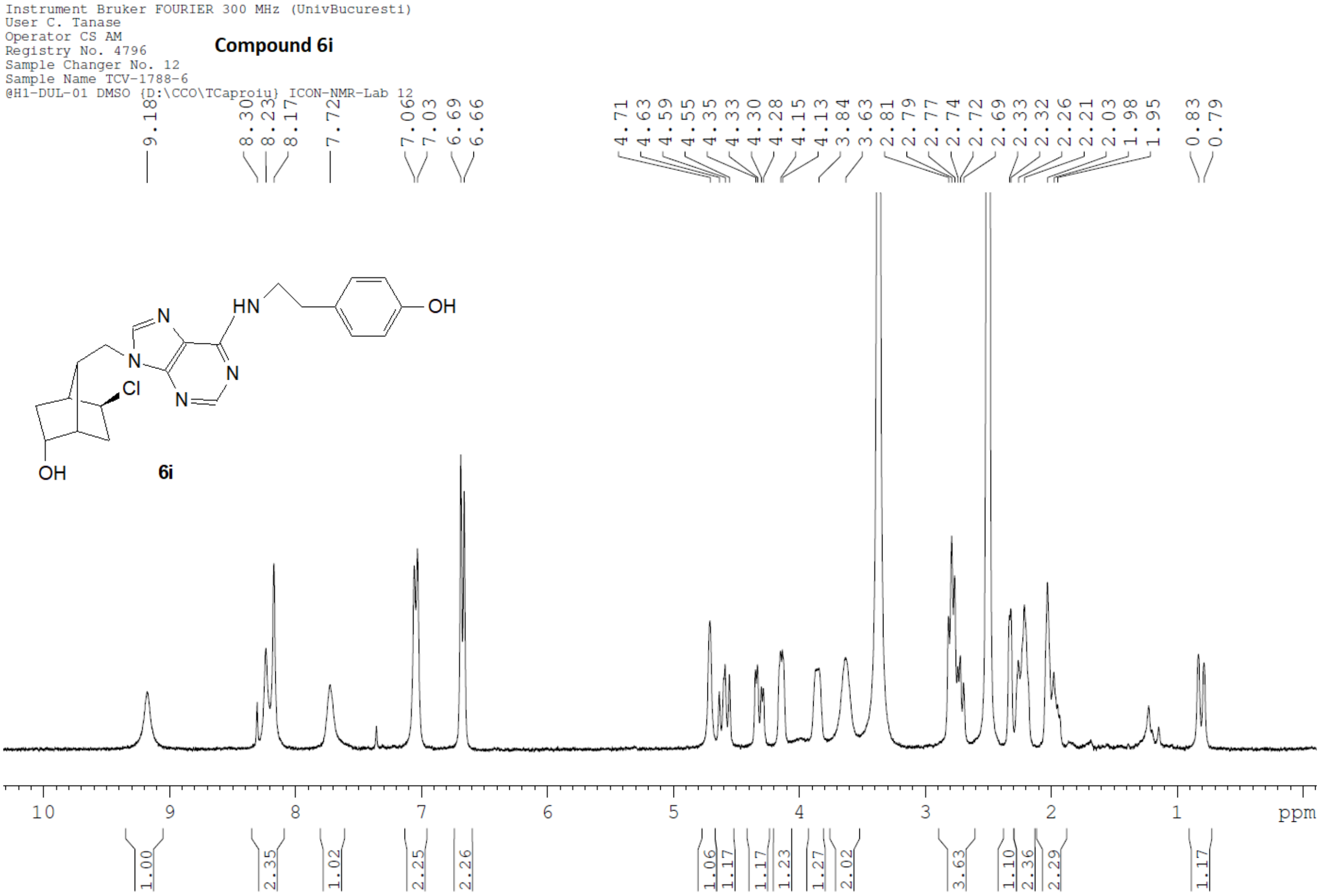

User C. Tanase
Operator CS AM
Registry No. 4796
Sample Changer No. 12
Sample Name TCV-1788-6
@C13-CPD-DUL-01 DMSO {D:\CCO\TCaproiu} ICON-NMR-Lab 12

Compound 6i

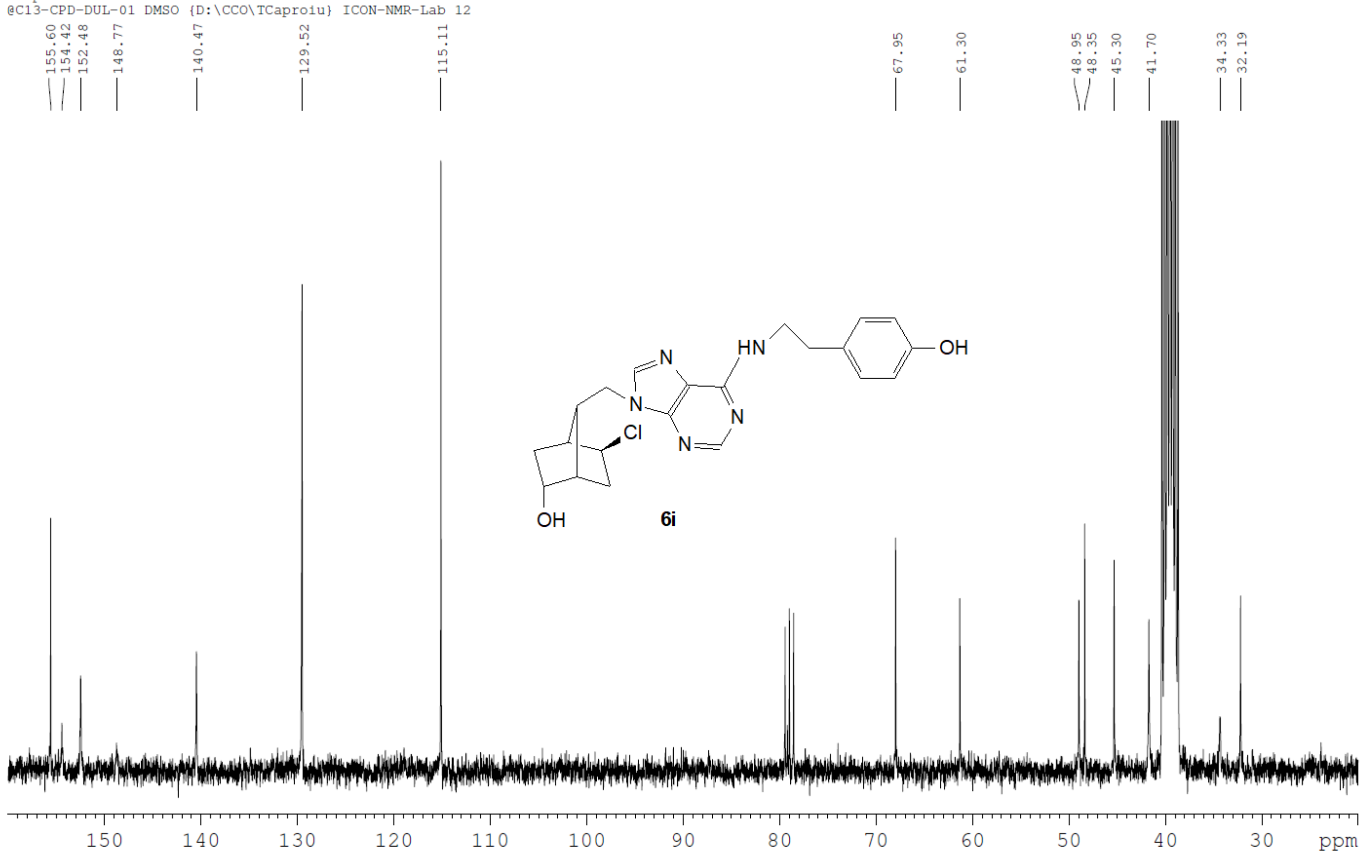

Instrument Bruker FOURIER 300 MHz (UnivBucuresti)
User C. Tanase
Operator CS AM
Registry No. 4796
Sample Changer No. 12
Sample Name TCV-1788-6
@COSYgs-DUL-01 DMSO {D:\CCO\TCaproiu} ICON-NMR-Lab 12

Compound 6i

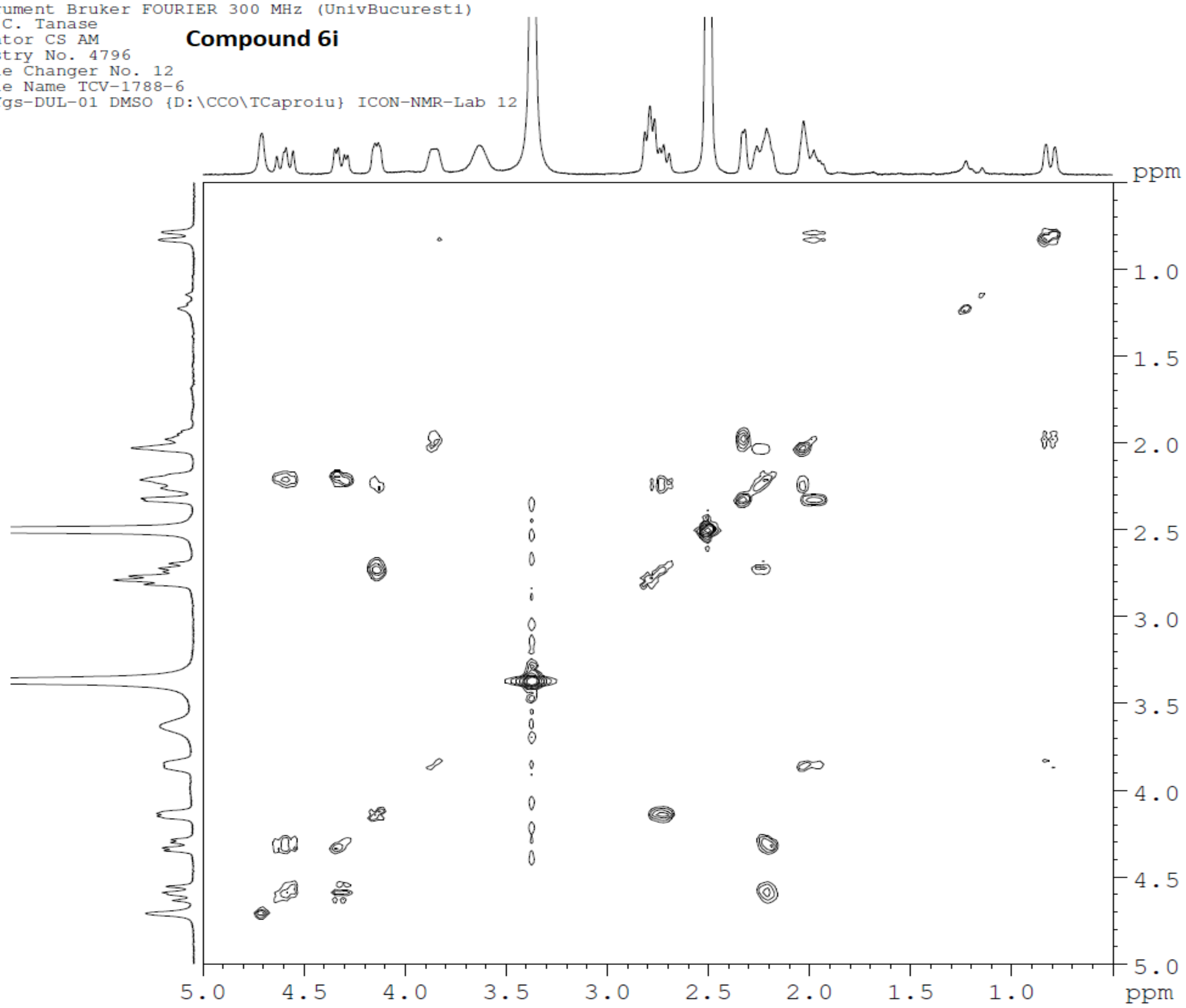

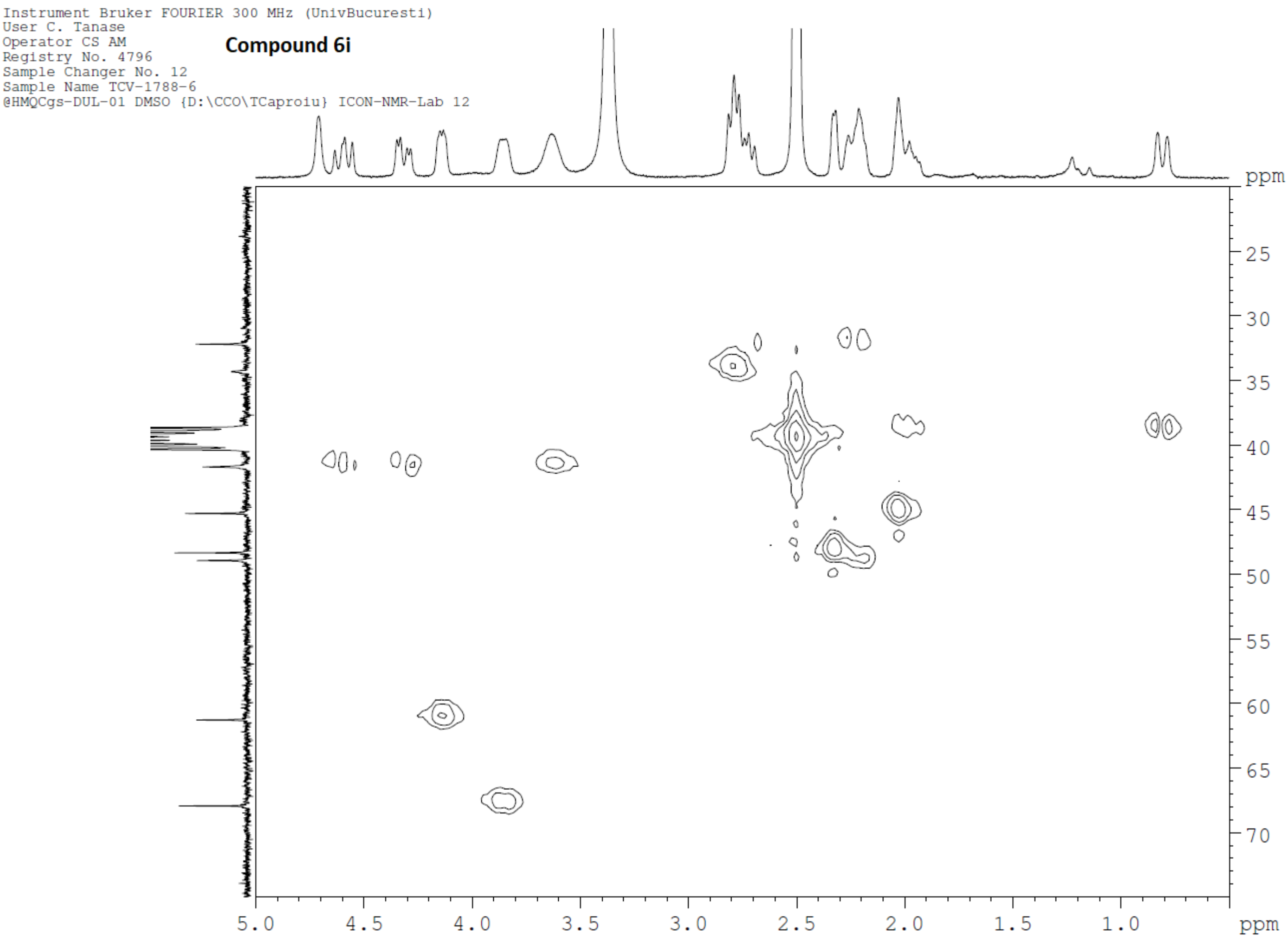

1.15. ^{1}H, ^{13}C, COSY and HETCOR (aliphatic)-NMR spectra in DMSO of the compound **6j**

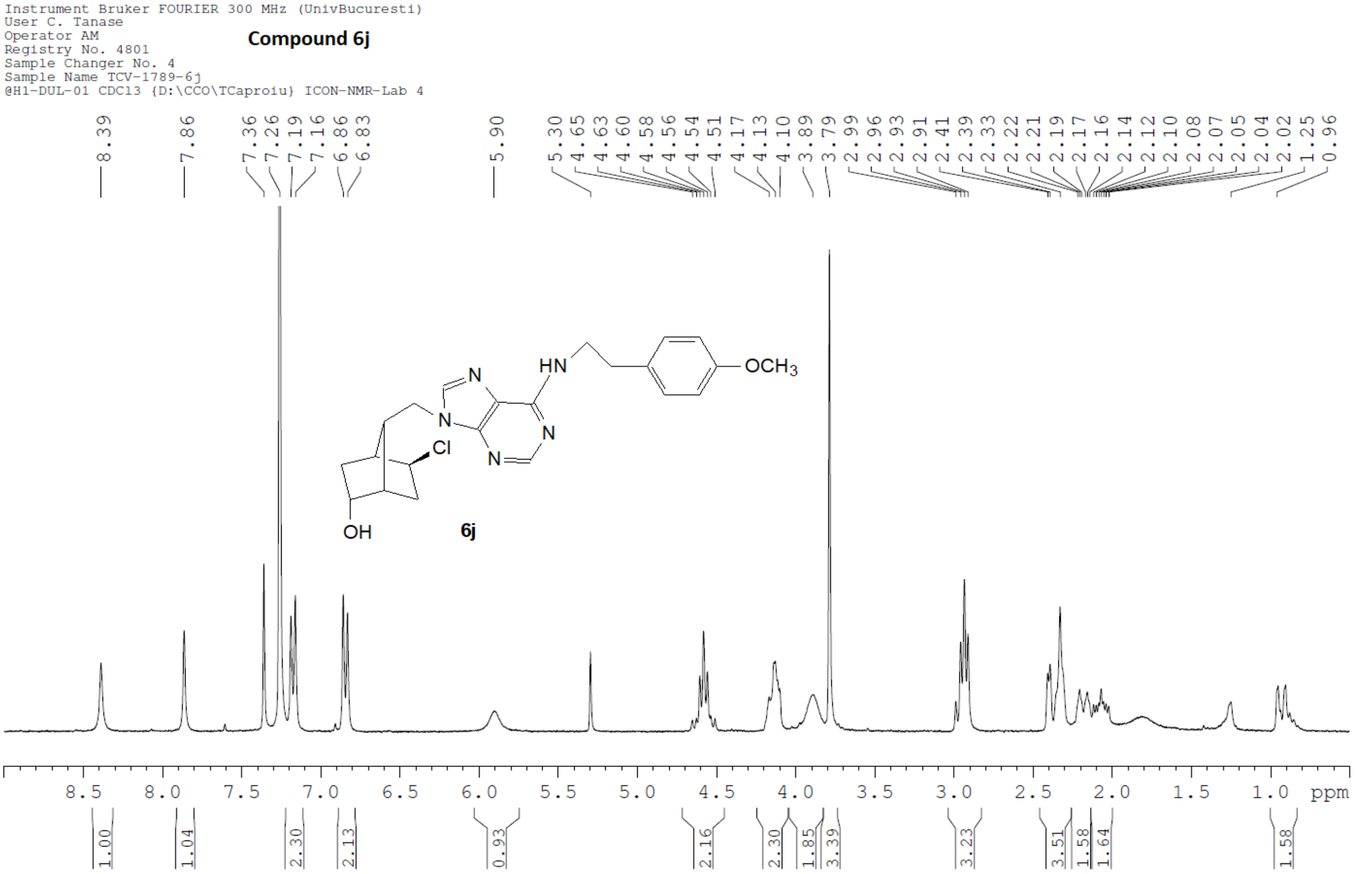

Instrument Bruker FOURIER 300 MHz (UnivBucuresti)
User C. Tanase
Operator AM
Registry No. 4801
Sample Changer No. 4
Sample Name TCV-1789-6j
@C13-CPD-DUL-01 CDCl3 {D:\CCO\TCaproiu} ICON-NMR-Lab 4

Compound 6j

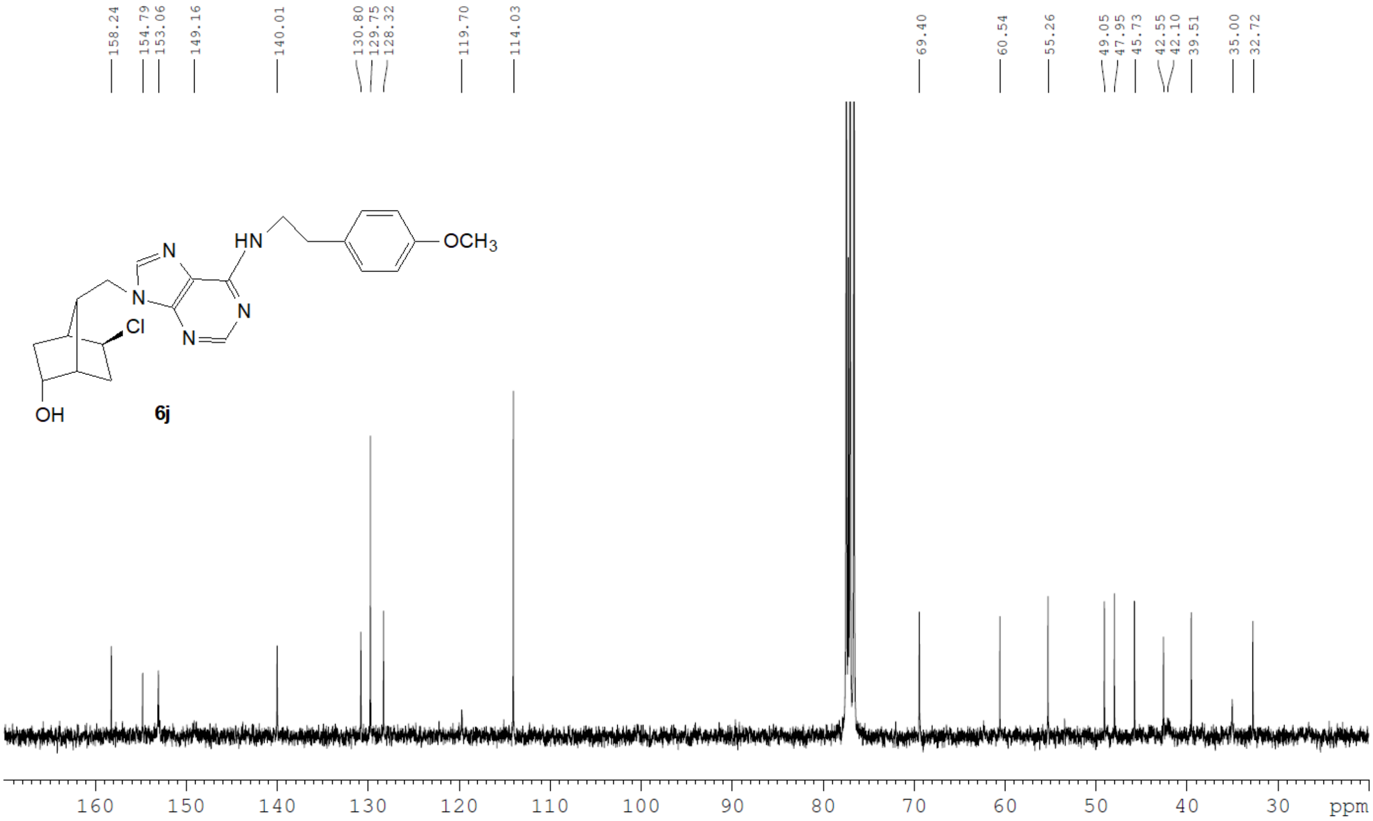

Instrument Bruker FOURIER 300 MHz (UnivBucuresti)
User C. Tanase
Operator AM
Registry No. 4801
Sample Changer No. 4
Sample Name TCV-1789-6j
@COSYgs-DUL-01 CDCl3 {D:\CCO\TCaproiu} ICON-NMR-Lab 4

Compound 6j

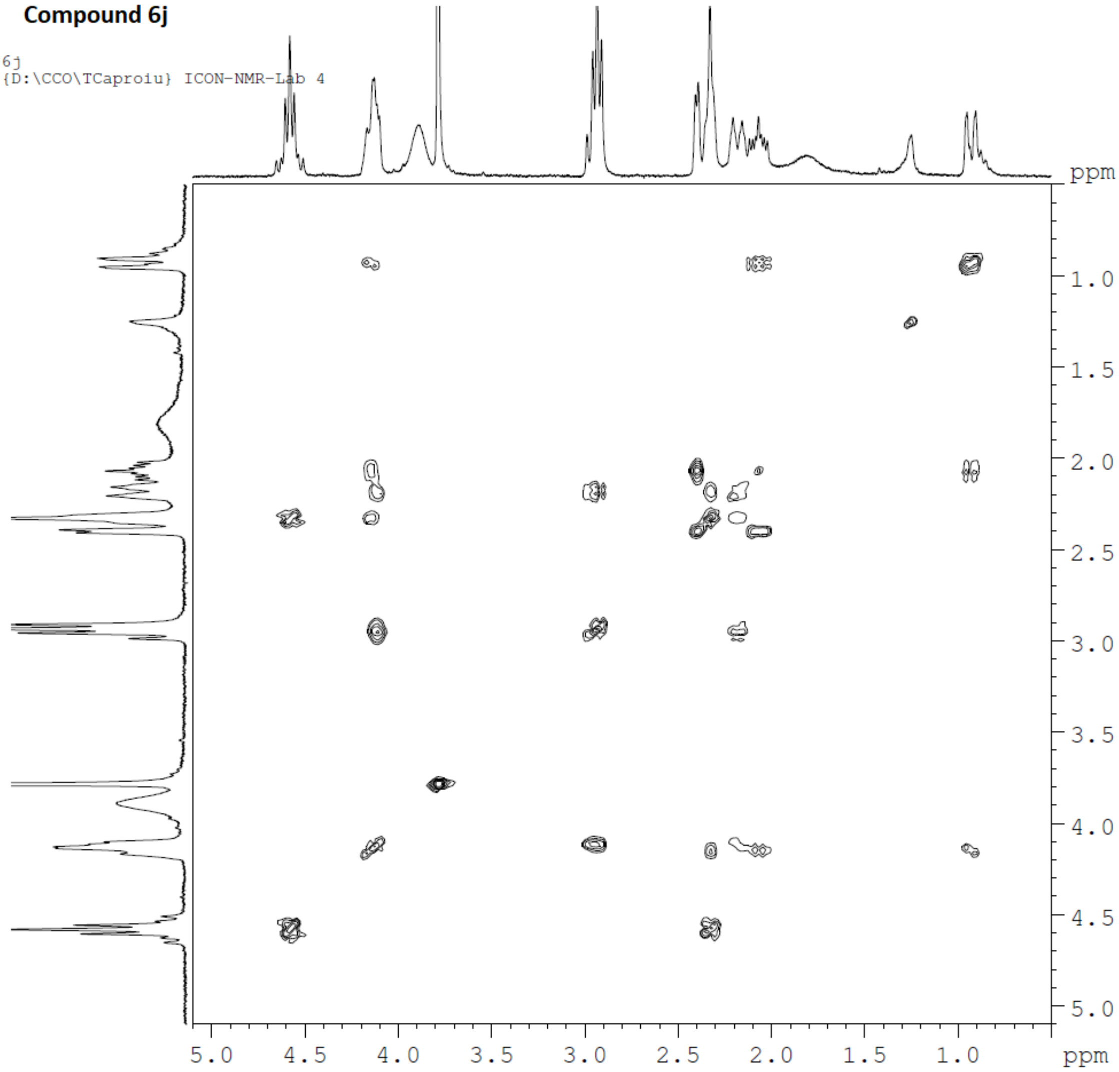

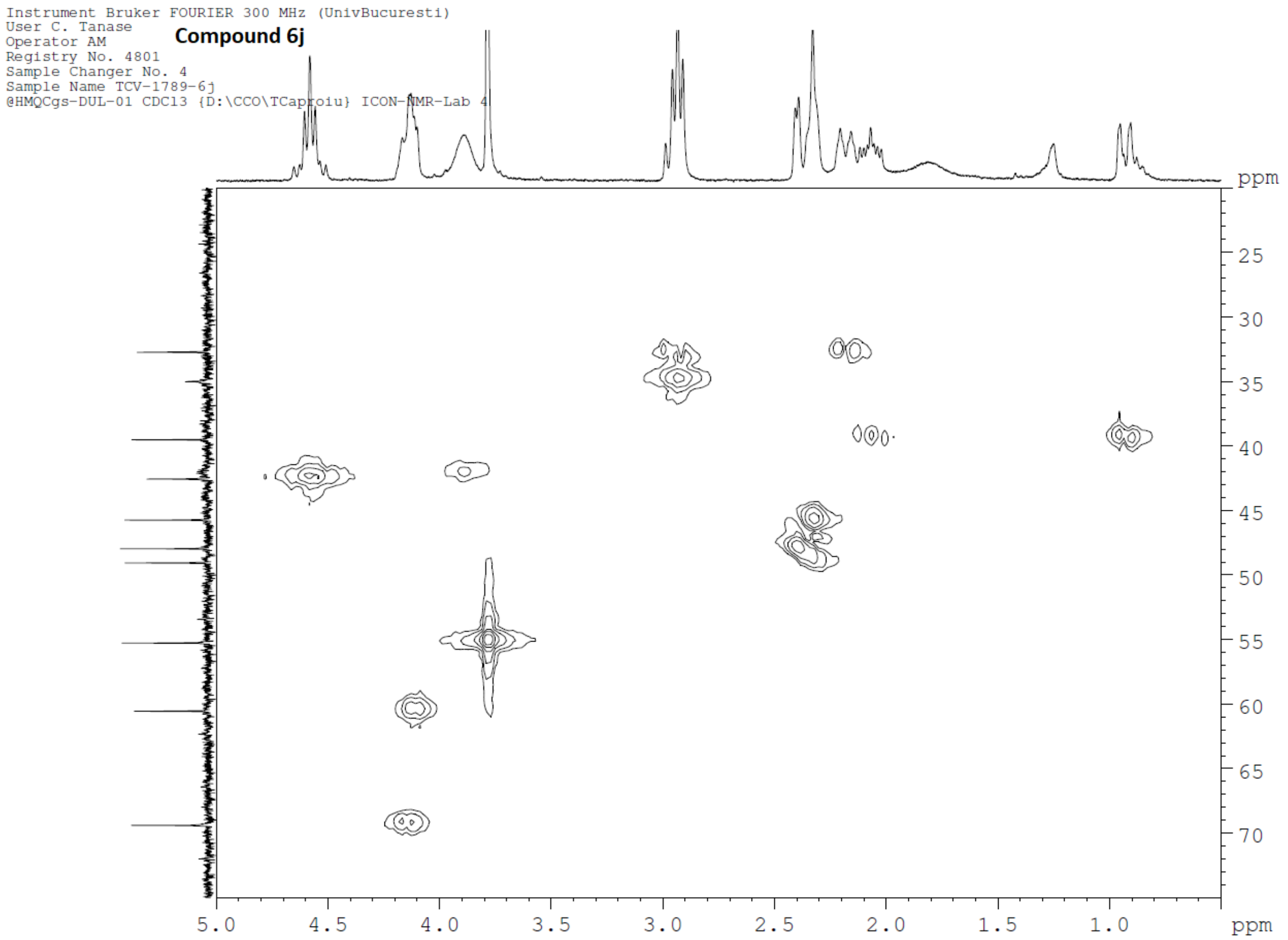

1.16. ^{1}H, ^{13}C, COSY and HETCOR (aliphatic)-NMR spectra in DMSO of the compound **6k**

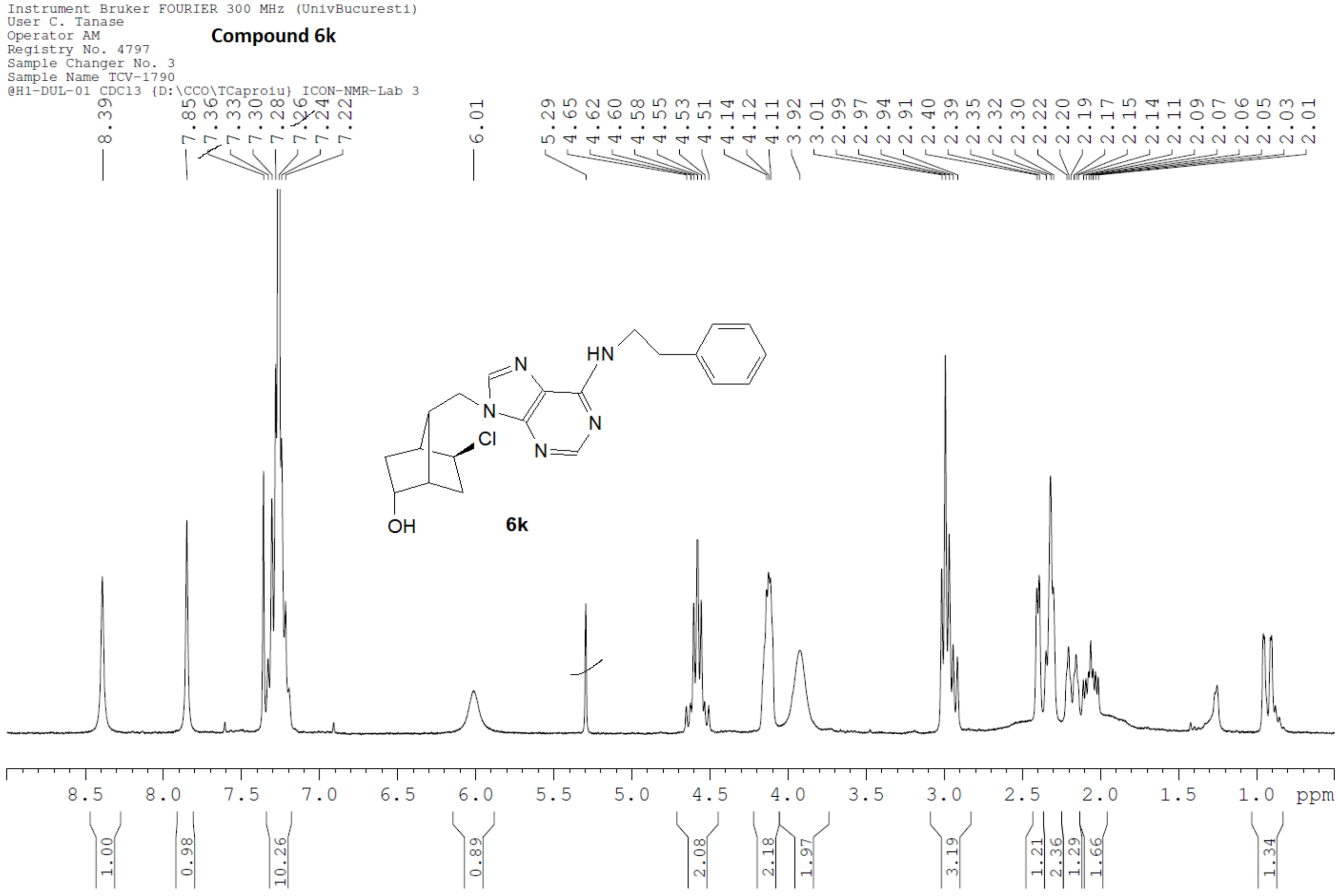

1.17. ^{1}H, ^{13}C, COSY and HETCOR (aliphatic)-NMR spectra in DMSO of the compound **7a**

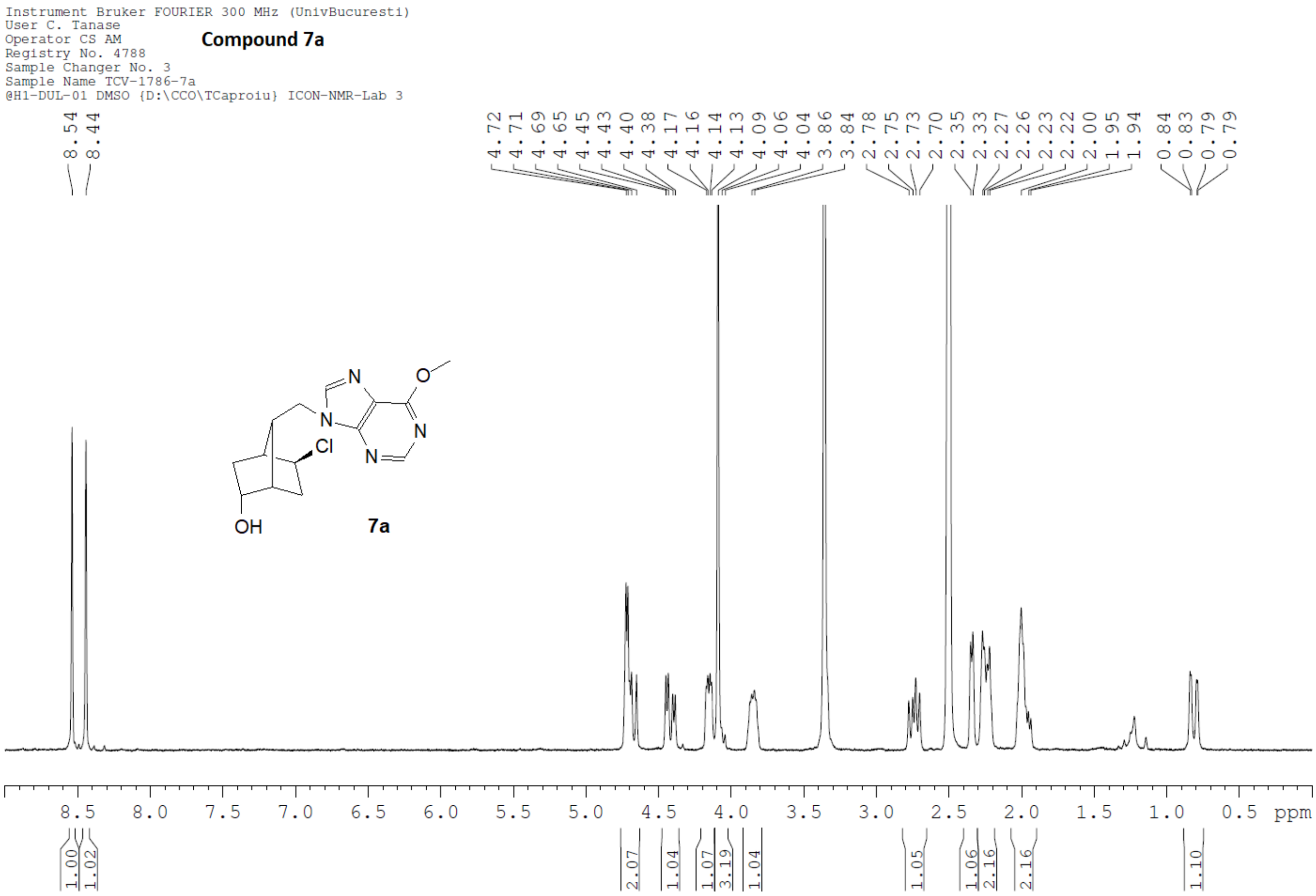

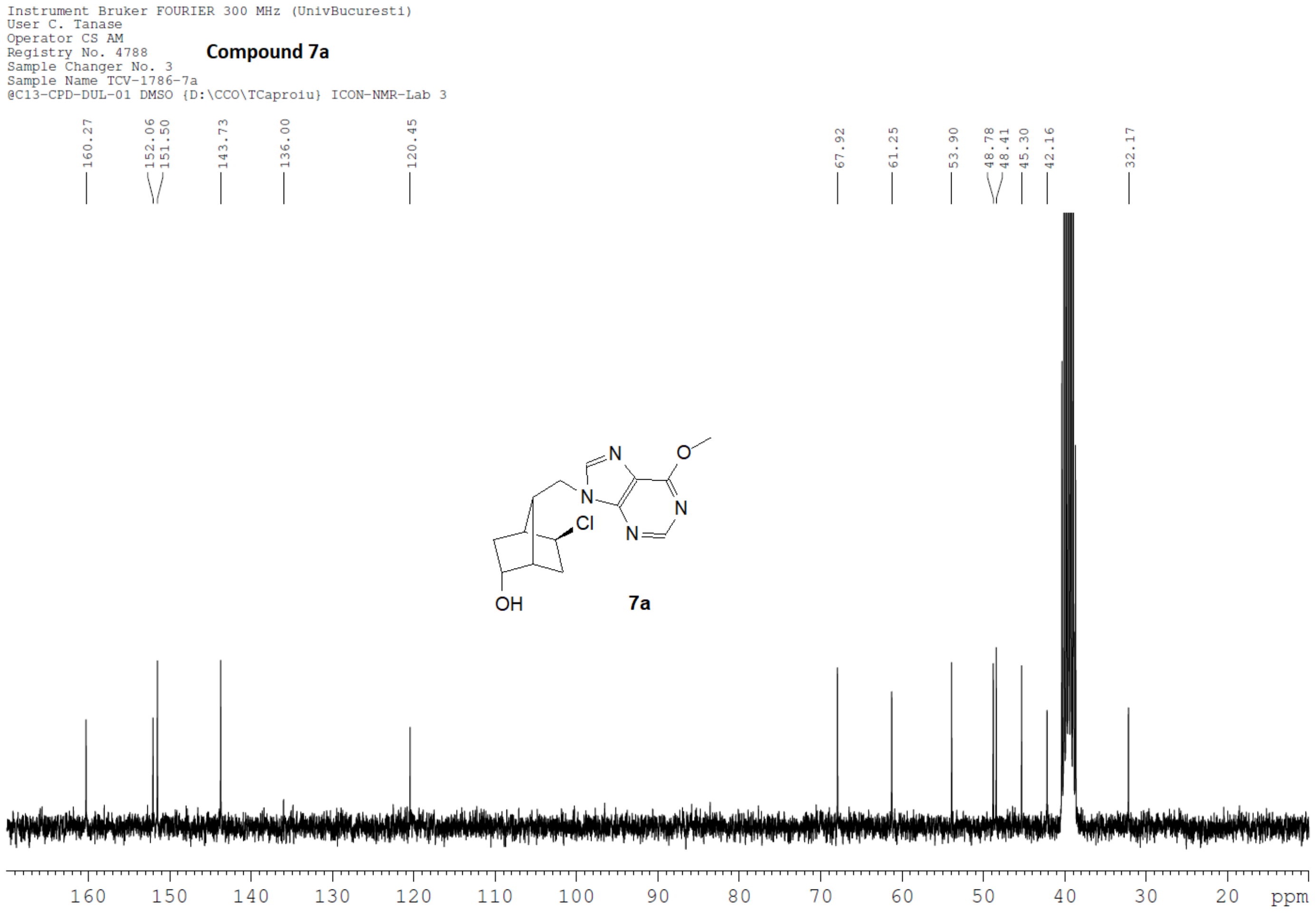

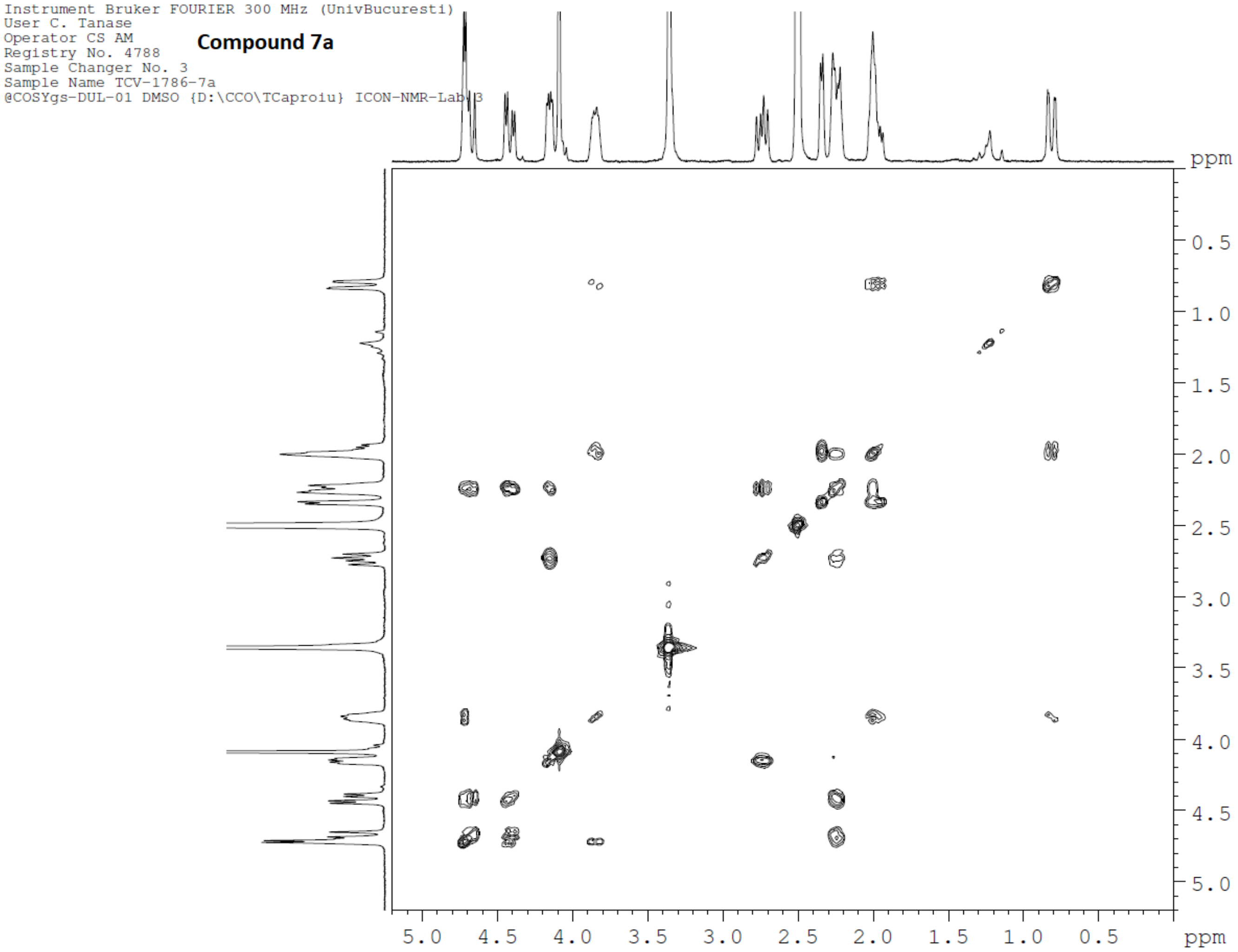
Instrument Bruker FOURIER 300 MHz (UnivBucuresti)
User C. Tanase
Operator CS AM
Compound 7a
Registry No. 4788
Sample Changer No. 3
Sample Name TCV-1786-7a
@COSYgs-DUL-01 DMSO {D:\CCO\TCaproiu} ICON-NMR-Lab 3
ppm
0.5
1.0
1.5
2.0
2.5
3.0
3.5
4.0
4.5
5.0
5.0 4.5 4.0 3.5 3.0 2.5 2.0 1.5 1.0 0.5
ppm

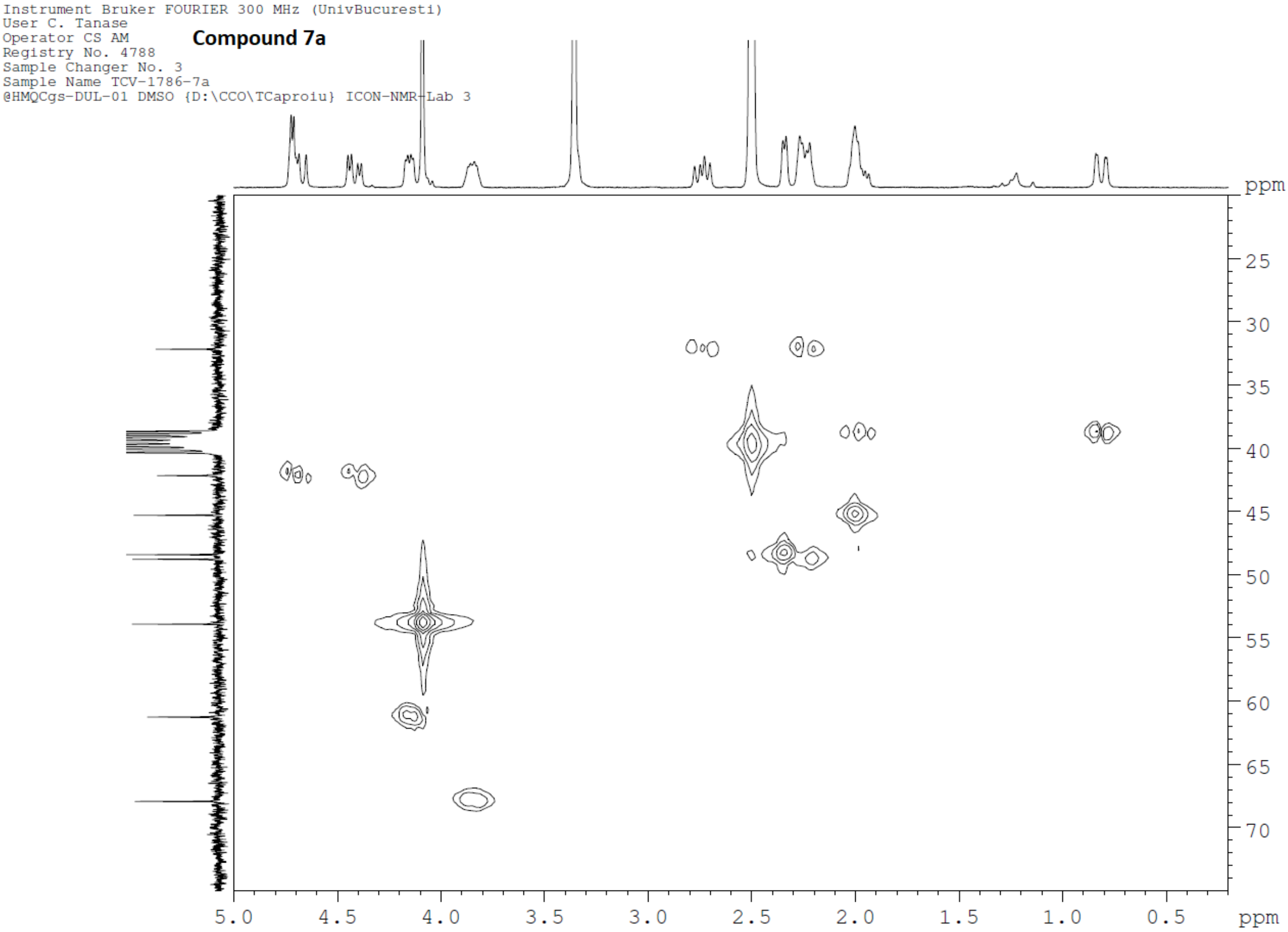
Instrument Bruker FOURIER 300 MHz (UnivBucuresti)
User C. Tanase
Operator CS AM
Compound 7a
Registry No. 4788
Sample Changer No. 3
Sample Name TCV-1786-7a
@HMQCgs-DUL-01 DMSO {D:\CCO\TCaproiu} ICON-NMR-Lab 3
ppm
25
30
35
40
45
50
55
60
65
70
5.0 4.5 4.0 3.5 3.0 2.5 2.0 1.5 1.0 0.5
ppm

1.18. ^{1}H, ^{13}C, COSY and HETCOR (aliphatic)-NMR spectra in DMSO of the compound **7b**

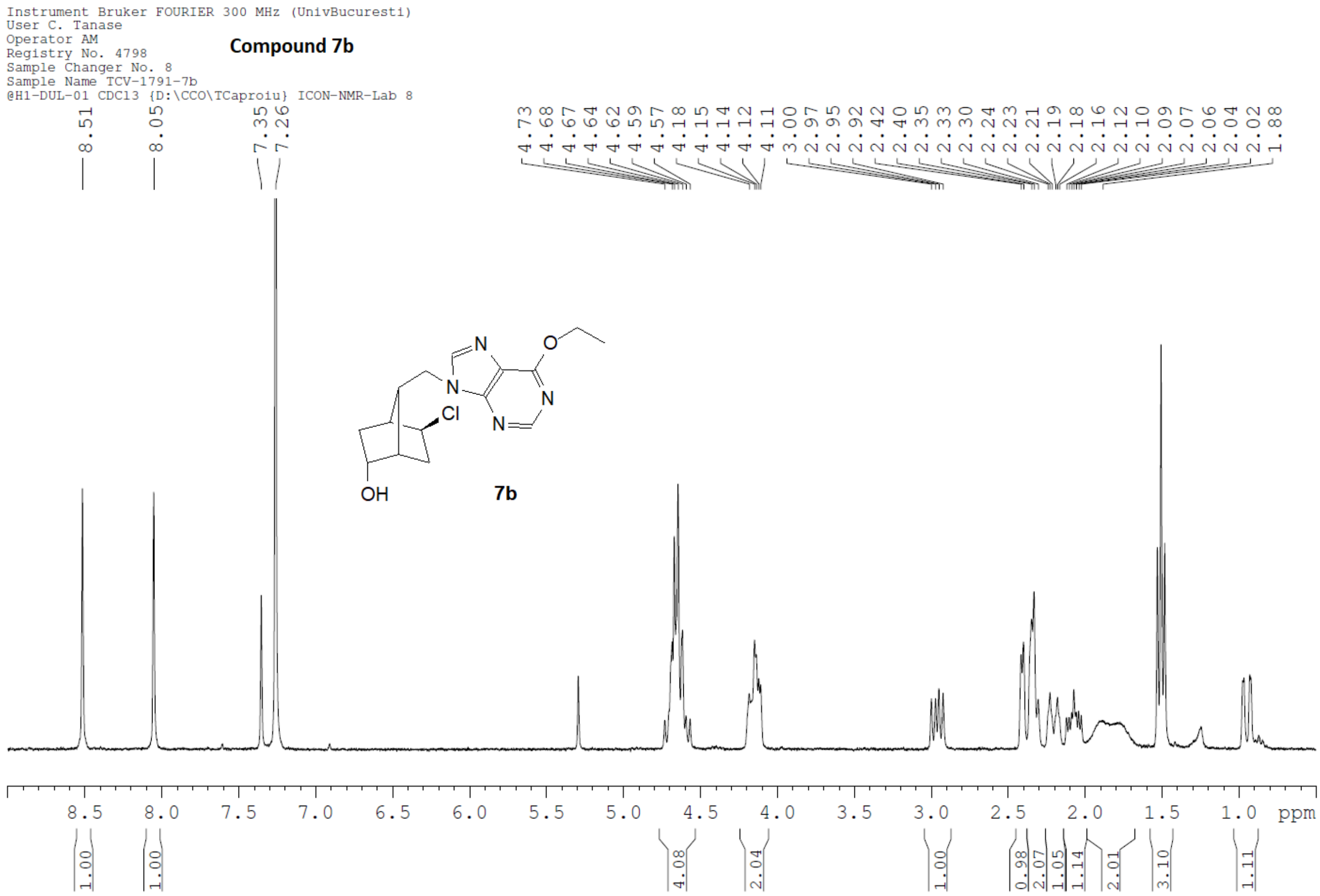

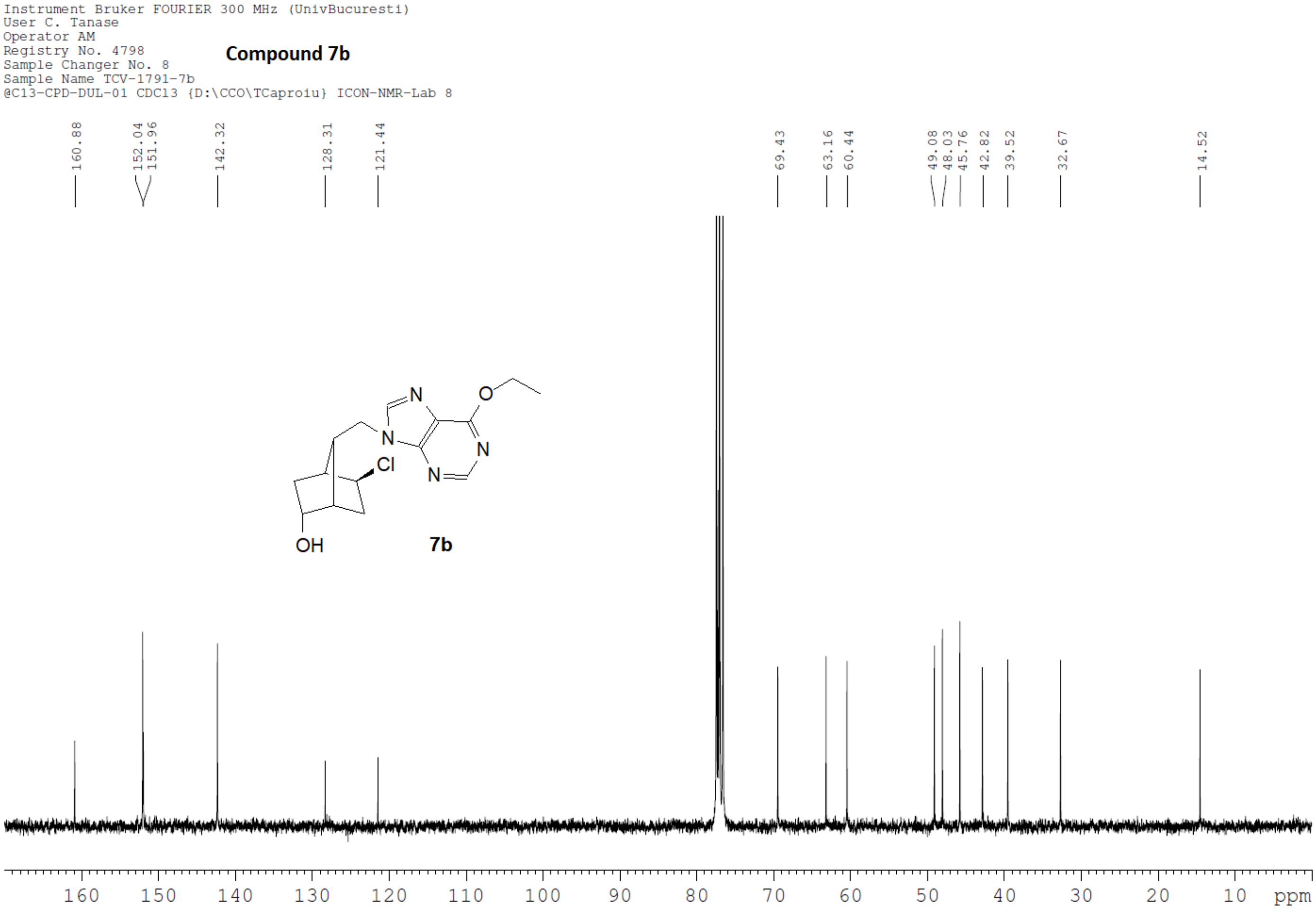

Instrument Bruker FOURIER 300 MHz (UnivBucuresti)
User C. Tanase
Operator AM
Registry No. 4798
Sample Changer No. 8
Sample Name TCV-1791-7b
@COSYgs-DUL-01 CDCl3 {D:\CCO\TCaproiu} ICON-NMR-Lab 8

Compound 7b

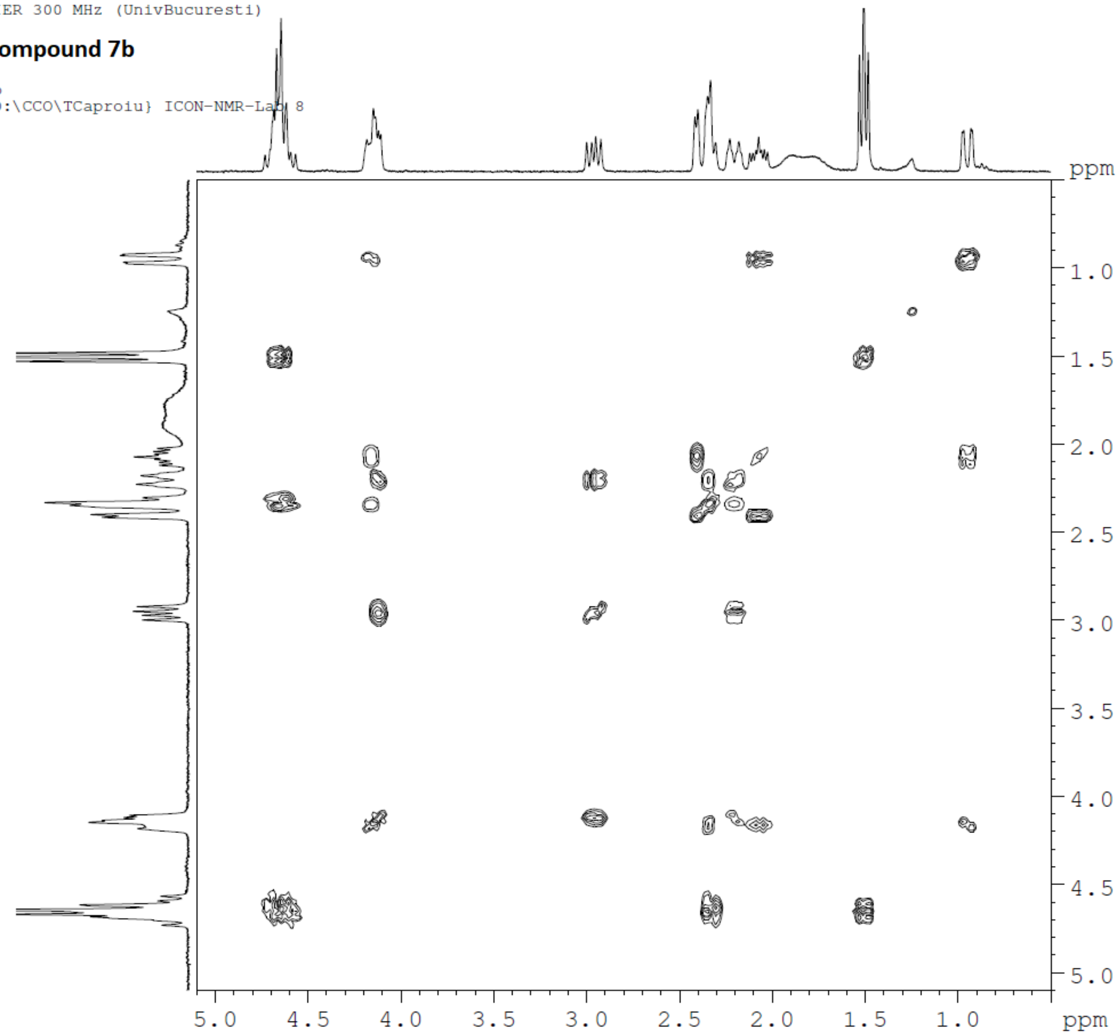

Instrument Bruker FOURIER 300 MHz (UnivBucuresti)
User C. Tanase
Operator AM
Registry No. 4798
Sample Changer No. 8
Sample Name TCV-1791-7b
@HMQCgs-DUL-01 CDCl3 {D:\CCO\TCaproiu} ICON-NMR-Lab 8

Compound7b

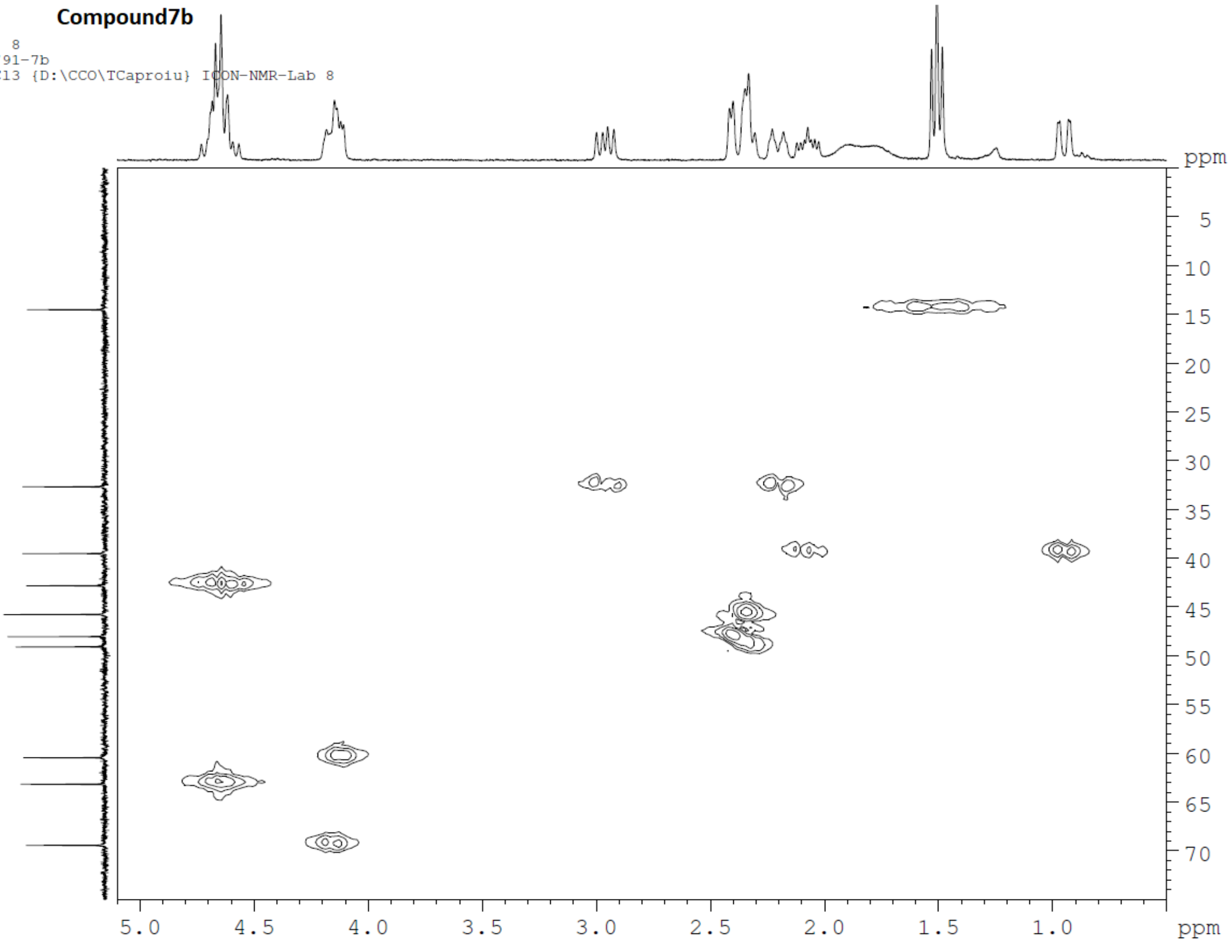

Docking studies

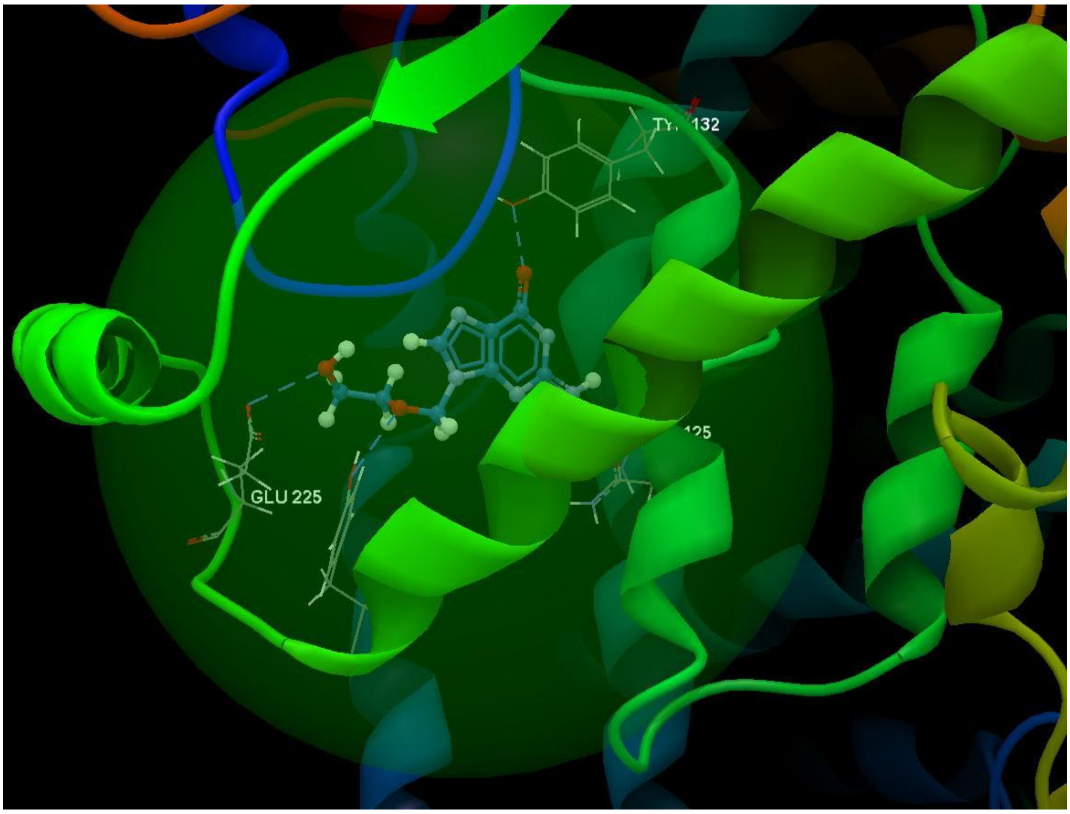

(a)

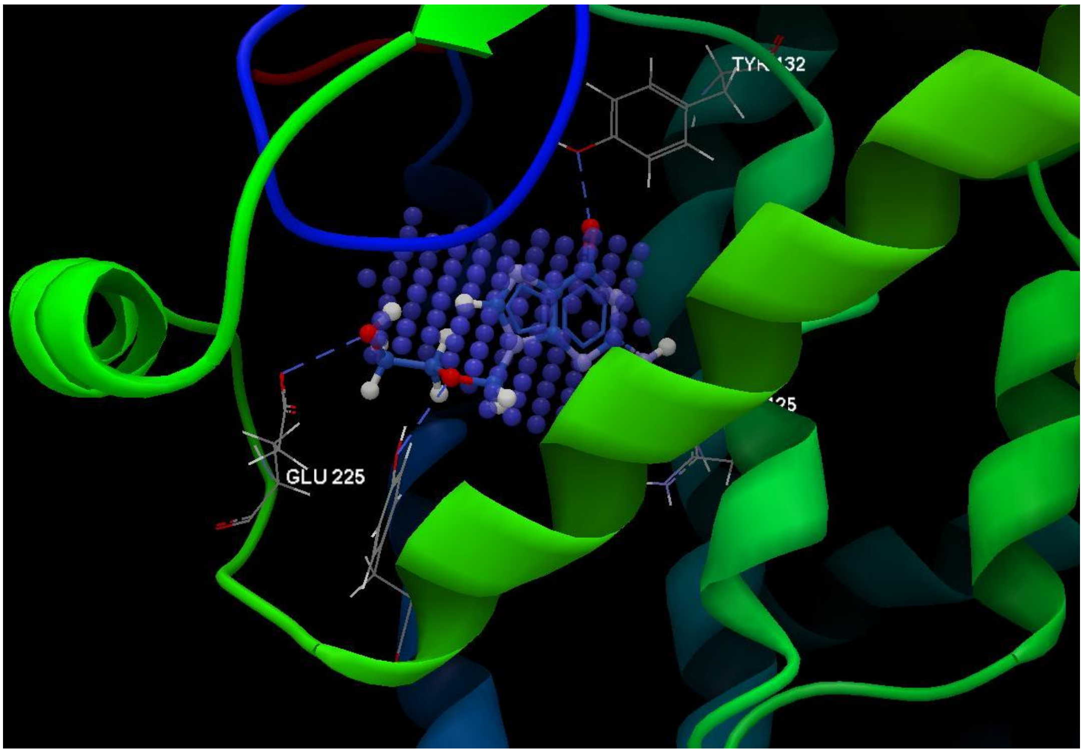

(b)

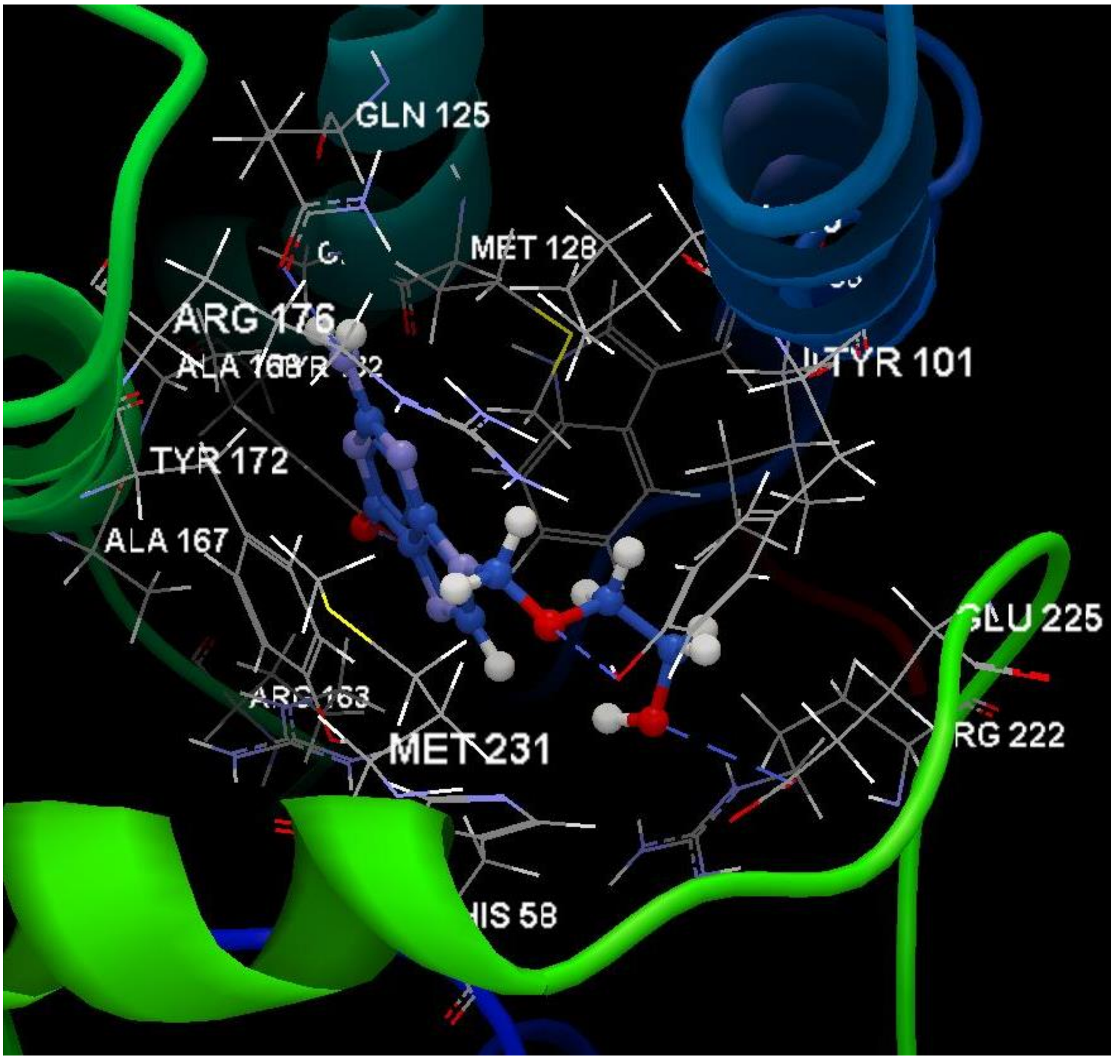

(c)

Figure S1. (a) Binding site and docking pose of the co-crystallized AC2 (hydrogen bonds are in blue dashlines); (b) Binding pocket and docking pose of the co-crystallized AC2 (hydrogen bonds are in blue dashlines); (c) Docking pose of the co-crystallized AC2 interacting with the amino acid residues of the ligand binding site of thymidine kinase

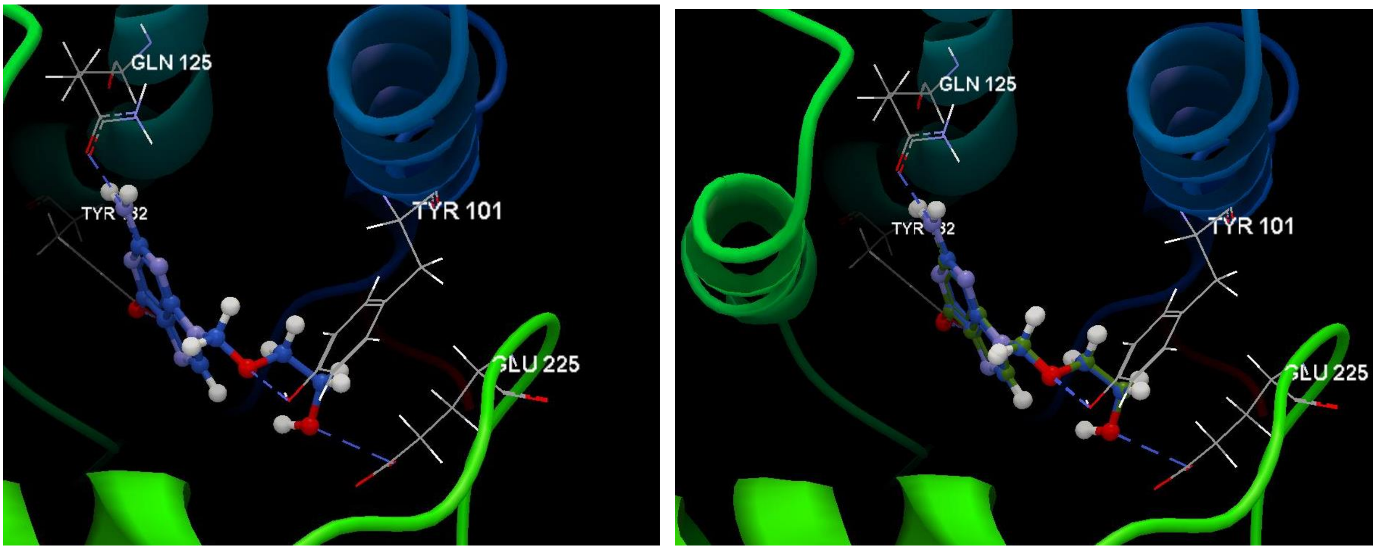

(a) (b)

Figure S2. (a) Hydrogen bonds between amino acids residues and co-crystallized AC2; (b) Docking validation of the co-crystallized;

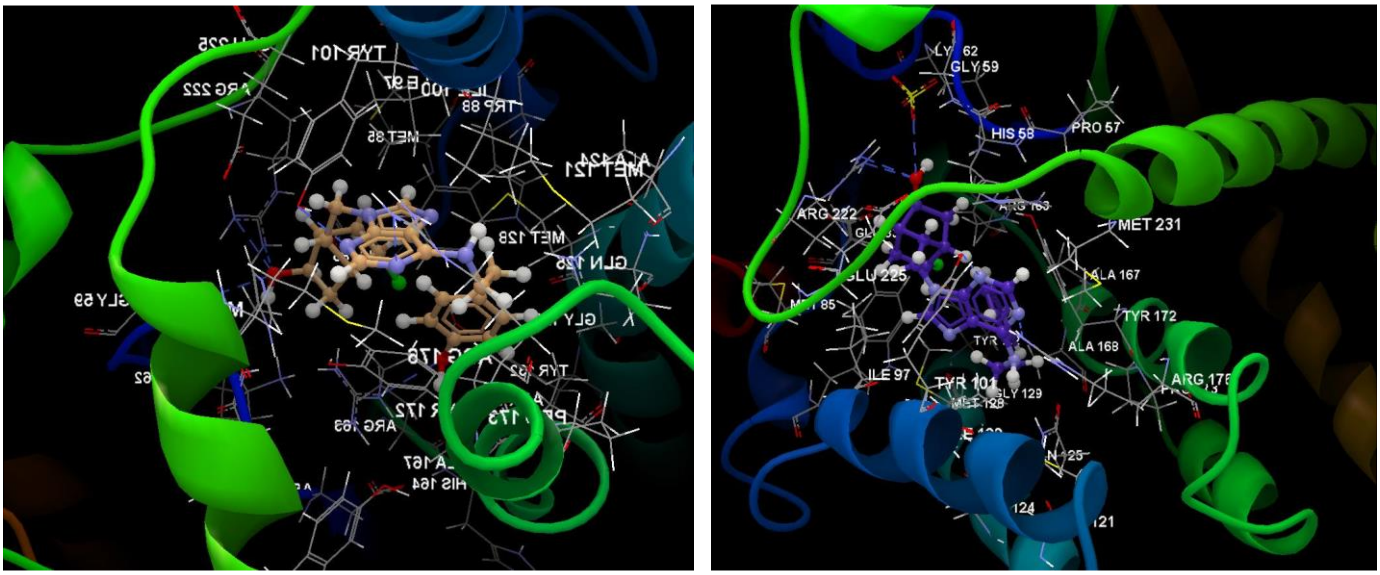

(a) (b)

Figure S3. (a) Docking pose of the **6i** ligand interacting with amino acids residues; (b) Docking pose of the **6k** ligand interacting with amino acids residues.

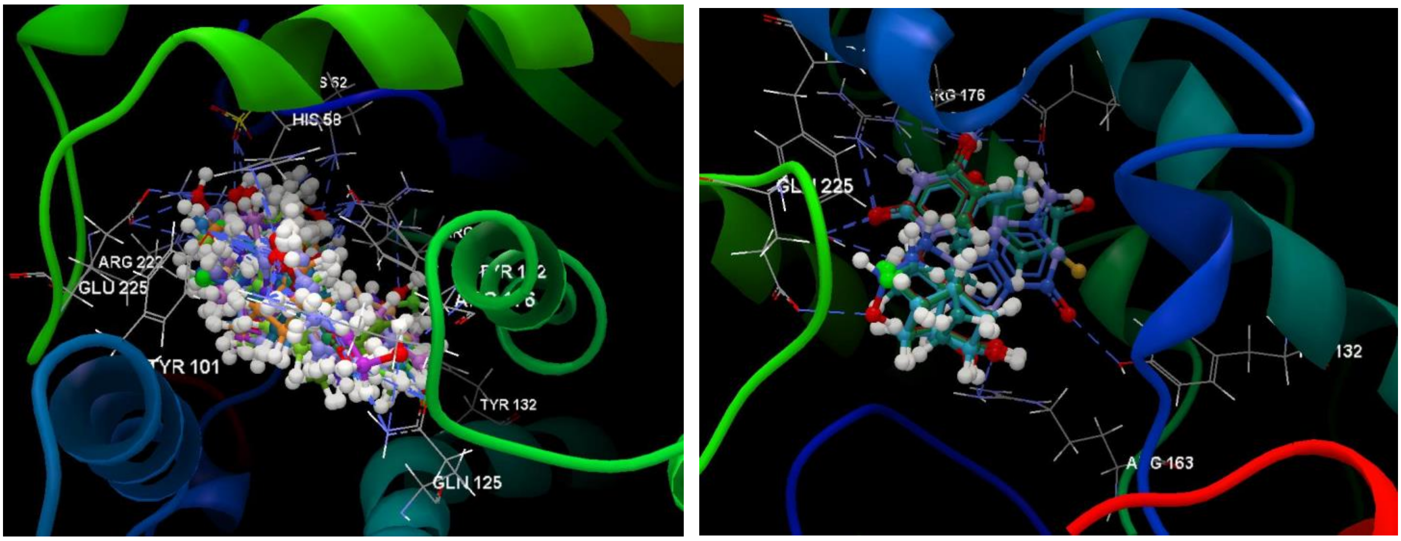

(a) (b)

Figure S4. (a) Docking pose of all ligands (b) Docking poses of AC2 and of the compound **4a-4d**.

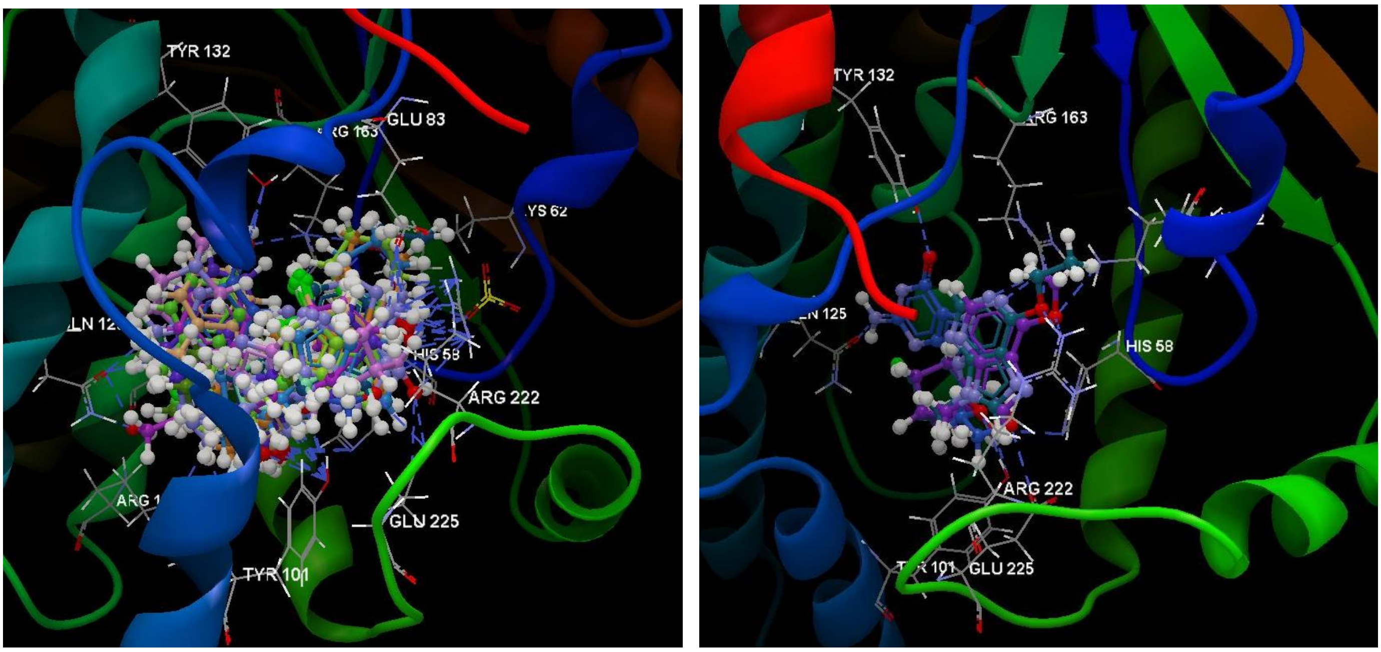

(a) (b)

Figure S5. (a) Docking poses of AC2 and of the compound **6a**-**6k**,
(b) Docking poses of AC2 and of the compound **7a**,**7b**

Table S1. List of docking interactions between the ligand molecules and thymidine kinase using CLC Drug Discovery Workbench Software.

Ligand	Score	RMSD	Group interaction	Hydrogen bond	Bond length
**Acyclovir**	-49.29	0.71	GLN 125; GLY 129; ILE 100; MET 128; TRP 88; ILE 97; TYR 101; GLU 225; ARG 222; ARG 176; ALA 168; TYR 132; TYR 172; ALA 167; ARG 163; MET 231; HIS 58	- N sp^2 (N2)– O sp^2 from GLN 125<br>- O sp^2 (O6) – O sp^3 from TYR 132<br>- O sp^3 (O3) – O sp^2 from GLU 225<br>- O sp^3 (O1) – O sp^3 from TYR 101	2.728 Å<br>2.905 Å<br>3.023 Å<br>3.108 Å
**4a**	-63.16	0.01	GLN 125; GLY 129; ILE 100; MET 128; TRP 88; ILE 97; TYR 101; GLU 225; ARG 222; ARG 176; ALA 168; TYR 132; TYR 172; ALA 167; ARG 163; MET 231; HIS 58; PRO 173; LYS 62; GLU 83;	- N sp^2 ($N3^1$)– O sp^2 from GLN 125<br>- O sp^3 (O5) – N sp^2 from ARG 163	2.664 Å<br>3.057 Å
**4b**	-59.49	0.008	MET 231; GLU 225; TYR 101; ARG 222; HIS 58; LYS 62; ARG 163; ARG 176; ILE 100; ILE 97; MET 121; TYR 172; GLU 83; TYR 132; MET 128; TRP 88; GLN 125.	- O sp^2 ($O4^1$) – N sp^2 from ARG 176<br>- O sp^2 ($O4^1$) – N sp^2 from GLN 125<br>- O sp^2 ($O2^1$) – N sp^2 from ARG 176<br>- O sp^2 ($O2^1$) – O sp^3 from TYR 101<br>- O sp^3 (O5) – N sp^2 from ARG 163	3.224 Å<br>2.883 Å<br>3.145 Å<br>2.753 Å<br>2.789 Å
**4c**	-62.37	0.02	TYR 132; GLU 83; ARG 163; ALA 168; TRP 88; MET 128; GLN 125; LYS 62; TYR 172; ILE 97; MET 121; ILE 100; ARG 176; TYR 101; GLU 225; MET 231; ARG 222; HIS 58;	- O sp^3 (O5) – N sp^2 from ARG 163<br>- O sp^2 ($O4^1$) – N sp^2 from GLN 125<br>- O sp^2 ($O2^1$) – O sp^3 from TYR 101<br>- O sp^2 ($O2^1$) – N sp^2 from ARG 176	2.833 Å<br>2.882 Å<br>2.652 Å<br>3.093 Å
**4d**	-65.53	0.01	MET 121; GLN 125; ILE 100; TYR 101; TRP 88; ILE 97; MET 128; ARG 176; PRO 173; TYR 172; MET 231; ARG 163; HIS 58; LYS 62; GLU 83; ARG 222; GLU 225; TYR 132.	- O sp^3 (O5) – N sp^2 from ARG 163<br>- O sp^2 ($O2^1$) – N sp^2 from ARG 176<br>- O sp^2 ($O2^1$) – O sp^3 from TYR 101<br>- N sp^2 ($N3^1$) – N sp^2 from ARG 176<br>- N sp^2 ($N3^1$) – N sp^2 from ARG 176<br>- N sp^3 ($N4^1$) – N sp^2 from ARG 176<br>- N sp^3 ($N4^1$)– N sp^2 from GLN 125<br>- N sp^3 ($N4^1$)– O sp^2 from GLN 125	2.779 Å<br>3.020 Å<br>2.862 Å<br>2.958 Å<br>3.078 Å<br>3.293 Å<br>2.794 Å<br>3.053 Å
**5**	-58.79	0.03	LYS 62; HIS 58; ARG 222; GLU 225; TYR 101; ILE 100; ARG 176; ILE 97; TYR 172; ARG 163; ALA 167; ALA 168; GLU 83; GLN 125; MET 128; TRP 88; GLY 129; TYR 132.	Ligand does not form hydrogen bonds with amino acid residues	
**6a**	-59.37	0.02	GLU 225; TYR 101; MET 231; ARG 222; GLY 59; HIS 58; PRO 57; LYS 62; ILE 97; ILE 100; ARG 176; MET 128; GLN 125; TRP 88; TYR 172; GLU 83; ASP 163; ARG 163; TYR 132.	- N sp^3 ($N6^1$) – N sp^2 from ARG 222<br>- N sp^3 ($N6^1$) – O sp^2 from SO4 A<br>- N sp^2 ($N1^1$) – N sp^2 from ARG 163<br>- O sp^3 (O5) – N sp^2 from HIS 58<br>- O sp^3 (O5) – O sp^3 from TYR 101	2.956 Å<br>3.660 Å<br>2.944 Å<br>3.104 Å<br>2.557 Å

**6b**	-51.59	0.06	THR 63; LYS 62; ASP 162; GLU 83; ARG 163; TYR 132; TRP 88; MET 128; GLY 56; HIS 56; PRO 57; ARG 222; GLY 59; GLN 125; TYR 172; ILE 97; GLU 225; MET 231; TYR 101; ILE 100; ARG 176.	- O sp^3 (O5) – O sp^3 from TYR 101<br>- O sp^3 (O5) – N sp^2 from HIS 58<br>- N sp^3 ($N6^1$) – N sp^2 from ARG 222<br>- N sp^3 ($N6^1$)– O sp^2 from SO4 A<br>- N sp^3 ($N6^1$)– O sp^2 from GLU 83<br>- N sp^2 ($N1^1$) – N sp^2 from ARG 163	2.538 Å<br>3.077 Å<br>2.880 Å<br>3.044 Å<br>2.720 Å<br>3.125 Å
**6c**	-38.45	0.02	TRP 88; ILE 97; ARG 222; MET 128; GLU 225; ILE 100; TYR 101; GLN 221; GLN 125; TYR 132; GLU 83; ASP 162; THR 63; ARG 163; LYS 62; HIS 58; GLY 59; LEU 217; PRP 57; GLY 56; ILE 235; MET 231; ARG 176; TYR 172.	- O sp^3 (O5) – O sp^3 from TYR 101<br>- O sp^3 (O5) – N sp^2 from HIS 58<br>- N sp^3 ($N6^1$) – N sp^2 from ARG 222<br>- N sp^3 ($N6^1$) – N sp^2 from ARG 222<br>- N sp^3 ($N6^1$)– N sp^2 from ARG 222<br>- N sp^3 ($N6^1$)– O sp^2 from GLU 83<br>- N sp^2 ($N1^1$) – N sp^2 from ARG 222	2.766 Å<br>3.321 Å<br>2.649 Å<br>3.322 Å<br>3.076 Å<br>2.903 Å<br>3.151 Å
**6d**	-39.94	0.13	THR 63; LYS 62; GLU 83; ASP 162; TRP 88; ARG 222; GLU 225; GLY 59; ILE 97; THR 96; HIS 58; ILE 100; TYR 101; MET 231; ARG 176; TYR 172; GLN 125; PRO 57; MET 128; ARG 163; TYR 132.	- O sp^3 (O5) – O sp^3 from TYR 101<br>- O sp^3 (O5) – N sp^2 from ARG 176<br>- N sp^3 ($N6^1$) – O sp^3 from GLU 83<br>- N sp^3 ($N6^1$)– N sp^2 from ARG 222<br>- N sp^3 ($N6^1$)– N sp^2 from ARG 222	2.951 Å<br>2.963 Å<br>2.966 Å<br>3.365Å<br>3.044 Å
**6e**	-55.54	0.29	ASP 162; GLU 83; ARG 163; THR 63; LYS 62; TYR 132; MET 85; ALA; 167; GLY 129; TRP 88; ALA 168; MET 128; ALA 168; GLN 125; THR 96; ILE 97; PRO 173; ARG 222; HIS 58; PRO 57; GLY 59; TYR 101; GLU 225; MET 121; ALA 175; MET 231; ARG 176; MET 182; ILE 100; PRO 173.	- O sp^3 (O5) – O sp^2 from GLU 83<br>- O sp^3 (O5) – N sp^2 from ARG 222<br>- N sp^3 ($N1^1$) – O sp^3 from TYR 101<br>- N sp^3 ($N6^1$)– N sp^2 from ARG 176<br>- N sp^3 ($N6^1$)– N sp^2 from ARG 176<br>- O sp^3 ($O1^2$) – O sp^2 from TYR 172<br>- O sp^3 ($O1^2$) – O sp^2 from GLN 125<br>- O sp^3 ($O1^2$) – O sp^2 from GLN 125	2.590 Å<br>2.912 Å<br>2.932 Å<br>2.937 Å<br>3.015 Å<br>2.961 Å<br>2.680 Å<br>2.878 Å
**6f**	-26.86	0.02	MET 121; ALA 124; GLN 125; MET 128; ARG 176; PRO 173; ALA 168; TYR 172; ALA 168; TYR 172; TYR 132; ALA 167; ARG 163; PRO 57; HIS 58; GLU 83; GLY 59; LYS 62; ARG 222; GLU 225; MET 231; ILE 97; TYR 101; ILE 100; TRP 88	- O sp^3 (O5) – N sp^2 from ARG 222<br>- O sp^3 (O5) – N sp^2 from ARG 222<br>- O sp^3 (O5) – O sp^2 from SO4 A<br>- N sp^2 ($N1^1$)– N sp^2 from ARG 176<br>- N sp^2 ($N3^1$)– O sp^3 from TYR 101	2.952 Å<br>2.037 Å<br>2.695 Å<br>3.288 Å<br>2.557 Å
**6g**	-19.26	0.02	LYS 32; HIS 58; GLY 59; MET 231; LEU 217; ARG 220; GLN 221; ARG 222; GLU 225; TYR 101; ILE 100; ILE 97; TRP 88; ALA 124; MET 128; GLN 125; GLY 129; ARG 176; GLU 83; PRO 173; TYR 172; ALA 168; TYR 132; LEU 169; ARG 163; ALA 167; MET 85.	- O sp^3 (O5) – N sp^2 from ARG 222<br>- O sp^3 (O5) – O sp^2 from GLU 225<br>- O sp^3 (O5) – O sp^2 from GLU 225<br>- N sp^3 ($N6^1$)– O sp^2 from GLN 125	3.218 Å<br>3.315 Å<br>2.992 Å<br>3.135 Å
**6h**	-32.12	0.47	TRP 88; GLU 83; TYR 132; THR 63; ARG 222; MET 128; ILE 97; GLU 225; ILE 100; GLN 125; TYR 101; ARG 163; LYS 62; HIS 58; GLY 59; TYR 172; PRO 57; ARG 176; MET 231; ILE 235.	- O sp^3 (O5) – O sp^3 from TYR 101<br>- O sp^3 (O5) – N sp^2 from HYS 58<br>- N sp^2 ($N1^1$)– N sp^2 from ARG 222<br>- O sp^3($O4^2$) – N sp^2 from ARG 163<br>- O sp^3($O4^2$) – N sp^2 from ARG 163	2.504 Å<br>3.028 Å<br>3.363 Å<br>2.658 Å<br>3.393 Å

				- O sp^3(O4[2]) – N sp^3 from LYS 62	2.835 Å
**6i**	-70.21	0.09	TYR 239; ASP 55; PRO 57; HIS 164; ALA 167; ARG 163; LYS 62; TYR 172; PRO 173; ALA 168; ARG 176; TYR 132; GLY 129; GLN 125; MET 128; MET 121; ALA 124; TRP 88; ILE 100; ILE 97; TYR 101; GLU 225; ARG 222, MET 85; GLU 83; MET 85; MET 231.	- N sp^2 (N1[1]) – N sp^2 from ARG 176<br>- N sp^2 (N2[1]) – O sp^3 from TYR 101<br>- O sp^3 (O5) – N sp^2 from ARG 222<br>- O sp^3 (O5) – N sp^2 from ARG 222<br>- O sp^3 (O5) – O sp^2 from SO4 A<br>- O sp^3 (*p*-OH) – O sp^3 from TYR 132<br>- O sp^3 (*p*-OH) – O sp^2 from ARG 163	3.125 Å<br>2.502 Å<br>3.188 Å<br>3.033 Å<br>2.802 Å<br>3.229 Å<br>3.069 Å
**6j**	-62.08	1.57	GLY 59; GLU 83; ASP 55; HIS 58; ARG 163; ARG 222; METV85; PRO 57; HIS 164; TYR 132; TYR 239; ALA 167; GLU 225; TRP 88; ILE 92; MET 128; MET 231; TYR 172; ALA 168; GLY 129; TYR 101; ILE 100; ALA 124; GLN 125; PRO 173; ARG 176.	- N sp^2 (N1[1]) – O sp^3 from TYR 101<br>- N sp^2 (N2[1]) – N sp^2 from ARG 176<br>- O sp^3 (O5) – N sp^2 from ARG 222<br>- O sp^3 (O5) – N sp^2 from ARG 222<br>- O sp^3 (O5) – O sp^2 from SO4 A<br>- O sp^3 (*p*-CH_3O) – O sp^3 from TYR 132	2.499 Å<br>3.201 Å<br>3.048 Å<br>3.136 Å<br>2.773 Å<br>3.169 Å
**6k**	-70.07	0.07	LYS 62; GLY 59; HIS 58; PRO 57; ARG 163; MET 231; AEG 222; GLU 225; GLU 83; MET 85; ILE 97; TRP 88; TYR 101; TYR 132; ILE 100; ALA 168; ALA 167; TYR 172; ARG 176; PRO 173; ALA 124; MET 121; GLN 125; MET 128; GLY 129.	- N sp^2 (N2[1]) – N sp^2 from ARG 176<br>- N sp^2 (N[1]) – O sp^3 from TYR 101<br>- O sp^3 (O5) – N sp^2 from ARG 222<br>- O sp^3 (O5) – N sp^2 from ARG 222<br>- O sp^3 (O5) – O sp^2 from SO4 A	3.215 Å<br>2.553 Å<br>2.748 Å<br>3.333 Å<br>2.944 Å
**7a**	-57.27	0.02	TYR 132; TRP 88; MET 128; GLN 125; ASP 162; GLU 83; ARG 163; THR 63; LYS 62; HIS 58; ARG 222; GLY 59; GLU 225; MET 231; TYR 101; ARG 176; ILE 100; ILE 97; TYR 172.	- O sp^3 (O6[1]) – N sp^3 from LYS 62<br>- O sp^3 (O6[1]) – N sp^2 from ARG 222<br>- N sp^2 (N1[1]) – N sp^2 from ARG 163<br>- O sp^3 (O5) – N sp^2 from HIS 58<br>- O sp^3 (O5) – O sp^3 from TYR 101	3.314 Å<br>3.095 Å<br>2.916 Å<br>3.109 Å<br>2.561 Å
**7b**	-53.11	0.55	THR 63; LYS 62; ASP 162; GLU 83; TYR 132; HIS 58; ARG 132; TRP 88; MET 428; TYR 172; GLN 125; ARG 176; MET 231; TYR 101; ILE 100; ILE97; GLU 225.	- O sp^3 (O6[1]) – N sp^3 from ARG 222<br>- O sp^3 (O6[1]) – N sp^2 from ARG 163<br>- N sp^2 (N1[1]) – N sp^2 from ARG 163<br>- O sp^3 (O5) – N sp^2 from HIS 58<br>- O sp^3 (O5) – O sp^3 from TYR 101	3.358 Å<br>3.224 Å<br>3.034 Å<br>3.080 Å<br>2.540 Å

[1] refers to the atoms in the heterocyclic base; [2]refers to the atoms in the substituted radical at the N^6 atom or O^6
